# Zintl Ions and Phases Promote the Catalytic Hydrophosphination of Alkynes, Alkenes, and Imines

**DOI:** 10.1021/acs.organomet.3c00494

**Published:** 2024-01-26

**Authors:** Benjamin L. L. Réant, Meera Mehta

**Affiliations:** Department of Chemistry, University of Manchester, Oxford Road, Manchester M13 9PL, U.K.

## Abstract

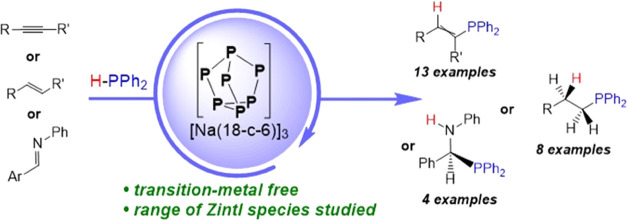

Although Zintl ions and phases have been known for more than a century, their application as tools to build organic molecules is underdeveloped. Here, a range of Zintl ions and phases were surveyed in the hydrophosphination of alkynes, alkenes, and imines with diphenylphosphine to afford useful organophosphine products. Further investigations with diphenylphosphine in the absence of the unsaturated organic substrates revealed the formation of the diphenylphosphide anion, allowing for the conclusion that the role of the Zintl species is as an initiator in these transformations.

## Introduction

Organophosphines, compounds that feature P–C bonds, are ubiquitous as ligands in coordination chemistry, organocatalysts, and substrates in organic transformations and are widely employed as pharmaceuticals, fertilizers, and flame retardants.^1^ Catalyzed formation of organophosphines *via* the addition of P–H bonds across an unfunctionalized moiety was first reported in 1990 by Pringle and Smith using a platinum system.^2^ Since then, hydrophosphination reactions have flourished as a key strategy for their synthesis.^3,4^ Often, these transformations require a catalyst, although, recently, a few “catalyst-free” protocols have also emerged, albeit requiring higher temperatures.^5^ Efforts to move toward greener synthetic practices have promoted innovation toward protocols mediated with main group catalysts, where the main group elements tend to have greater crustal abundance and lower costs compared to their transition metal counterparts. In 2002, Knochel and co-workers reported the hydrophosphination of alkenes catalyzed by 20 mol % potassium *tert*-butoxide.^6^ Later in 2007, Barrett and Hill reported a β-diketiminato Ca complex that mediated intermolecular alkene hydrophosphination.^7^ In 2018, the Webster group used 10 mol % potassium hexamethyldisilazane ([K{N(SiMe_3_)_2_}]) for the bis-hydrophosphination of alkynes to generate 1,1-diphosphanes.^8^ Later, Webster also reported a [GeCl{N(SiMe_3_)_2_}_3_] compound as a precatalyst to mediate the hydrophosphination of styrenes and internal alkynes with anti-Markovnikov selectivity.^9^ Meanwhile, Mulvey utilized a 10 mol % lithium phosphidoaluminate complex (^i^Bu_3_AlPPh_2_Li(THF)_3_) to promote the hydrophosphination of alkynes, alkenes, and carbodiimides.^10^ Most recently, in 2022, Mulvey further reported a series of solvated and sequestered sodium diphenylphosphide complexes and their catalytic competency in the hydrophosphination of alkynes and alkenes.^11^

**Figure 1:**
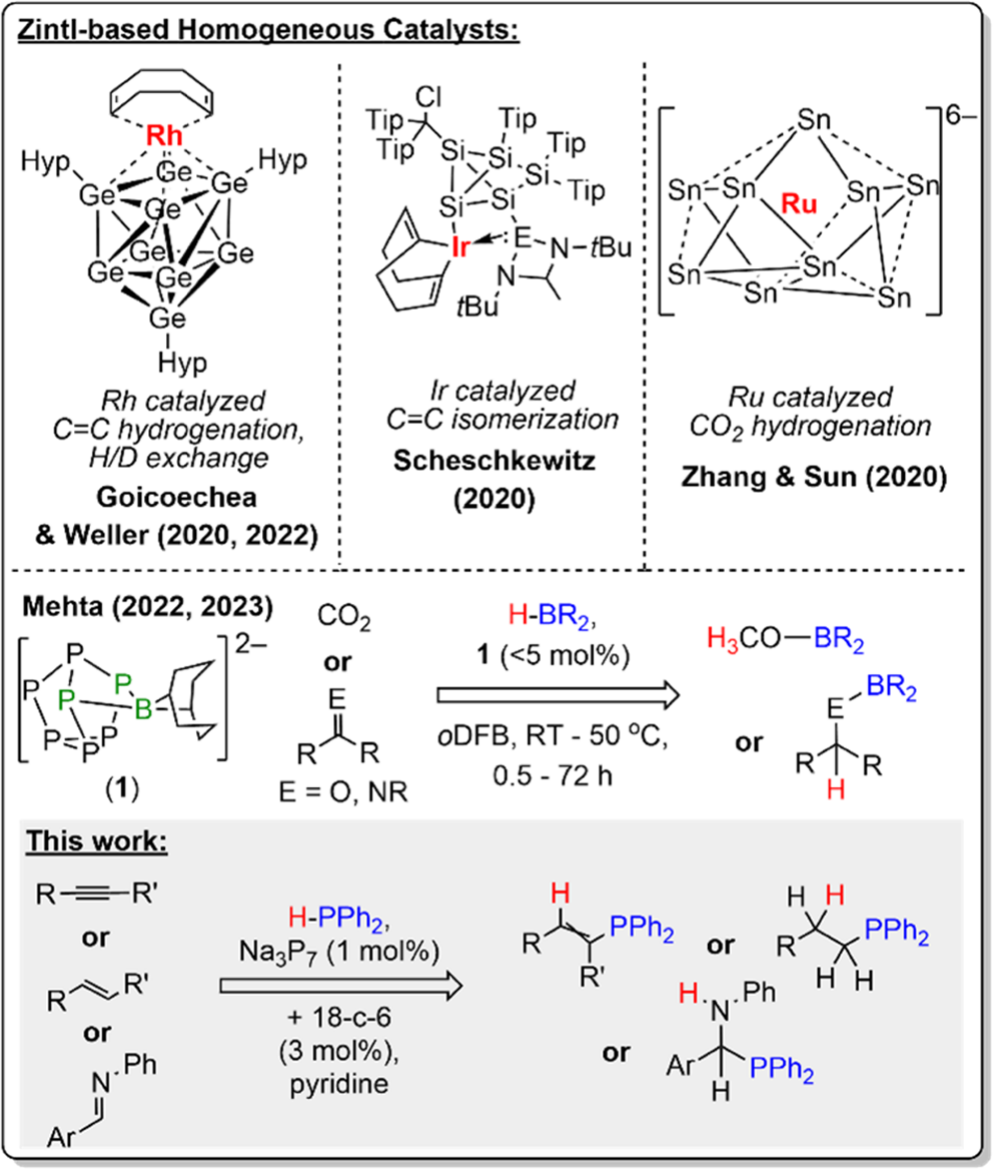
Select examples of Zintl-derived catalysts and this work. Hyp = Si(SiMe_3_)_3_ and Tip = 2,4,6-triisopropylphenyl.

Zintl ions and phases have been known for over a century but remained largely ignored. Their chemistry is now experiencing a renaissance,^12^ with developments as ligands in coordination chemistry, which have unlocked unique physical properties,^13^ in small molecule activations including reactivity akin to frustrated Lewis pair chemistry,^14,15^ and as precursors to nanostructures and light-emitting diodes.^16^ One burgeoning area is the application of these ions and phases in organic catalysis. Within homogeneous catalysis (Figure 1), in 2020, Goicochea and Weller coordinated a deltahedral [Ge_9_] cluster to Rh, which facilitated the hydrogenation of cyclic alkenes, and later in 2022, the same system was found to scramble H/D.^17^ In contrast, Scheschkewitz and co-workers prepared a silicon cluster with coordinated iridium to mediate the isomerization of alkenes.^18^ Meanwhile, Zhang and Sun encapsulated Ru within a [Sn_9_] cluster, dispersed it on a CeO_2_ surface, and found that it mediated the reverse water-shift reaction.^19^ In 2022, we reported the first example of Zintl transition metal-free catalysis,^20^ when the boron-functionalized cluster [Na(18-c-6)]_2_[(BBN)P_7_] (**1**) (also studied in this report) was found to facilitate the reduction of CO_2_ to a methoxyborane. Later, we reported the same cluster as an active catalyst for the hydroboration of pyridines, imines, and nitriles.^21^ We also prepared the boron-tethered [P_7_] cluster where the cluster and boron are linked via an aliphatic unit to mediate hydroboration catalysis; however with this system, the cluster is an innocent platform for boron catalysis.^22^ As for transition metal-free heterogeneous Zintl catalysis, hydrogenation of alkynes has been the most widely explored. In 2019, Goldberger and co-workers utilized BaGa_2_ to encapsulate dihydrogen (BaGa_2_H_2_) and mediate the reduction of phenylacetylene.^23^ Following on from this work, they reported a 13-layer trigonal polytype consisting of [GaGe]^2–^ ions separated by Ca^2+^, CaGaGe, in the same reduction.^24^ Most recently, Goldberger and co-workers have also found that Ga/In layered phases are competent in alkyne hydrogenation reactions.^25^

Here, we find that the transition metal-free Zintl cluster [Na(18-c-6)]_2_[(BBN)P_7_] (**1**; 18-c-6 = 1,4,7,10,13,16-hexaoxycyclooctadecane {18-crown-6}, BBN = 9-borabicyclo[3.3.1]nonane) promotes the hydrophosphination of alkynes. We also found that the unfunctionalized Zintl ion [Na(DME)_*x*_]_3_[P_7_] (DME = 1,2-dimethoxyethane) demonstrated greater reactivity when compared to **1**, and we further expanded into the hydrophosphination of alkenes and imines. This led to the examination of the K_3_P_7_, K_3_As_7_, K_3_Sb_7_, K_5_Bi_4_, K_4_Ge_9_, and K_4_Sn_9_ phases in hydrophosphination chemistry. Further investigations revealed that MPPh_2_ (M = Na, K) was generated in the reaction mixture, and thus, we postulate that these ions and phases are acting as initiators rather than catalytic participants.^11^ To the best of our knowledge, this work represents the first survey of Zintl ions and phases in hydrophosphination catalysis.

## Results and Discussion

### Initial Investigations

Following our previously reported synthesis, [Na(18-c-6)]_2_[(BBN)P_7_] (**1**) was prepared by dehydrocoupling the 9-borabicyclo[3.3.1]nonane dimer (HBBN dimer) with [Na(18-c-6)]_2_[HP_7_] salt.^20^ Next, with 10 mol % **1** and equimolar amounts of phenylacetylene (**2a**) and diphenylphosphine (HPPh_2_), the hydrophosphination reaction was tested in a variety of solvents (e.g., C_6_D_6_, THF, *o*DFB, and pyridine) at both room temperature (RT) and 50 °C, with results summarized in Table 1. Diphenylphosphine was selected as the hydrophosphination agent as it has shown the greatest precedence in this transformation.^3^ We found that the highest conversions were obtained when pyridine was employed as the solvent, with >90% conversion after 24 h (h) at both RT and 50 °C. In line with literature precedence, both *trans-* (**3a**) and *cis*- (**4a**) alkene products were obtained,^26^ with a preference toward the *cis*-product (**4a**). *Cis*- and *trans*-isomers were assigned by comparing to literature known NMR spectroscopic signals.^11^ Control reactions further confirmed that **2a** does not undergo hydrophosphination with HPPh_2_ in the absence of a catalyst, see SI Section 2.1.

**Table 1:**
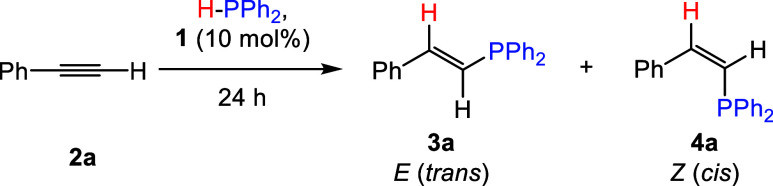
Solvent Screening for Hydrophosphination of Phenylacetylene (**2a**)

entry	solvent	*T* (°C)	conversion/%^a^	distribution (**3a:4a**)
1	C_6_D_6_	RT	9	2:7
2		50	8	4:4
3	THF	RT	3	2:1
4		50	20	16:4
5	*o*DFB	RT	15	7:8
6		50	19	10:9
7	pyridine	RT	91	17:74
8		50	93	20:73

a Conversion determined by ^31^P{^1^H} NMR spectroscopy.

To further understand the role of **1** in this transformation, other clusters were investigated for the hydrophosphination of **2a** in pyridine (Table 2). Both the neutral silyl functionalized cluster (Me_3_Si)_3_P_7_ and the unfunctionalized [Na(DME)_*x*_]_3_[P_7_] (**A**) cluster were tested at 10 mol % loading. (Me_3_Si)_3_P_7_ showed no catalytic competency, while **A** gave complete conversion within 24 h with 16% of the *trans*-isomer **3a** and 84% of the *cis*-isomer (**4a**) being generated. Further, to test if the boron component of **1** is independently active, the HBBN dimer was tested and found to be catalytically inactive toward the hydrophosphination of **2a**. For the [Na(18-c-6)]^+^ fragment, both sodium triflate (NaOTf) and 18-crown-6 (18-c-6) were independently investigated, and neither showed any catalytic competency, confirming that the presence of the phosphide anion is vital to establishing catalytic turnover and that the unfunctionalized [Na(DME)_*x*_]_3_[P_7_] cluster (**A**) outperforms **1**. It is also noteworthy that phosphines diisopropylphosphine (as a hexane solution) and diphenylphosphine oxide were also tested with 3.33 mol % **A** and **2a** in pyridine and gave no conversion to the hydrophosphinated products. Further, no evidence for bis-hydrophosphination was observed when 2 equiv of diphenylphosphine were employed.

**Table 2:**
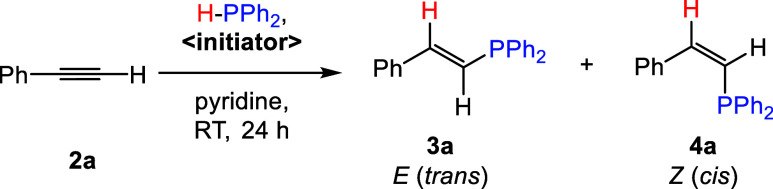
Control Reactions for the Hydrophosphination of Phenylacetylene (**2a**)

entry	initiator	loading (mol %)	conversion/%^a^ (**3a**:**4a**)
1	(HBBN)_2_	5	0
2	(Me_3_Si)_3_P_7_	10	0
3	[Na(DME)_*x*_][P_7_] (**A**)	10	>99 (16:84)
4	NaOTf	10	0
5	18-c-6	30	0

a Conversion determined by ^31^P{^1^H} NMR spectroscopy.

[Na(DME)_*x*_]_3_[P_7_] (**A**) can be readily synthesized on a large scale (40 g), and the resulting product can vary in its DME content depending on how thoroughly the solvent is removed after isolation. Through elemental analysis, the DME content was determined to be 0.05/Na cation, [Na(DME)_0.05_]_3_[P_7_]. As [Na(DME)_0.05_]_3_[P_7_] (**A**) demonstrated greater reactivity in the hydrophosphination reactions when compared to **1**, the solvent system for the hydrophosphination of **2a** with HPPh_2_ using 10 mol % **A** and 30 mol % 18-c-6 was reassessed (see SI, Section 2.5). Again, the greatest conversion was obtained when pyridine was employed as the solvent. Consistent with our previous report, when the unfunctionalized [Na(DME)_*x*_]_3_[P_7_] cluster was tested in the hydroboration of pyridines, imines, and nitriles,^21^ addition of 18-c-6 improved conversion with complete consumption of **2a** obtained within 15 min (12 h without 18-c-6). We postulate that the cation sequestering agent increases the solubility of the anionic species, increasing the effective loading in solution.

When investigating NaPPh_2_ as a catalyst, Mulvey and co-workers uncovered that the distribution of *trans*- *vs cis*-products was partially dependent on the cation sequestering agent employed. Thus, the hydrophosphination of **2a** with HPPh_2_ in pyridine catalyzed by 3.33 mol % **A** was investigated with both 10 mol % 15-crown-5 and 2.2.2-cryptand (Table 3). Analysis of the reaction mixtures by ^31^P{^1^H} NMR spectroscopy revealed complete conversion within 15 min with **3a**:**4a** distributions of 16:84 and 17:83 for the 15-crown-5 and 2.2.2-cryptand reactions, correspondingly. The influence of the cation sequestering agent was less significant when using **A** compared to the diphenylphosphide complexes reported by Mulvey.^11^ We found that the equivalence of 18-c-6 to **A** made little impact on the distribution of products or time required to hydrophosphinate **2a** with HPPh_2_ (see SI Section 2.7). Catalyst loading of **A** could be further reduced to 1 mol % (+ 3 mol % 18-c-6, see SI Section 2.8), and complete hydrophosphination of **2a** achieved after 15 min, with a *trans*- (**3a**)/*cis*- (**4a**) product distribution of 15:85.

**Table 3:**
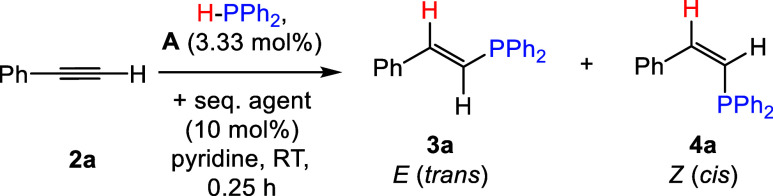
Different Cation Sequestering Agents for the Hydrophosphination of Phenylacetylene (**2a**)

entry	sequestering agent	conversion/%^a^ (**3a**:**4a**)
1	15-crown-5	>99 (16:84)
2	2.2.2-cryptand	>99 (17:83)

a Conversion determined by ^31^P{^1^H} NMR spectroscopy.

### Alkyne Scope

Having demonstrated the capability of **A** to promote alkyne hydrophosphination, the scope of alkynes was expanded with 1 mol % **3**, 3 mol % 18-crown-6, and diphenylphosphine in pyridine (Table 4). *Para*-substituted phenylacetylenes 4-ethynylanisole (**2b**), 4-ethynyltoluene (**2c**), 4-trifluoromethylphenylacetylene (**2d**), 4-fluorophenylacetylene (**2e**), 4-chlorophenylacetylene (**2f**), 4-bromophenylacetylene (**2g**), and 4-ethynylaniline (**2h**) could all be hydrophosphinated with high conversions, between 82 and >99%, obtained at RT in under 2 h. Next, the *meta*- and *ortho*-functionalized phenylacetylenes **2i** and **2j** were investigated, and again complete conversion to the hydrophosphinated products was obtained after 15 min at RT. Consistent with the hydrophosphination of **2a**, in almost all cases, both the *trans*- and *cis*-products were observed, with a preference toward the *cis*-hydrophosphinated product. In the cases of 4-ethynylanisole (**2b**), 4-fluorophenylacetylene (**2e**), and 4-ethynylaniline (**2h**) hydrophosphination, there was high selectivity toward the *cis*-isomer, with over 95% conversion to exclusively this product. Meanwhile, the aliphatic substituted alkynes cyclohexylacetylene (**2k**) and 1-hexyne (**2l**) could be hydrophosphinated in high conversions but required long reaction times of up to 2 days and higher temperatures of 50 °C. Finally, the internal alkyne diphenylacetylene (**2m**) was investigated, and complete conversion was obtained at 50 °C after 4 h. Interestingly, in the case of **2m** hydrophosphination, analysis of the reaction mixture by ^31^P{^1^H} NMR spectroscopy now revealed selectivity toward the *trans*- product. 4-Ethynylbenzaldehyde, trimethylsilylacetylene, bis-trimethylsilylacetylene, and 3-chloro-1-phenyl-1-propyne were also investigated as the alkyne substrate, but no conversion to the hydrophosphinated products was observed under similar reaction conditions. Finally, to demonstrate the scalability of this process, we carried out the hydrophosphination of **2h** with an equimolar amount of HPPh_2_ on a 5 mmol scale; following extraction of the filtrate in dichloromethane, product **4h** was cleanly isolated in a 78% yield as a pale yellow powder (see SI Section 4.1). Hydrophosphination of substrates **2a**, **2c**, and **2d** was also previously studied by Mulvey and co-workers using [Na(15-c-5)][PPh_2_] as a catalyst. Significant differences in conversion to products were not observed; however, we observed greater selectivity for the *cis*-isomer, except in the case of **2m** where selectivity for the *trans-*isomer was increased.^11^

**Table 4:**
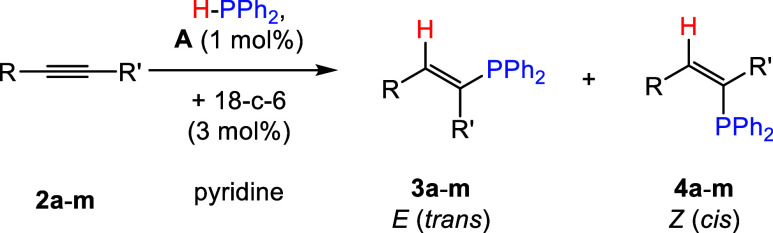
Catalytic Hydrophosphination of Alkynes

entry	substrate	*T* (°C)	*t* (h)	% Conv.^a^ (**3**:**4**)
1	**2b**; R = *p*-MeOPh, R′ = H	RT	1	>99 (2:98)
2	**2c**; R = *p*-MePh, R′ = H	RT	1	82 (6:76)
3	**2d**; R = *p*-CF_3_Ph, R′ = H	RT	0.25	>99 (31:69)
4	**2e**; R = *p*-FPh, R′ = H	RT	0.25	>99 (5:95)
5	**2f**; R = *p*-ClPh, R′ = H	RT	0.5	91 (26:65)
6	**2g**; R = *p*-BrPh, R′ = H	RT	0.5	90 (27:62)
7	**2h**; R = *p*-NH_2_Ph, R′ = H	RT	2	>99 (0:100)
8	**2i**; R = *m*-MePh, R′ = H	RT	0.25	>99 (13:87)
9	**2j**; R = *o*-MePh, R′ = H	RT	0.75	>99 (16:84)
10	**2k**; R = Cy, R′ = H	50	24	>99 (32:68)
11	**2l**; R = ^*n*^Bu, R′ = H	50	48	96 (47:49)
12	**2m**; R = R′ = Ph	50	4	>99 (60:40)

a Conversion determined by ^31^P{^1^H} NMR spectroscopy.

### Alkene Scope

Next, alkene hydrophosphination was probed with 1 mol % **A**, 3 mol % 18-c-6, and HPPh_2_ in pyridine (Table 5). First, the hydrophosphination of styrene (**5a**) was investigated, and it was found to fully convert to the alkyl-phosphine product **6a** after 1 h at RT. Further, *para*-substituted styrenes 4-methoxystyrene (**5b**), 4-methylstyrene (**5c**), 4-fluorostyrene (**5d**), and 4-bromostyrene (**5e**) could all be hydrophosphinated to products **6b**–**6e** in complete conversion. For 4-methoxystyrene (**5b**), this conversion was achieved in 24 h, while for substrates **5c**–**5e**, the reactions required less than 12 h. Meanwhile, the hydrophosphination of 2-vinylnapthalene (**5f**) gave only **6f** in 9% conversion after 24 h at elevated temperatures (50 °C). However, triethoxyvinylsilane (**5g**) could be readily hydrophosphinated to product **6g** in >99% conversion after 9 h at RT. The internal alkene *trans*-stilbene (**5h**) was also investigated, and it was found to afford **6h** in 34% conversion after 24 h at 50 °C. Efforts were also made to hydrophosphinate 9-vinyl-9*H*-carbozole and 1-octene; however, no reaction was observed by NMR spectroscopy even after 24 h at 50 °C. Finally, as proof-of-concept scaled reactions, we carried out the hydrophosphination of alkenes **5a**, **5d**, **5e**, and **5g** with an equimolar amount of HPPh_2_ on a 5 mmol scale. Similarly, the hydrophosphination of **5b** and **5c** was scaled to 2.4 and 1.6 mmol, respectively. In all cases, following an extraction in dichloromethane, the products were cleanly isolated as colorless oils and yields between 50–81% were obtained (see SI Section 4).

**Table 5:**
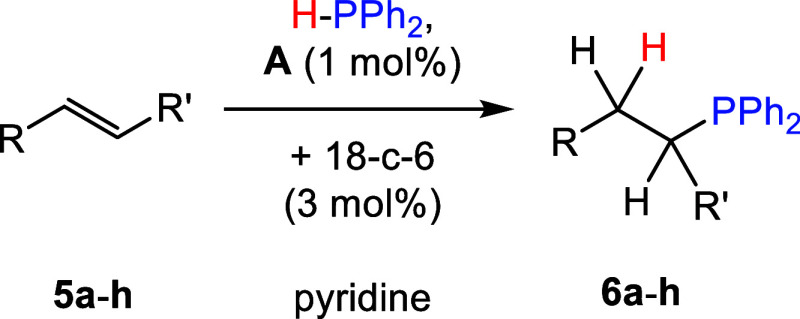
Catalytic Hydrophosphination of Alkenes

entry	substrate	*T* (°C)	*t* (h)	% Conv.^a^
1	**5a**; R = Ph, R′ = H	RT	1	>99
2	**5b**; R = *p*-MeOPh, R′ = H	RT	24	>99
3	**5c**; R = *p*-MePh, R′ = H	RT	6	>99
4	**5d**; R = *p*-FPh, R′ = H	RT	12	>99
5	**5e**; R = *p*-BrPh, R′ = H	RT	1	>99
6	**5f**; R = Napth, R′ = H	50	24	9
7	**5g**; R = (EtO)_3_Si, R′ = H	RT	9	>99
8	**5h**; R = R′ = Ph	50	24	34

a Conversion determined by ^31^P{^1^H} NMR spectroscopy.

### Imine Scope

Finally, we explored imine hydrophosphination reactions with HPPh_2_, 1 mol % **A**, and 3 mol % 18-c-6 in pyridine (Table 6). Control reactions revealed that *N*-benzylideneaniline (**7a**) could be hydrophosphinated in the absence of a catalyst in C_6_D_6_ (20% conversion), THF (18% conversion), and *o*DFB (74% conversion) after 24 h but not in pyridine after the same amount of time. However, in the presence of the catalyst, benzylideneaniline (**7a**) was fully hydrophosphinated to **8a** after 15 min in pyridine. Similarly, *para*-substituted methoxy (**7b**), methyl (**7c**), and bromo (**7d**) functionalized *N*-benzylideneanilines could also be hydrophosphinated to products **8b**–**8d** in high conversion (>89%) within 1 h.

**Table 6:**
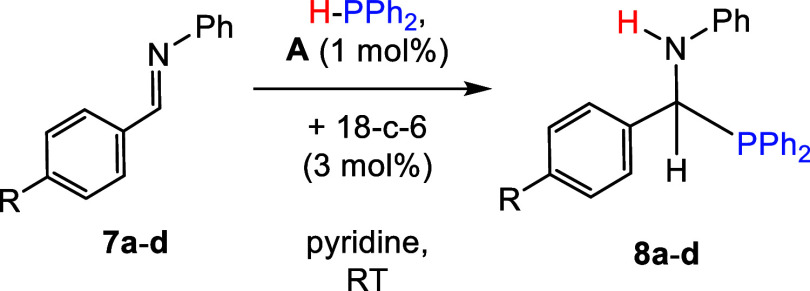
Catalytic Hydrophosphination of Imines

entry	substrate	*t* (h)	% Conv.^a^
1	**7a**; R = H	0.25	>99
2	**7b**; R = MeO	0.25	>99
3	**7c**; R = Me	1	>99
4	**7d**; R = Br	0.25	89

a Conversion determined by ^31^P{^1^H} NMR spectroscopy.

### Hydrophosphination with Other Zintl Ions/Phases

As the unfunctionalized cluster **A** was able to promote hydrophosphination reactions, other pnictogen and tetrel Zintl ions and phases were also investigated in this transformation. Zintl ions [K(DME)_*x*_]_3_[P_7_] (**B**) and [K(DME)_*x*_]_3_[As_7_] (**C**) can both be synthesized by similar methods to **A**.^15,27^ The DME content was similarly determined by elemental analysis and found to be 0.03 and 0.06/K cation for **B** ([K(DME)_0.03_]_3_[P_7_]) and **C** ([K(DME)_0.06_]_3_[As_7_]), respectively. Further, a range of Zintl phases, namely, K_3_P_7_ (**D**),^28^ K_3_Sb_7_ (**E**),^29^ K_5_Bi_4_ (**F**),^30^ K_4_Ge_9_ (**G**),^31^ and K_4_Sn_9_ (**H**),^31^ were prepared following literature known methods. It is worth noting that K_3_P_7_ (**D**) was prepared so as to compare the phase to the solution-state synthesized and DME coordinated ion ([K(DME)_0.03_]_3_[P_7_] **B**). As a representative transformation to assess the relative reactivity of these ions and phases, the hydrophosphination of phenylacetylene (**2a**) with HPPh_2_ in pyridine was tested. Further, as the pnictogen systems have either a 3– or 5– charge while the tetrel systems feature a 4– charge, the catalyst loading was normalized to 10 mol % anionic sites in the reaction mixture. Additionally, the ions and phases were investigated in the presence and absence of the cation sequestering agent 18-c-6, with results summarized in Table 7.

**Table 7:**
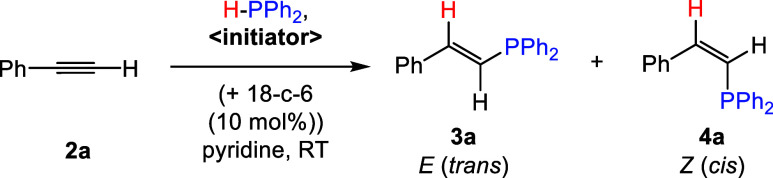
Catalytic Hydrophosphination of Phenylacetylene (**2a**) with Various Zintl Ions/Phases

entry	initiator	loading (mol %)	*t* (h)	% Conv.^a^ (**3a**:**4a**)
1	**A**	3.33	12	98 (13:85)
2	**A** + 18-c-6		0.25	>99 (17:83)
3	**B**	3.33	6	98 (13:85)
4	**B** + 18-c-6		0.25	>99 (14:86)
5	**C**	3.33	1	>99 (14:86)
6	**C** + 18-c-6		0.25	>99 (19:81)
7	**D**	3.33	18	98 (13:86)
8	**D** + 18-c-6		0.25	>99 (17:83)
9	**E**	3.33	24	25 (3:22)
10	**E** + 18-c-6		24	86 (13:73)
11	**F**	2	1	>99 (23:77)
12	**F** + 18-c-6		0.25	>99 (16:84)
13	**G**	2.5	24	23 (3:20)
14	**G** + 18-c-6		1	>99 (14:86)
15	**H**	2.5	1	>99 (18:82)
16	**H** + 18-c-6		0.25	>99 (14:86)

a Conversion determined by ^31^P{^1^H}NMR spectroscopy.

As expected, similar to the investigations with **A** mentioned above, addition of 18-c-6 increased the conversion or rate of conversion from **2a** to **3a**/**4a** in all cases when 2–3.33 mol % of **B**–**H** were employed. In the case of arsenic and phosphorus systems **B**–**D**, complete conversion was obtained after 15 min, consistent with the reactivity of **A**. In contrast, for K_3_Sb_7_ (**E**) with 18-c-6, a conversion of 86% to **3a** and **4a** was obtained after 24 h. Across all reactions, there was little variance in the distribution of products observed (*ca.* 1:5 **3a**:**4a**). The heaviest pnictogen system K_5_Bi_4_ (**F**) and the one with the highest anionic charge gave complete conversion to the hydrophosphinated products **3a**:**4a** after 1h in the absence of 18-c-6 and after 15 min in the presence of 18-c-6. Investigation of the tetrel phases revealed that K_4_Ge_9_ (**G**) only gave 23% conversion after 24 h to products **3a** and **4a** in the absence of 18-c-6, but in the presence of 18-c-6, this conversion was increased to >99% after 1 h. However, the related K_4_Sn_9_ (**H**) system could rapidly facilitate the complete hydrophosphination of **2a** within 1 h, even in the absence of 18-c-6.

### Mechanistic Insight

In order to further investigate the role of [Na(DME)_0.05_]_3_[P_7_] (**A**) in promoting hydrophosphination reactions, reaction mixtures of **A** with 3 equiv of 18-c-6 were allowed to react with 3 equiv of either HPPh_2_, **2a**, **5a**, or **7a** in pyridine. For the reaction with HPPh_2_, monitoring the reaction by ^31^P{^1^H} NMR spectroscopy revealed unreacted HPPh_2_ as well as the immediate formation of the [HP_7_]^2–^ dianion, with two resonances at −23.8 and −110.6 ppm, which were in good agreement with literature reports (δ_P_ (*d*_7_-DMF) = −23.7, −111.4 ppm).^32^ Additionally a doublet at 13.6 ppm (^1^*J*_PP_ = 323 Hz) and a related triplet at −98.2 ppm were also observed, corresponding to the known [Na(18-c-6)][P(PPh_2_)_2_] complex with the cation sequestered.^33^ And another singlet at −52.2 ppm from [NaPPh_2_] without the cation sequestered was also detected.^34^ Similar metal phosphide generation could be observed in reactions between **B–H** and HPPh_2_, where the ratio between the phosphine and anionic sites was 1:1 (see SI Section 6). For the reactions with substrates **5a** and **7a**, no reaction was observed, even after 24 h. However, in the case of **2a**, although no immediate reaction was observed after 3 h, three singlet resonances grew in the ^31^P{^1^H} NMR spectrum at −48.1, −58.3, and −71.9 ppm, which remained stable after further monitoring for 24 h. The absence of multiplicity or broadness suggests the absence of P–P coupling and is consistent with cluster decomposition. This decomposition with **2a** is notably slower than cluster reactivity with HPPh_2_ to generate the phosphide anion, and as alkyne hydrophosphinations are completed within 1 h, it is not expected to be significant on the catalytic time scale. Based on these experiments, we believe that the Zintl ions and phases act as initiators in these transformations, by accepting proton and generating diphenylphosphide ([MPPh_2_]; M = Na, K) as the active catalyst.^11^ In accordance with the previous work done by the Mulvey group,^11^ the diphenylphosphide generated is expected to attack the alkyne to produce an olefinic anion, which then accepts a proton to give the hydrophosphinated product. Although it is possible that [HP_7_]^2–^ supplies this proton regenerating [P_7_]^3–^ as a catalyst, the concentration of HPPh_2_ during catalysis would be 100 times higher. Thus, it is far more likely that this proton is supplied by another 1 equiv of HPPh_2_, regenerating the diphenylphosphide anion. We believe that the differences in conversions obtained by these initiators observed in Table 7 are a balance of their relative solubilities and abilities to accept protons. To further confirm that the role of the Zintl species is proton acceptance, 10 mol % pyridine hydrochloride was added to a reaction mixture with 3.33 mol % **A**, 10 mol % 18-c-6, HPPh_2_, and **2a** and found to give no reaction. The hydrochloride in this reaction serves to fully protonate the cluster, poisoning it from accepting protons from HPPh_2_ and turning off further phosphide-mediated catalysis.

## Conclusions

In conclusion, the reactivity of group 15 and group 14 Zintl ions and phases in promoting the hydrophosphination of alkynes was surveyed. The [Na(DME)_0.05_]_3_[P_7_] (**A**) was also investigated in the hydrophosphination of alkenes and imines. Further investigations between the Zintl ions and phases with HPPh_2_ revealed the formation of diphenylphosphide salts, which are believed to be the active catalysts. The application of Zintl ions and phases as tools in catalysis and organic synthesis is a new field. This work further affirms the utility of Zintl ions and phases in synthetic science.

## Experimental Section

Experimental details, including general procedures, synthetic methods, catalytic procedures, control reactions, characterization data, and preparation scale syntheses, are located in the Supporting Information.

## Supporting Information Available

General information, experimental procedures, and characterization data (PDF)
