# Supplementary material for: Zintl Ions and Phases Promote the Catalytic Hydrophosphination of Alkynes, Alkenes, and Imines
Source: Organometallics. 2024 Jan 26;43(3):395–401. doi: 10.1021/acs.organomet.3c00494 (PMC10865437; doi:10.1021/acs.organomet.3c00494)
Supplement: Supplementary file 1 — om3c00494_si_001.pdf [file om3c00494_si_001.pdf]

Supporting Information for

Zintl Ions and Phases Promote the Catalytic Hydrophosphination of Alkynes, Alkenes and Imines

Benjamin L. L. Réant,[a] Meera Mehta*[a]

[a] Department of Chemistry, University of Manchester, Oxford Road, Manchester, M13 9PL, United Kingdom.

* meera.mehta@manchester.ac.uk

Contents

1. Methods & Materials S4

1.1. Experimental Considerations S4

1.2. Analytical Considerations S4

1.3. Hydrophosphination Scope Procedure S5

2. Screening Conditions S6

2.1. Control Solvent Screening S6

2.2. Solvent screening for the hydrophosphination of phenylacetylene with $[Na(18\text{-}c\text{-}6)]_2[(BBN)P_7]$ (1) S7

2.3. Screening initiators for the hydrophosphination of phenylacetylene S10

2.4. Determining DME content of $[Na(DME)_x]_3[P_7]$ (A) S11

2.5. Solvent Screening for the hydrophosphination of phenylacetylene with A ... S12

2.6. Cation sequestering agent selection S13

2.7. Screening 18-crown-6 loadings S14

2.8. Screening Initiator Loading S17

3. Characterisation Data S19

3.1. Products of Alkyne Hydrophosphination S19

3.1.1. **3a**/**4a** – (*E*/*Z*)-diphenyl(styryl)phosphane S20

3.1.2. **3b**/**4b** – (*E*/*Z*)-(4-methoxystyryl)diphenylphosphane S21

3.1.3. **3c**/**4c** – (*E*/*Z*)-(4-methylstyryl)diphenylphosphane S22
3.1.4. **3d**/**4d** – (*E*/*Z*)-diphenyl(4-(trifluoromethyl)styryl)phosphane S23
3.1.5. **3e**/**4e** – (*E*/*Z*)-(4-fluorostyryl)diphenylphosphane S25
3.1.6. **3f**/**4f** – (*E*/*Z*)-(4-chlorostyryl)diphenylphosphane S27
3.1.7. **3g**/**4g** – (*E*/*Z*)-(4-bromostyryl)diphenylphosphane S28
3.1.8. **4h** – (*Z*)-4-(2-(diphenylphosphaneyl)vinyl)aniline S29
3.1.9. **3i**/**4i** – (*E*/*Z*)-(3-methylstyryl)diphenylphosphane S30
3.1.10. **3j**/**4j** – (*E*/*Z*)-(2-methylstyryl)diphenylphosphane S31
3.1.11. **3k**/**4k** – (*E/Z*)-(2-cyclohexylvinyl)diphenylphosphane S32
3.1.12. **3l**/**4l** – (*E*/*Z*)-hex-1-en-1-yldiphenylphosphane S34
3.1.13. **3m**/**4m** – (*E*/*Z*)-(1,2-diphenylvinyl)diphenylphosphane S35
3.2. Products of Alkene Hydrophosphination S36
3.2.1. **6a** – phenethyldiphenylphosphane S36
3.2.2. **6b** – (4-methoxyphenethyl)diphenylphosphane S37
3.2.3. **6c** – (4-methylphenethyl)diphenylphosphane S38
3.2.4. **6d** – (4-fluorophenethyl)diphenylphosphane S39
3.2.5. **6e** – (4-bromophenethyl)diphenylphosphane S41
3.2.6. **6f** – (2-(naphthalen-2-yl)ethyl)diphenylphosphane S42
3.2.7. **6g** – diphenyl(2-(triethoxysilyl)ethyl)phosphane S42
3.2.8. **6h** – (1,2-diphenylethyl)diphenylphosphane S44
3.3. Products of Imine Hydrophosphination S45
3.3.1. **8a** – *N*-((diphenylphosphaneyl)(phenyl)methyl)aniline S45
3.3.2. **8b** – *N*-((diphenylphosphaneyl)(4-methoxyphenyl)methyl)aniline S46
3.3.3. **8c** – *N*-((diphenylphosphaneyl)(p-tolyl)methyl)aniline S47
3.3.4. **8d** – *N*-((4-bromophenyl)(diphenylphosphaneyl)methyl)aniline S48
4. Preparatory Scale Synthesis S49

4.1. Preparatory Scale Synthesis of (*Z*)-4-(2-(diphenylphosphaneyl)vinyl)aniline (4h)....S50

4.2. Preparatory Scale Synthesis of phenethyldiphenylphosphane (6a)....S52

4.3. Preparatory Scale Synthesis of (4-methoxyphenethyl)diphenylphosphane (6b)....S54

4.4. Preparatory Scale Synthesis of (4-methylphenethyl)diphenylphosphane (6c)....S57

4.5. Preparatory Scale Synthesis of (4-fluorophenethyl)diphenylphosphane (6d)....S59

4.6. Preparatory Scale Synthesis of (4-bromophenethyl)diphenylphosphane (6e)....S62

4.7. Preparatory Scale Synthesis of diphenyl(2-(triethoxysilyl)ethyl)phosphane (6g)....S65

5. Comparing Catalytic Activity Of Different Zintl Phases....S67

6. Stoichiometric Reactions of $HPPh_2$ with Zintl Ions/ Phases....S73

7. Stoichiometric Reactions of Substrates with Zintl Ion A....S76

8. References....S76

1. Methods & Materials

1.1. Experimental Considerations

Unless stated otherwise, all manipulations were performed under an inert atmosphere using standard Schlenk-line, and glovebox techniques. Glassware was flame dried prior to use.

Dry THF, Et_2O, toluene, and pentane were obtained using Innovative Technologies anhydrous engineering solvent purification systems and subsequently degassed. DME, *o*DFB, and pyridine was dried over Na and purified by distillation. $CDCl_3$, and C_6D_6 was dried over activated 3 Å molecular sieves. All solvents were stored over activated 3 Å molecular sieves.

Elemental phosphorus (Merck), grey arsenic (Alfa Aesar), antimony (Thermo Scientific), germanium (Thermo Scientific), tin (Thermo Scientific), bismuth (Alfa Aesar), naphthalene (Fluorochem), 18-crown-6 (Fluorochem), and all organic substrates (**2a-m**, **5a-h**, and **7a**) used in this study were purchased from commercial sources and used without any further purification, imines **7b** to **7d** were prepared according to literature.[1] $[Na(18\text{-}c\text{-}6)]_2[(BBN)P_7]$,[2] $[Na(DME)_x]_3[P_7]$,[3] $[K(DME)_X]_3[P_7]$,[4] $[K(DME)_X]_3[As_7]$,[4] K_3P_7,[5] K_3Sb_7,[6] K_4Ge_9,[7] K_4Sn_9,[7] and K_5Bi_4[8] were synthesized according to previously reported synthetic procedures.

1.2. Analytical Considerations

NMR Spectroscopy. ^{1}H, $^{13}C\{^{1}H\}$, ^{19}F, ^{29}Si DEPT90, $^{31}P\{^{1}H\}$ NMR spectra were recorded on a Bruker AVIII 400 spectrometer (operating frequencies: 399.78 MHz, 100.53 MHz, 376.17 MHz, 79.48 MHz and 161.83 MHz for ^{1}H, ^{13}C, ^{19}F, ^{29}Si and ^{31}P, respectively). ^{1}H and $^{13}C\{^{1}H\}$ NMR chemical shifts were internally referenced to the residual solvent resonances ($CDCl_3$ (chloroform-d): ^{1}H δ = 7.26 ppm, $^{13}C\{^{1}H\}$ δ = 77.16 ppm, C_6D_6 (benzene-d_6): ^{1}H δ = 7.16 ppm, $^{13}C\{^{1}H\}$ δ = 128.02 ppm). ^{19}F, ^{29}Si, $^{31}P\{^{1}H\}$ chemical shifts were externally referenced to $CFCl_3$, Me_4Si, H_3PO_4, respectively. Hydrophosphination reactions were prepared under an inert atmosphere in 5 mm J Young NMR tubes, isolated products were prepared on the bench and spectra recorded in 5 mm NMR tubes. Data was analysed using MestReNova V14.0.0 software.

Elemental Analysis. Elemental analysis was carried out by Mr. Martin Jennings and Mrs. Anne Davies at the Microanalytical Service, School of Chemistry, the University of Manchester using a Flash 2000 elemental analyser. The elemental analysis carbon value found for the scaled synthesis of compound **4h** was larger than expected, despite multiple attempts at obtaining better data. Best data is reported, and NMR spectroscopy and mass spectrometry data are consistent with product formulation. It is noteworthy, that in a study carried out by Melen and co-workers evaluating elemental analysis found that random error can variably lead to differences outside of 0.4%.[9]

Mass spectrometry. Mass spectrometry samples were measured through the Mass Spectrometry Service, School of Chemistry, the University of Manchester using an electrospray ionization or atmospheric pressure chemical ionization equipped Thermo Orbitrap Executive Plus Extended Mass Range mass spectrometer. Despite multiple efforts, we were unable to obtain mass spectra from the products of imine hydrophosphination (**8a-d**).

1.3. Hydrophosphination Scope Procedure

In a nitrogen glovebox, a J Young NMR tube was loaded with catalyst **A** (1 mg, 1 mol%) and 18-crown-6 (2.4 mg, 3 mol%). The unfunctionalized organic substrate (0.3 mmol) was dissolved in pyridine (0.6 mL) and transferred to the J Young NMR tube followed by the addition of diphenylphosphine (52.2 µL, 0.3 mmol) *via* microsyringe. The reaction progress was monitored by $^{31}P\{^{1}H\}$ NMR spectroscopy, completion was determined by the absence of diphenylphosphine ($^{31}P\{^{1}H\}$ NMR (162 MHz, 298 K, pyridine): δ = 40.24 ppm).

2. Screening Conditions

2.1. Control Solvent Screening

The relevant substrate (0.3 mmol) was dissolved in pyridine (0.6 mL) and transferred to the J Young NMR tube followed by the addition of diphenylphosphine (52.2 µL, 0.3 mmol) *via* microsyringe. The reaction progress was monitored by $^{31}P\{^{1}H\}$ NMR spectroscopy, completion was determined by the absence of diphenylphosphine ($^{31}P\{^{1}H\}$ NMR (162 MHz, 298 K, pyridine): δ = 40.24 ppm).

Table S1. Control reactions for the hydrophosphination of phenylacetylene (**2a**), styrene (**5a**) and *N*-benzylideneaniline (**7a**).

Entry	Substrate	Product	Solvent	*t* (h)	T (°C)	% Conv. [a]
1			C_6D_6	24	25	0
2	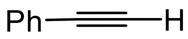	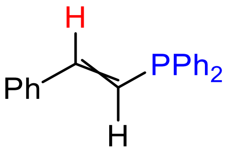	THF	24	25	0
3	**2a**		*o*DFB	24	25	0
4		**3a/4a**	pyridine	24	25	0
5			C_6D_6	24	25	1
6	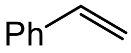	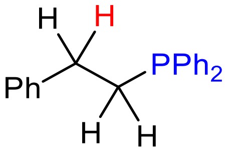	THF	24	25	1
7	**5a**		*o*DFB	24	25	0
8		**6a**	pyridine	24	25	3
9			C_6D_6	24	25	20
10	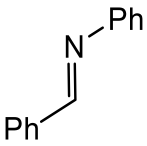	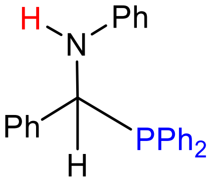	THF	24	25	18
11			*o*DFB	24	25	74
12	**7a**	**8a**	pyridine	24	25	0

[a] Conversion determined by $^{31}P\{^{1}H\}$ NMR spectroscopy. Note for the hydrophosphination of phenylacetylene (**2a**) both *E*- (**3a**) and *Z*-isomers (**4a**) are observed products.

2.2. Solvent screening for the hydrophosphination of phenylacetylene with $[Na(18\text{-}c\text{-}6)]_2[(BBN)P_7]$ (1).

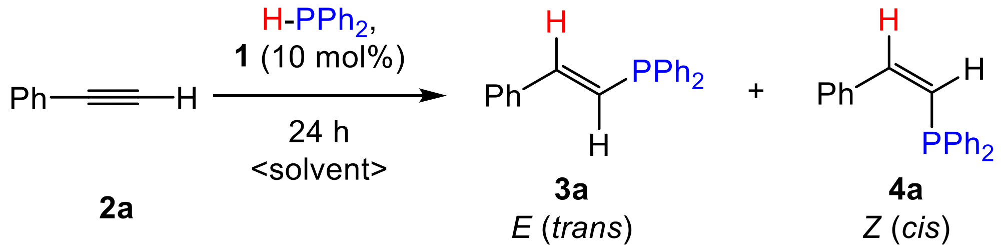

$[Na(18\text{-}c\text{-}6)]_2[(BBN)P_7]$ (**1**, 10.2 mg, 0.01 mmol) was added to a J Young NMR tube followed by the addition of solvent (0.6 mL, solvents = C_6D_6, THF, *o*DFB, pyridine). Phenylacetylene (**2a**, 11 µL, 0.1 mmol) and diphenylphosphine (17.4 µL, 0.1 mmol) were subsequently transferred. The reaction was monitored after 24 hours at either room temperature (RT) or 50 °C by $^{31}P\{^1H\}$ NMR spectroscopy, completion was determined by the absence of diphenylphosphine ($^{31}P\{^1H\}$ NMR (162 MHz, 298 K, pyridine): δ = 40.24 ppm).

Note: the resonance at –15 ppm is the coupled phosphorus compound $Ph_2P–PPh_2$.[10]

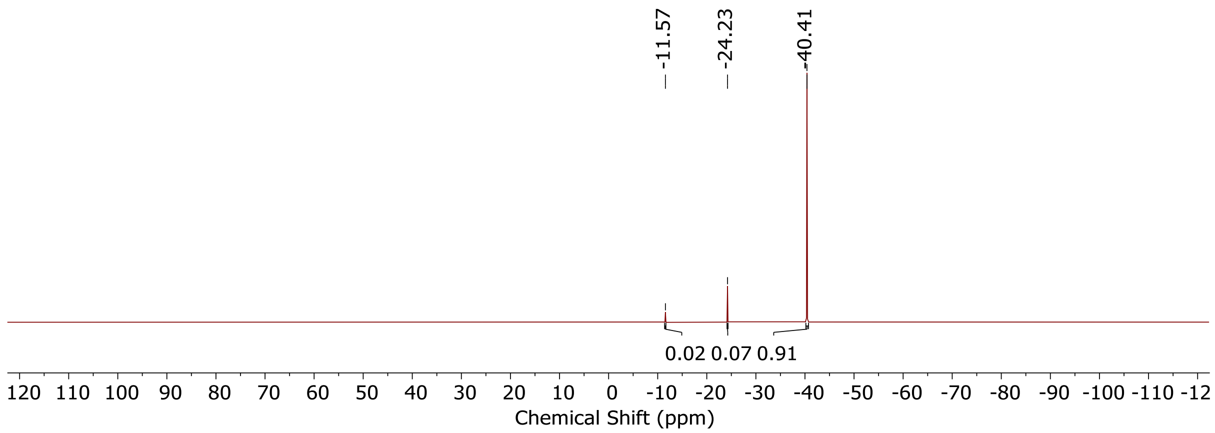

Figure S1. $^{31}P\{^1H\}$ NMR spectrum (C_6D_6) of crude **3a**/**4a** after 24h at RT.

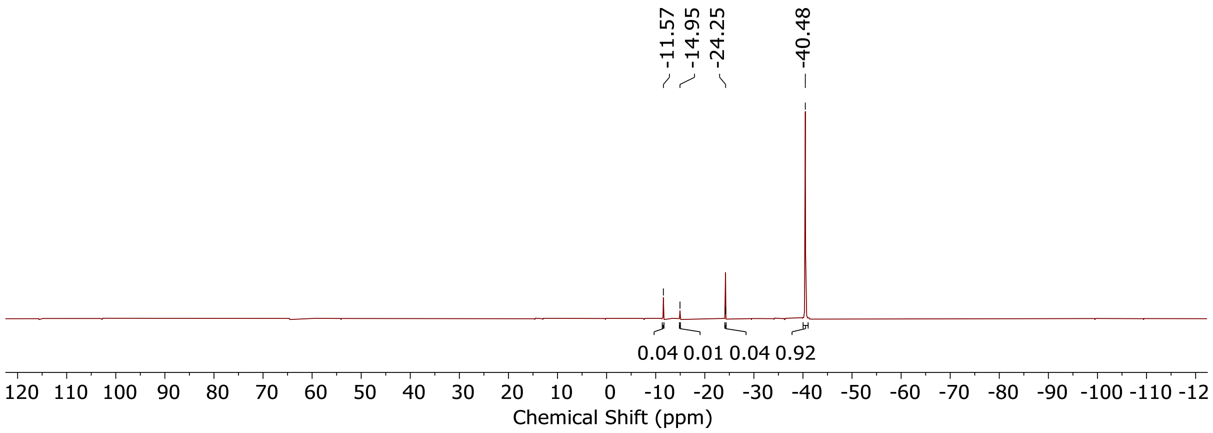

Figure S2. $^{31}P\{^{1}H\}$ NMR spectrum (C_6D_6) of crude **3a**/**4a** after 24h at 50 °C.

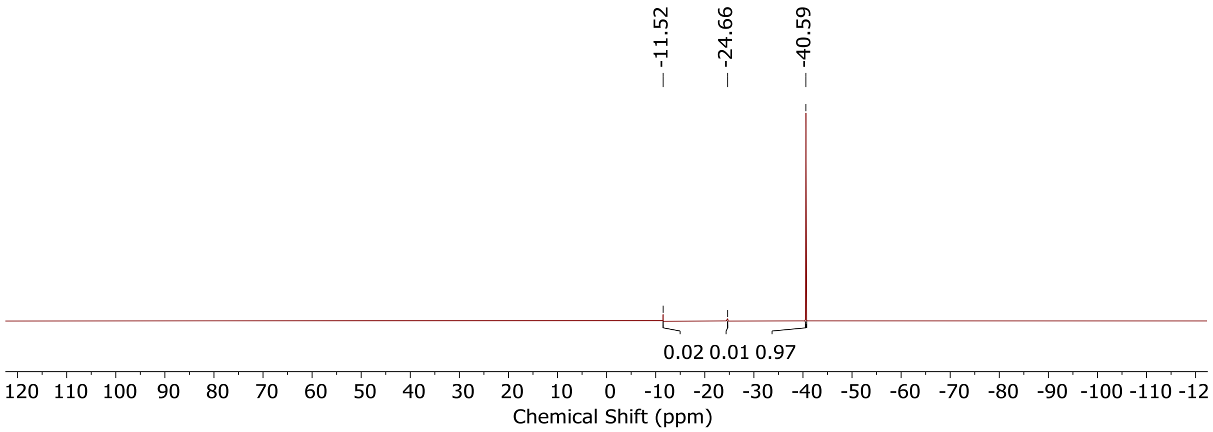

Figure S3. $^{31}P\{^{1}H\}$ NMR spectrum (THF) of crude **3a**/**4a** after 24h at RT.

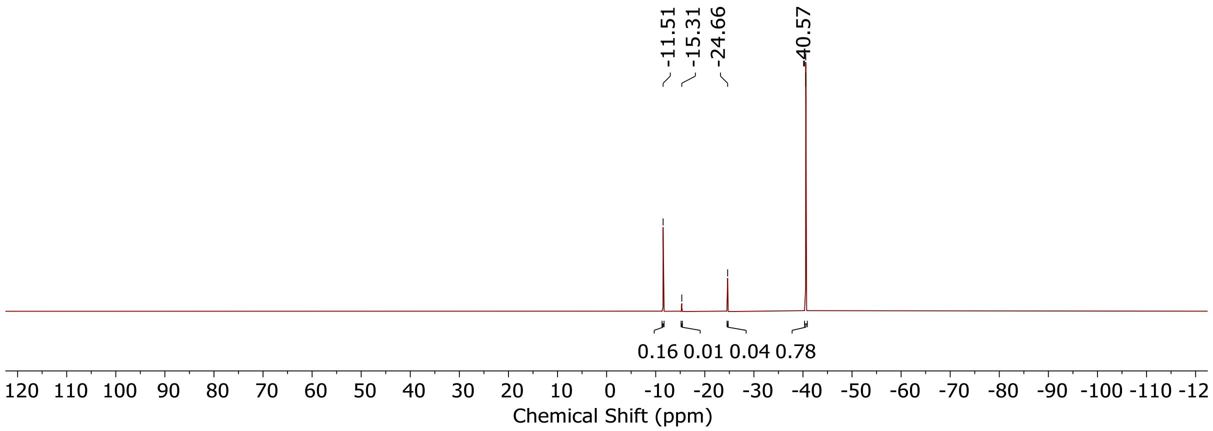

Figure S4. $^{31}P\{^{1}H\}$ NMR spectrum (THF) of crude **3a**/**4a** after 24h at 50 °C.

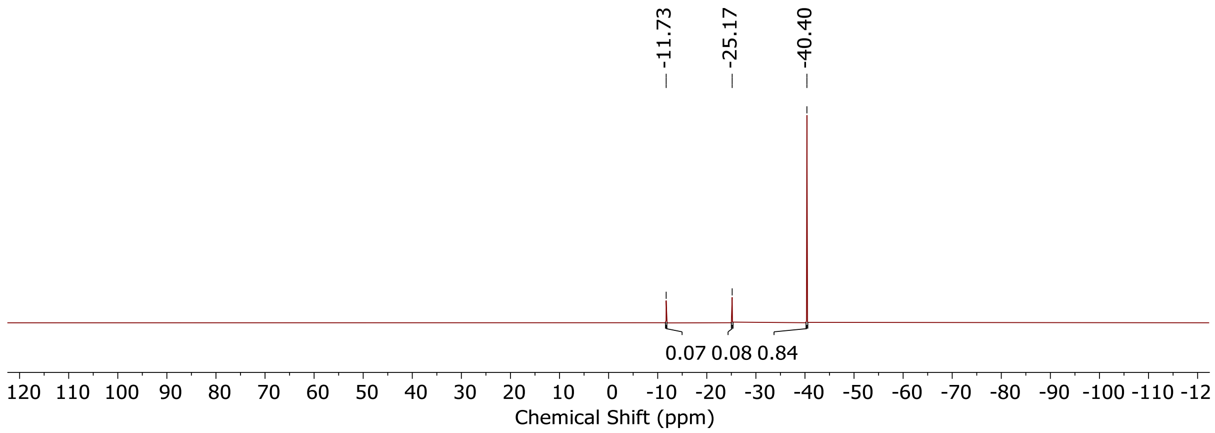

Figure S5. $^{31}P\{^1H\}$ NMR spectrum (*o*DFB) of crude **3a**/**4a** after 24h at RT.

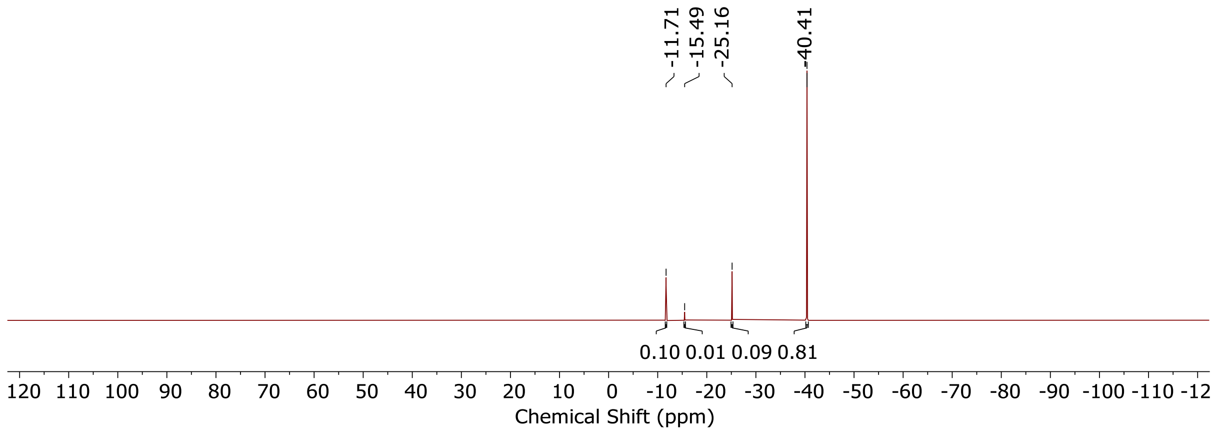

Figure S6. $^{31}P\{^1H\}$ NMR spectrum (*o*DFB) of crude **3a**/**4a** after 24h at 50 °C.

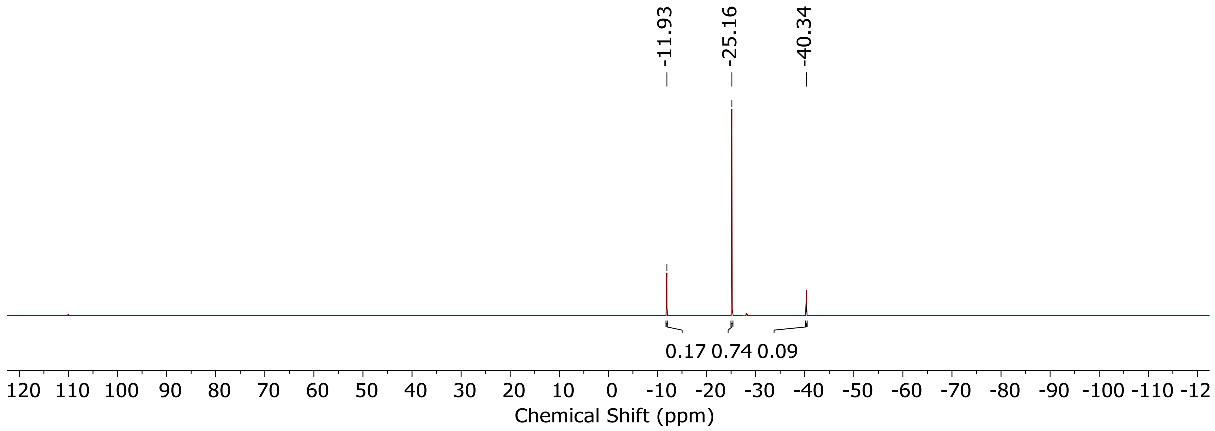

Figure S7. $^{31}P\{^1H\}$ NMR spectrum (pyridine) of crude **3a**/**4a** after 24h at RT.

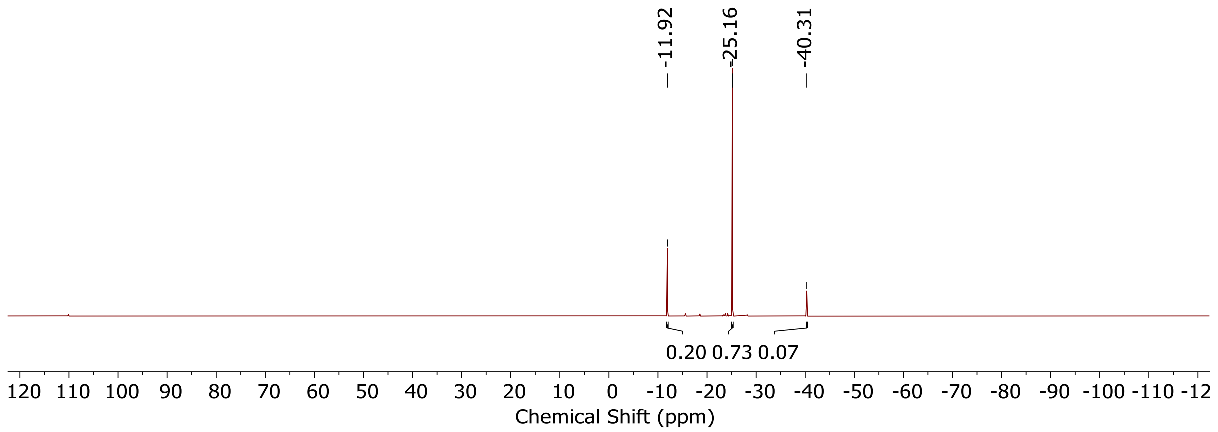

Figure S8. $^{31}P\{^1H\}$ NMR spectrum (pyridine) of crude **3a**/**4a** after 24h at 50 °C.

2.3. Screening initiators for the hydrophosphination of phenylacetylene

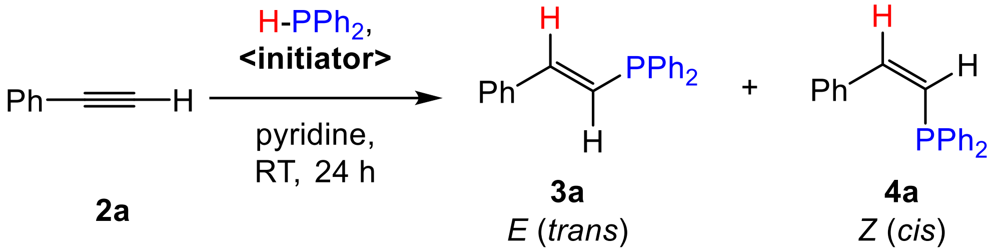

Initiator (5 mol%, 0.005 mmol $(HBBN)_2$ = 1.2 mg; 10 mol%, 0.01 mmol: $(Me_3Si)_3P_7$ = 4.4 mg, $[Na(DME)_x]_3[P_7]$ = 3.0 mg, NaOTf = 1.7 mg; 30 mol%, 0.03 mmol, 18-crown-6 = 7.9 mg) was added to a J Young NMR tube followed by the addition of pyridine (0.6 mL). Phenylacetylene (**2a**, 11 µL, 0.1 mmol) and diphenylphosphine (17.4 µL, 0.1 mmol) were subsequently transferred. The reaction was monitored after 24 hours at room temperature (RT) by $^{31}P\{^1H\}$ NMR spectroscopy, completion was determined by the absence of diphenylphosphine ($^{31}P\{^1H\}$ NMR (162 MHz, 298 K, pyridine): δ = 40.24 ppm).

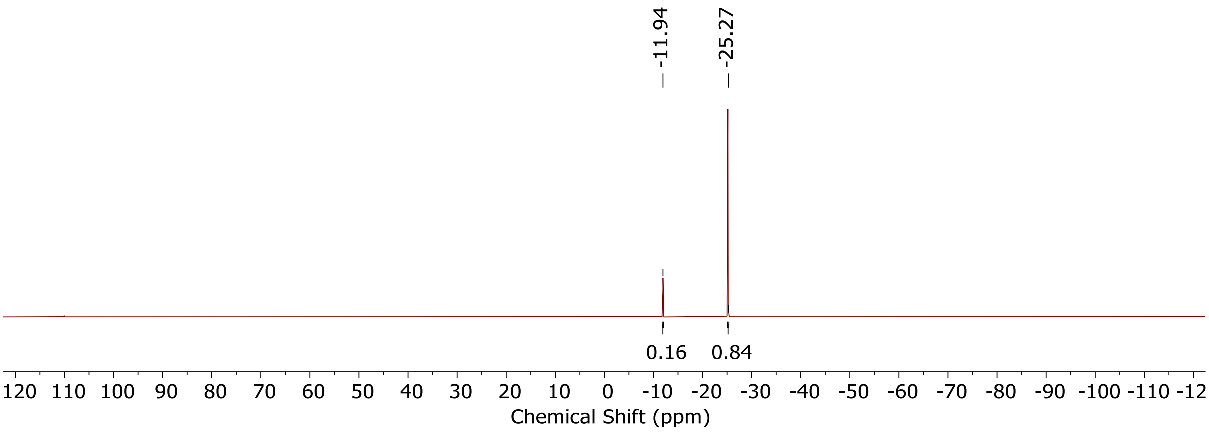

Figure S9. $^{31}P\{^{1}H\}$ NMR spectrum (pyridine) of crude **3a**/**4a** after 24h at RT with 10 mol% $[Na(DME)_x]_3[P]_7$.

2.4. Determining DME content of $[Na(DME)_x]_3[P_7]$ (A)

Elemental analysis of **A** to determine the C and H content and allowed for the determination of DME content.

**Found**	**C**	**H**
Run 1	2.71	0.38
Run 2	2.75	0.38
Run 3	2.77	0.37

We found consistent results across all runs, from there we determined x = 0.05 to be the most accurate balance of C and H values providing a molecular weight of 299.304 g/ mol for **A**.

**x**	**3x**	**C**	**H**
0.035	0.115	1.87	0.39
0.050	0.150	2.41	0.51
0.055	0.175	2.79	0.58

We also determined the DME content of $[K(DME)_x]_3[P_7]$ (**B**) and $[K(DME)_x]_3[As_7]$ (**C**). We found x = 0.030 and 0.060/ K cation and molecular weights of 342.222 and 657.968 g/mol for **B** and **C**, respectively.

Found	B		C	
	C	H	C	H
Run 1	2.18	0.26	1.54	0.22
Run 2	1.93	0.24	1.68	0.23
Run 3	1.72	0.25	1.47	0.21

B				C			
x	3x	C	H	x	3x	C	H
0.027	0.080	1.13	0.24	0.050	0.150	1.10	0.23
0.030	0.090	1.26	0.27	0.055	0.165	1.21	0.25
0.033	0.100	1.40	0.29	0.060	0.180	1.31	0.28

2.5. Solvent Screening for the hydrophosphination of phenylacetylene with A

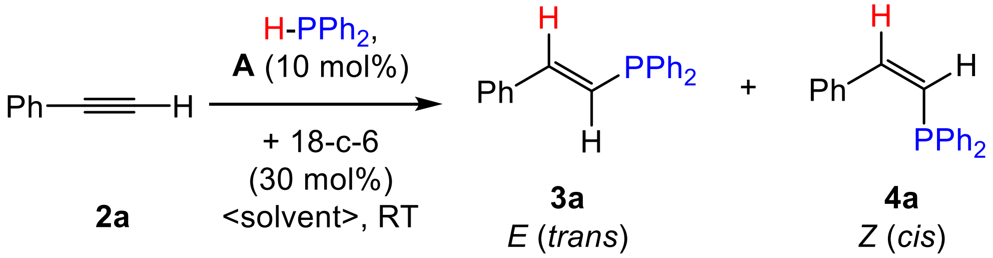

Table S2. Solvent screening for the hydrophosphination of phenylacetylene (**2a**) with **A**.

Entry	Substrate	Product	Solvent	*t* (h)	% Conv.[a] (3a: 4a)
1			C_6D_6	0.25	0
2			C_6D_6	24	0
3			THF	0.25	0
4	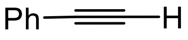 **2a**	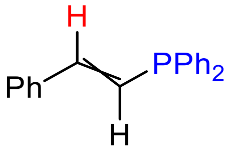 **3a/4a**	THF	24	56 (15: 41)
5			*o*DFB	0.25	0
6			*o*DFB	24	9 (1: 8)
7			pyridine	0.25	>99 (23: 77)

[a] Conversion determined by $^{31}P\{^1H\}$ NMR spectroscopy. Note for the hydrophosphination of phenylacetylene (**2a**) both *E*- (**3a**) and *Z*-isomers (**4a**) are observed products.

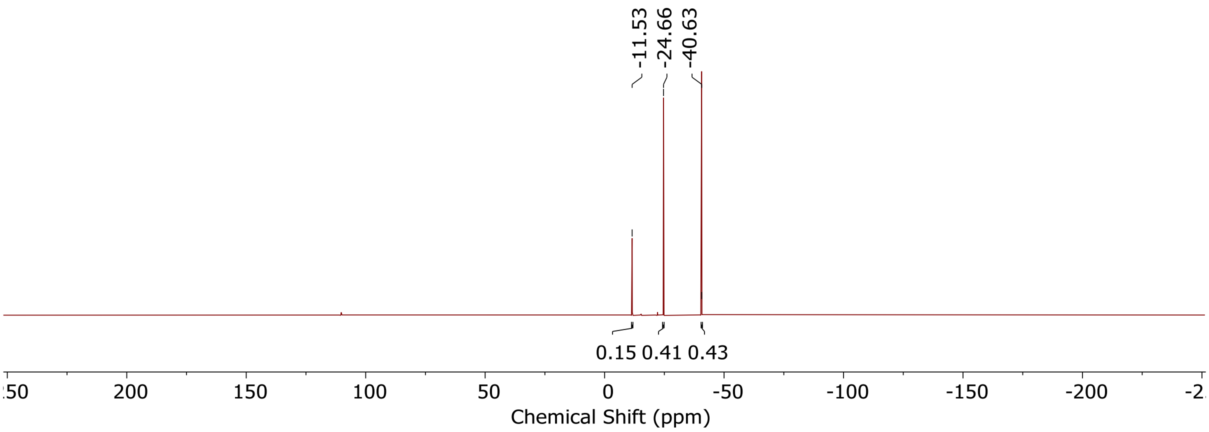

Figure S10. $^{31}P\{^{1}H\}$ NMR spectrum (THF) of crude **3a**/**4a** after 24h at RT.

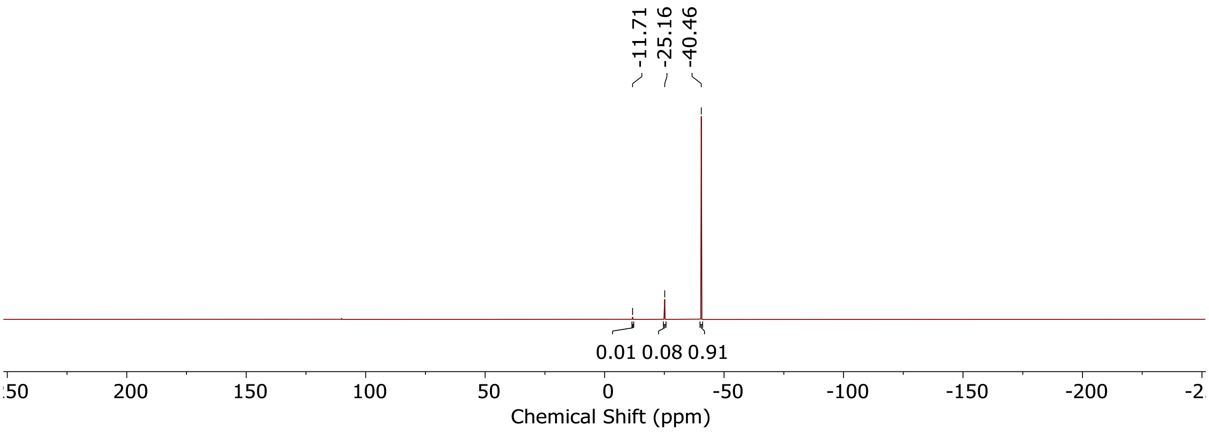

Figure S11. $^{31}P\{^{1}H\}$ NMR spectrum (*o*DFB) of crude **3a**/**4a** after 24h at RT.

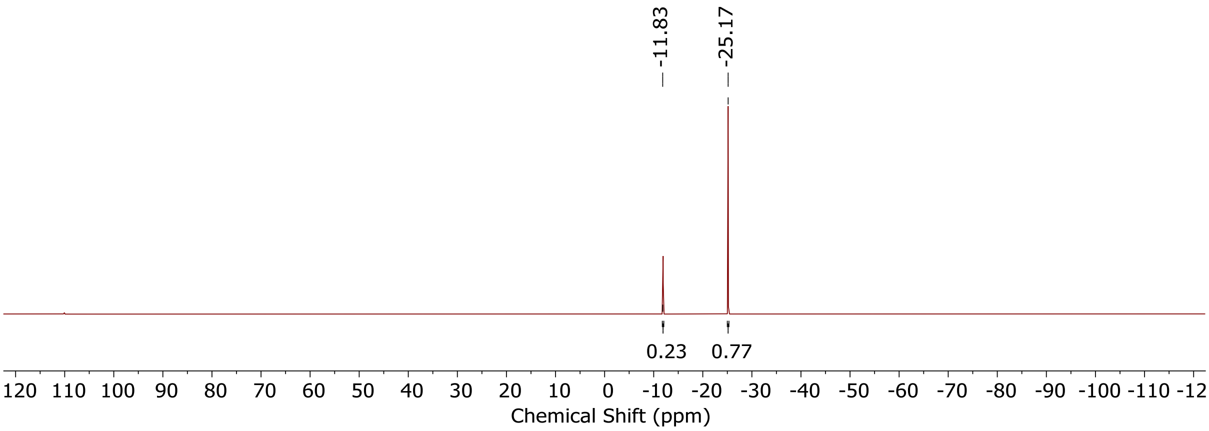

Figure S12. $^{31}P\{^{1}H\}$ NMR spectrum (pyridine) of crude **3a**/**4a** after 15 minutes at RT.

2.6. Cation sequestering agent selection

A (2 mg, 0.0067 mmol, 3.33 mol%) and cation sequestering agent (10 mol%, 0.02 mmol, 15-c-5 = 4.0 µL, 2.2.2-cryptand = 7.5 mg) was added to a J Young NMR tube followed by the addition of pyridine (0.6 mL). Phenylacetylene (**2a**, 22 µL, 0.2 mmol) and diphenylphosphine (34.8 µL, 0.2 mmol) were subsequently transferred. The

reaction was monitored at room temperature (RT) by $^{31}P\{^1H\}$ NMR spectroscopy, completion was determined by the absence of diphenylphosphine ($^{31}P\{^1H\}$ NMR (162 MHz, 298 K, pyridine): δ = 40.24 ppm).

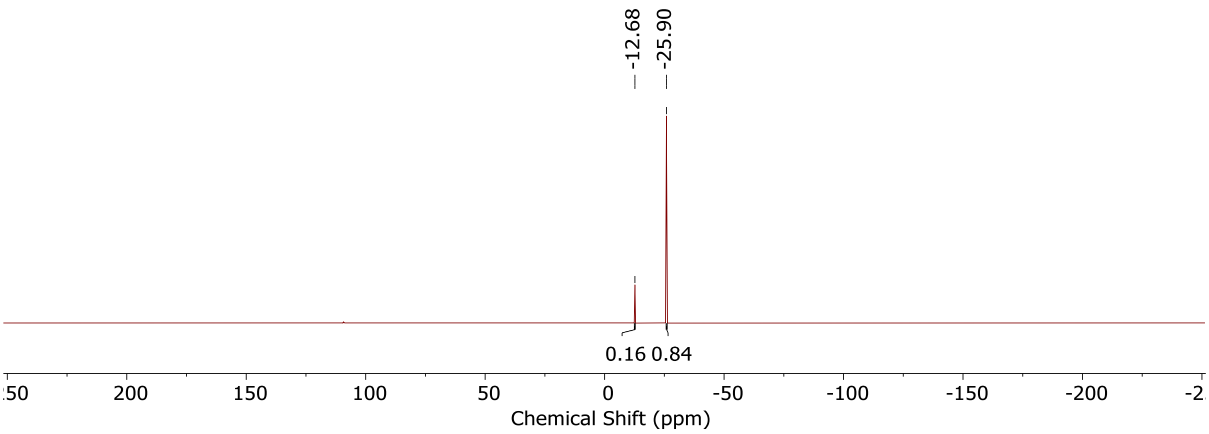

Figure S13. $^{31}P\{^1H\}$ NMR spectrum (pyridine) of crude **3a**/**4a** after 15 minutes at RT with 10 mol% 15-crown-5 as the cation sequestering agent.

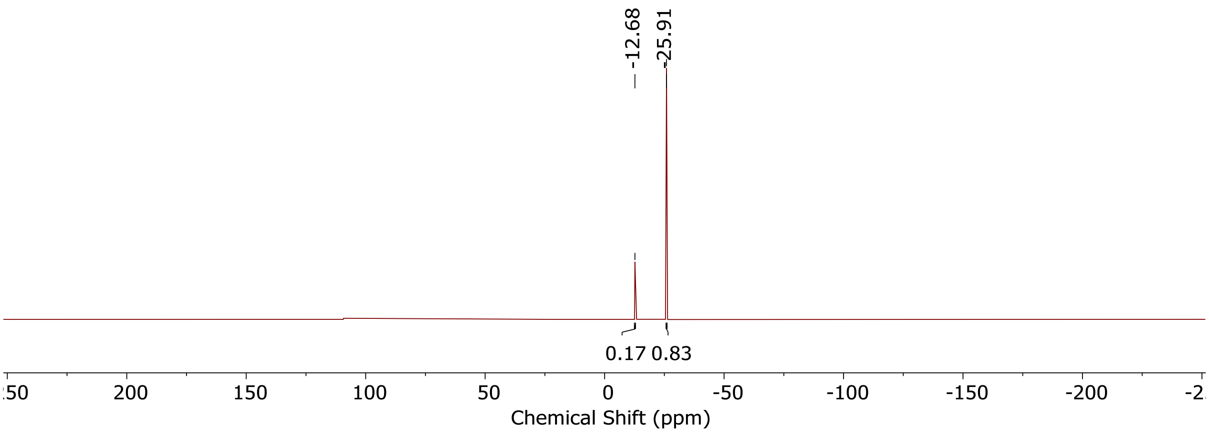

Figure S14. $^{31}P\{^1H\}$ NMR spectrum (pyridine) of crude **3a**/**4a** after 15 minutes at RT with 10 mol% 2.2.2-cryptand as the cation sequestering agent.

2.7. Screening 18-crown-6 loadings

We investigated if there was any effect on the loading of 18-crown-6 (18-c-6). We found that at lower loadings the reaction time was slightly impacted. When no 18-crown-6 was added there was a significant impact on time, consistent with the role of the 18-crown-6 to aid solubility of **A**. At higher loadings, the effect was minimal and thus a loading of be three equivalents of 18-c-6 relative to the loading of **A** was employed.

A (3 mg, 0.01 mmol, 10 mol%) and 18-crown-6 (50 mol %, 0.05 mmol, 13.2 mg; 20 mol%, 0.02 mmol, 5.2 mg; 10 mol%, 0.01 mmol, 2.6 mg; 5 mol%, 0.05 mmol, 1.3 mg) was added to a J Young NMR tube followed by the addition of pyridine (0.6 mL). Phenylacetylene (**2a**, 11μL, 0.1 mmol) and diphenylphosphine (17.4 μL, 0.1 mmol) were subsequently transferred. The reaction was monitored at room temperature (RT) by $^{31}P\{^{1}H\}$ NMR spectroscopy, completion was determined by the absence of diphenylphosphine ($^{31}P\{^{1}H\}$ NMR (162 MHz, 298 K, pyridine): δ = 40.24 ppm).

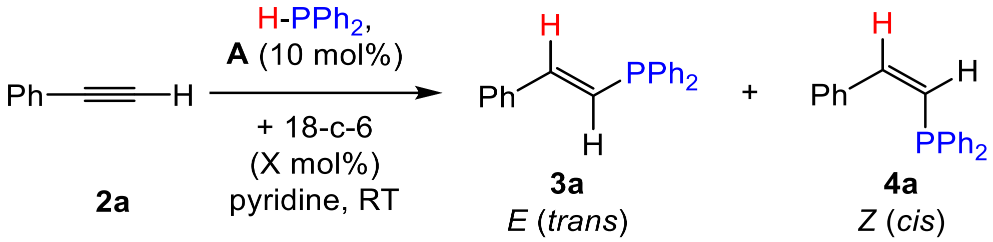

Table S3. Screening 18-c-6 loading for the hydrophosphination of phenylacetylene (**2a**) with **A**.

Entry	18-c-6 Loading (mol%)	*t* (h)	% Conv.[a] (3a: 4a)
1	50	0.25	>99 (19: 81)
2	20	0.25	>99 (19: 81)
3	10	0.50	>99 (23: 77)
4	5	0.50	>99 (20: 80)

[a] Conversion determined by $^{31}P\{^{1}H\}$ NMR spectroscopy. Note for the hydrophosphination of phenylacetylene (**2a**) both *E*- (**3a**) and *Z*-isomers (**4a**) are observed products.

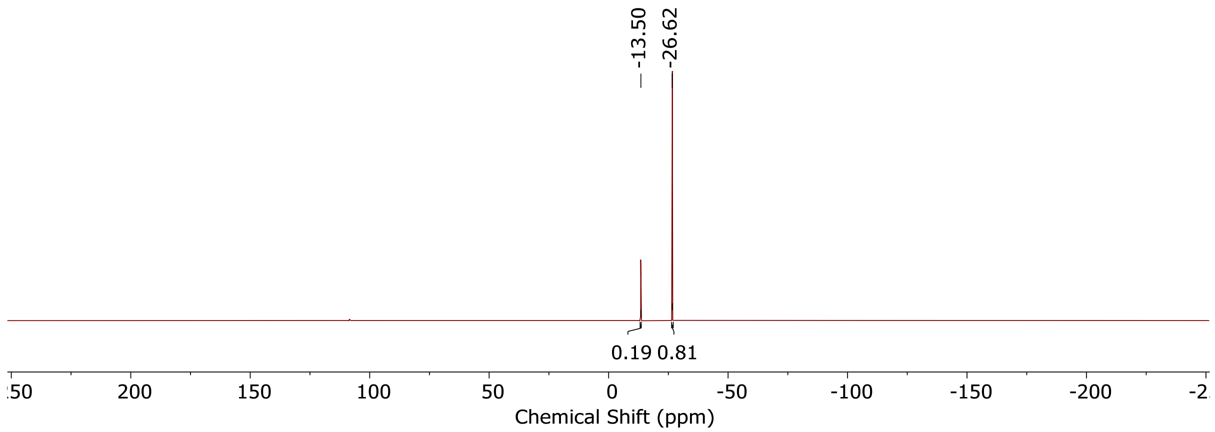

Figure S15. $^{31}P\{^{1}H\}$ NMR spectrum (pyridine) of crude **3a**/**4a** after 15 minutes at RT with 50 mol% 18-c-6.

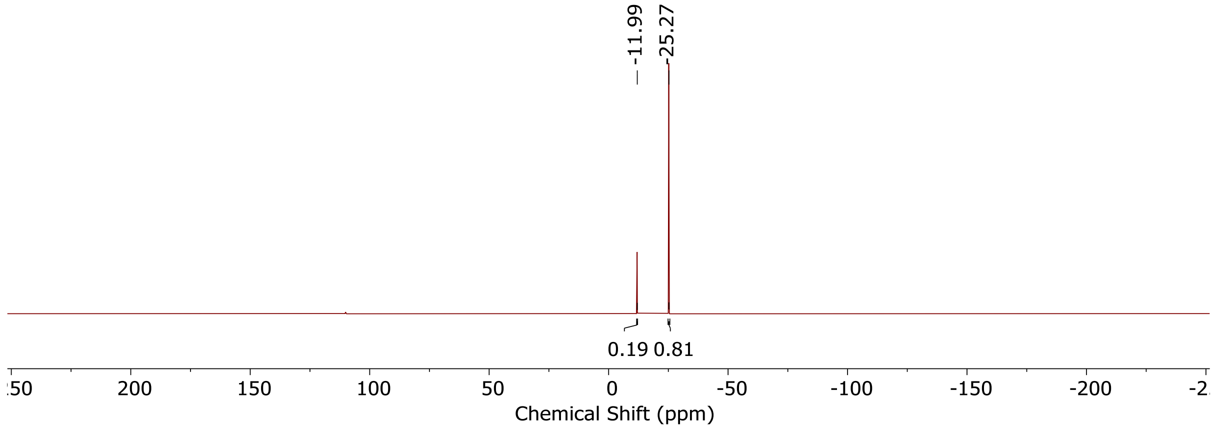

Figure S16. $^{31}P\{^{1}H\}$ NMR spectrum (pyridine) of crude **3a**/**4a** after 15 minutes at RT with 20 mol% 18-c-6.

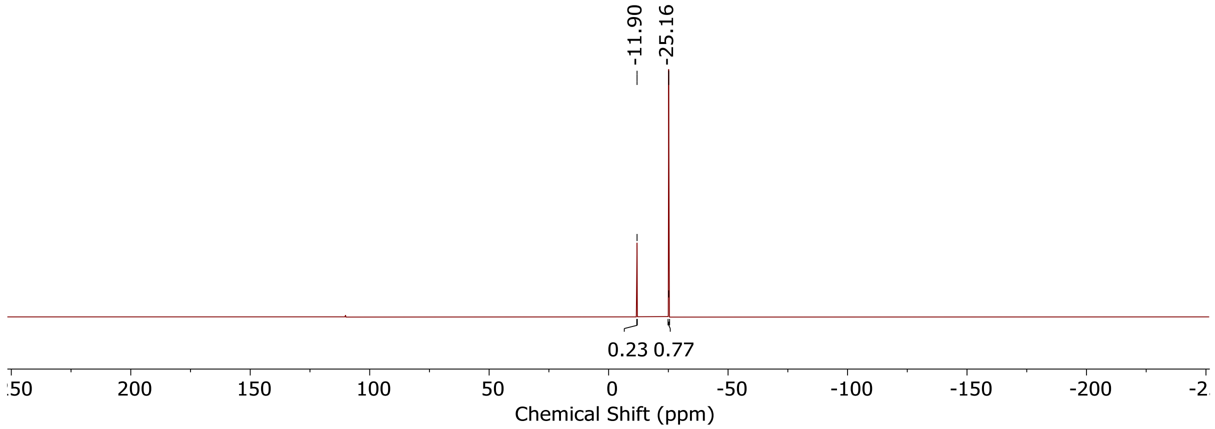

Figure S17. $^{31}P\{^{1}H\}$ NMR spectrum (pyridine) of crude **3a**/**4a** after 30 minutes at RT with 10 mol% 18-c-6.

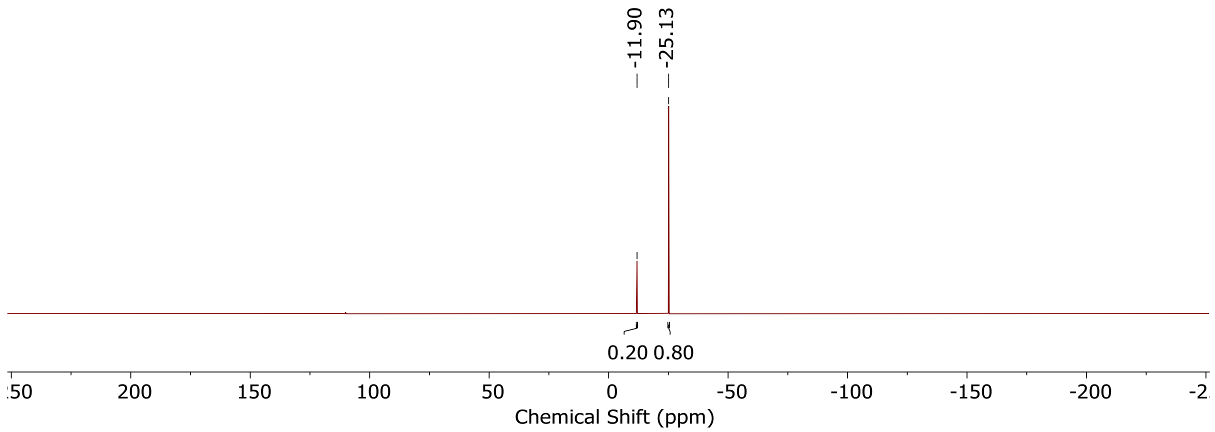

Figure S18. $^{31}P\{^1H\}$ NMR spectrum (pyridine) of crude **3a**/**4a** after 30 minutes at RT with 5 mol% 18-c-6.

2.8. Screening Initiator Loading

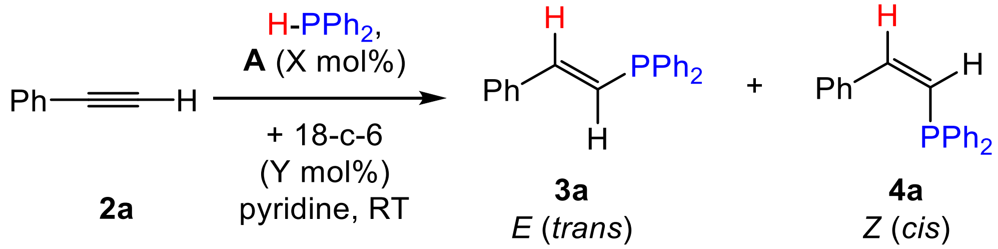

Table S4. Screening initiator **A** loading for the hydrophosphination of phenylacetylene (**2a**).

Entry	A Loading (mol%)	18-c-6 Loading (mol%)	*t* (h)	% Conv.[a] (3a: 4a)	Reaction Scale (mmol)
1	5	15	0.25	>99 (16: 82)	0.1
2	3.33	10	0.25	>99 (15: 85)	0.2
3	2.5	7.5	0.25	>99 (17: 83)	0.2
4	1	3	0.25	>99 (14: 86)	0.3

5	0.5	1.5	12	87 (11: 76)	0.3

[a] Conversion determined by $^{31}P\{^1H\}$ NMR spectroscopy. Note for the hydrophosphination of phenylacetylene (**2a**) both *E*- (**3a**) and *Z*-isomers (**4a**) are observed products.

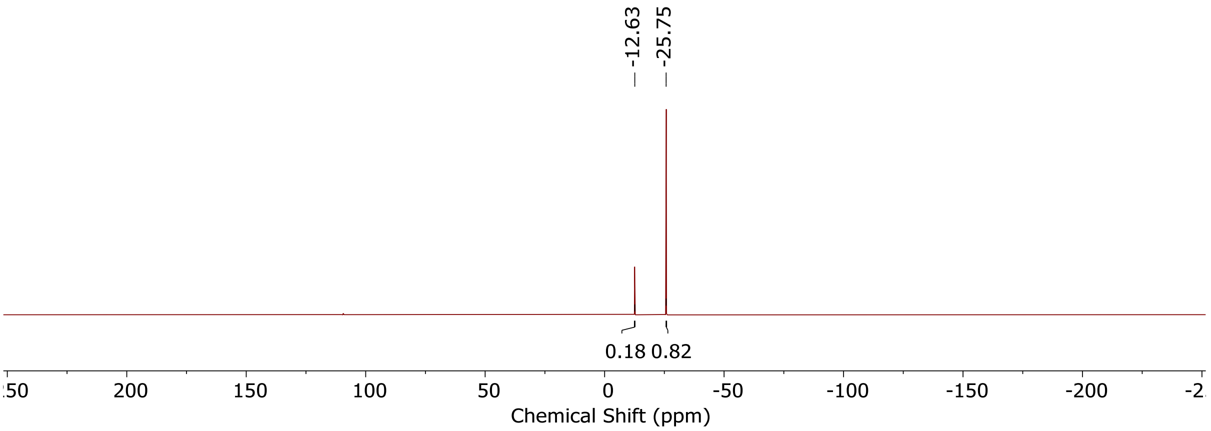

Figure S19. $^{31}P\{^1H\}$ NMR spectrum (pyridine) of crude **3a**/**4a** after 15 minutes at RT with 5 mol% **A** + 15 mol% 18-c-6.

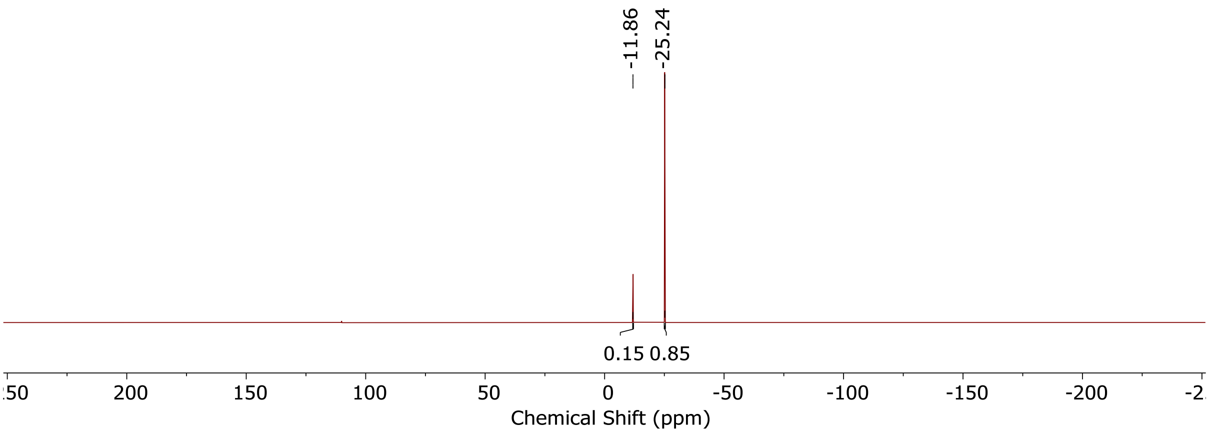

Figure S20. $^{31}P\{^1H\}$ NMR spectrum (pyridine) of crude **3a**/**4a** after 15 minutes at RT with 3.33 mol% **A** + 10 mol% 18-c-6.

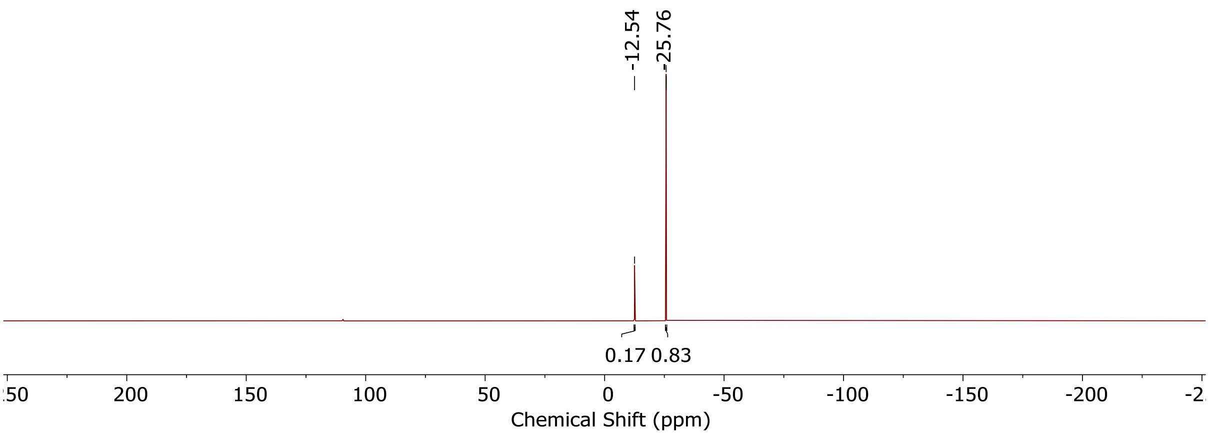

Figure S21. $^{31}P\{^1H\}$ NMR spectrum (pyridine) of crude **3a**/**4a** after 15 minutes at RT with 2.5 mol% **A** + 7.5 mol% 18-c-6.

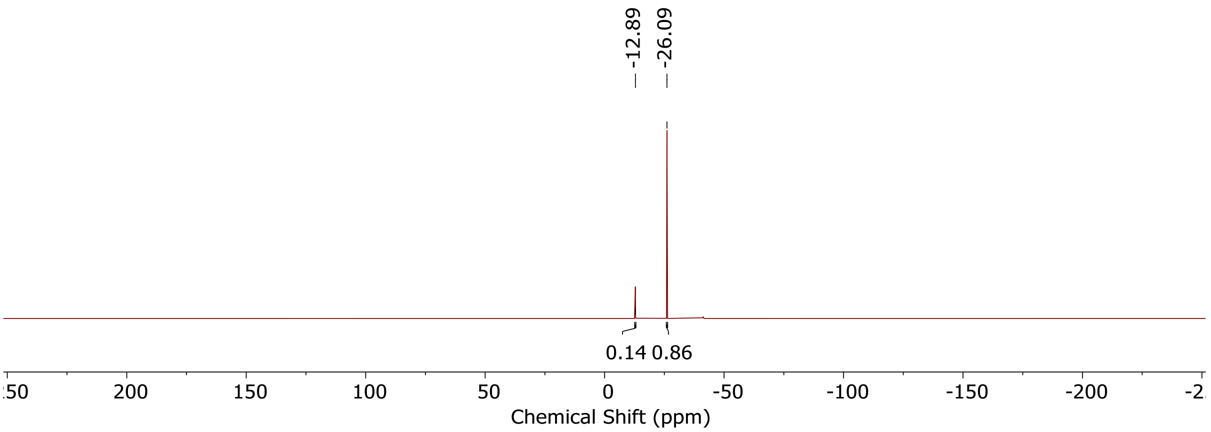

Figure S22. $^{31}P\{^{1}H\}$ NMR spectrum (pyridine) of crude **3a**/**4a** after 15 minutes at RT with 1 mol% **A** + 3 mol% 18-c-6.

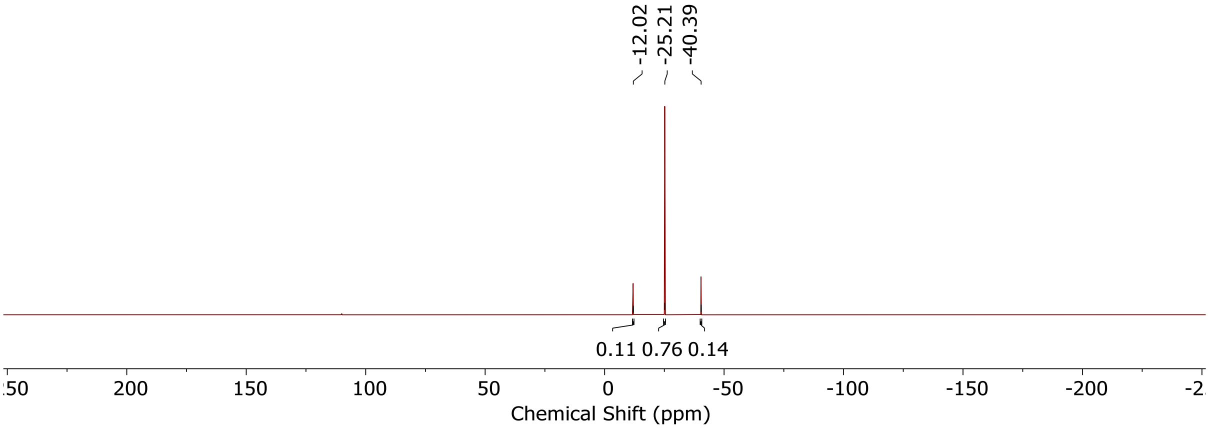

Figure S23. $^{31}P\{^{1}H\}$ NMR spectrum (pyridine) of crude **3a**/**4a** after 12h at RT with 0.5 mol% **A** + 1.5 mol% 18-c-6.

3. Characterisation Data

For hydrophosphination of alkynes, alkenes and imines the general procedure described in section 1.3 was used. Conversions were determined by $^{31}P\{^{1}H\}$ NMR spectroscopy. Data obtained was in line with previous literature for hydrophosphinated alkynes,[11, 12] alkenes,[11, 13, 14] and imines.[14, 15]

3.1. Products of Alkyne Hydrophosphination

Note: Where possible, ^{1}H NMR resonance of both isomers have been identified, otherwise only major isomer is given. For the minor isomer, integration is given as a percentage of the major isomer in accordance with its conversion where possible.

3.1.1. **3a**/**4a** – (*E*/*Z*)-diphenyl(styryl)phosphane

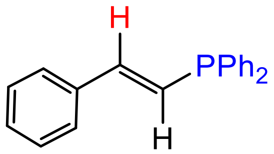

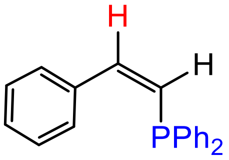

3a (*E*)-diphenyl(styryl)phosphane   **4a** (*Z*)-diphenyl(styryl)phosphane

Conversion: >99%; **3a** – 15%, **4a** – 85%.

^{1}H NMR (400 MHz, 298 K, pyridine): δ = 6.23 (dd, $^{3}J_{HH}$ = 12.81 Hz, $^{3}J_{HP}$ = 2.87 Hz, 1H, C*H*=CHPPh$_2$, **4a**), 7.31–7.38 (m, 13H, Ar–*H*, **3a**/**4a**), 7.46 (dd, $^{2}J_{HP}$ = 23.76 Hz, $^{3}J_{HH}$ = 12.81 Hz, 1H, CH=C*H*PPh$_2$, **4a**), 7.74 (d, $^{3}J_{HH}$ = 8.25 Hz, 2H, Ar–*H*, **3a**/**4a**). *Note – **3a** CH=CH resonances could not be identified, obscured by solvent resonances.*

^{31}P{^{1}H} NMR (162 MHz, 298 K, pyridine): δ = –11.94 (s, **3a**), –25.16 (s, **4a**).

Mass spectrometry (APCI): $C_{20}H_{17}P$+H ([M+H]$^{+}$); Calcd. = 289.1141, Found = 289.1132.

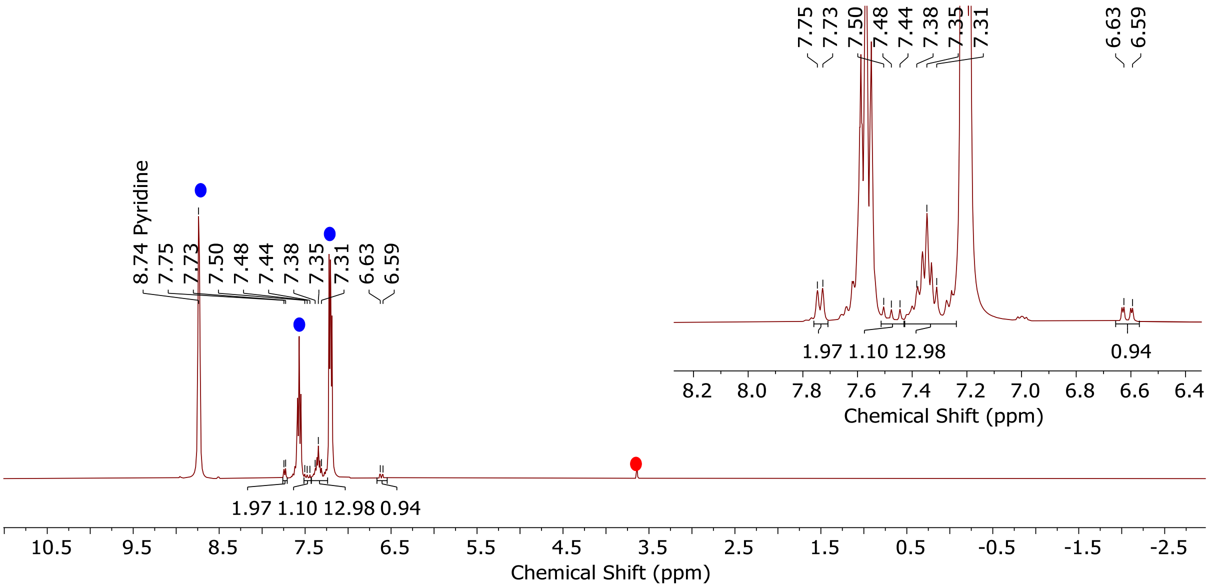

Figure S24. ^{1}H NMR spectrum (pyridine) of crude **3a**/**4a**. ● = pyridine, ● = 18-c-6.

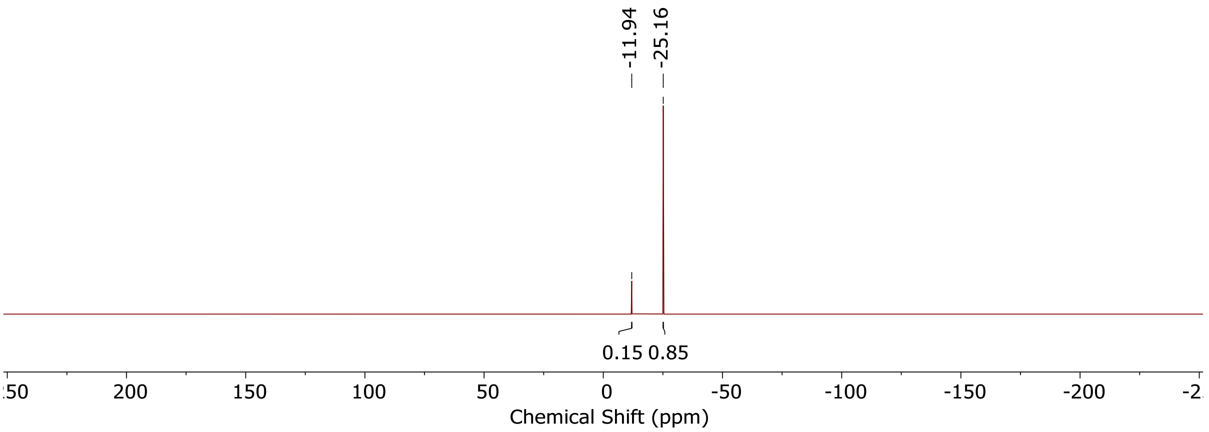

Figure S25. ^{31}P{^{1}H} NMR spectrum (pyridine) of crude **3a**/**4a**.

3.1.2. **3b**/**4b** – (*E*/*Z*)-(4-methoxystyryl)diphenylphosphane

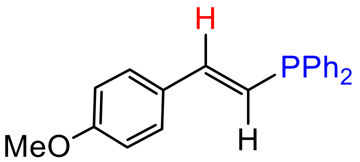

3b

(*E*)-(4-methoxystyryl)diphenylphosphane

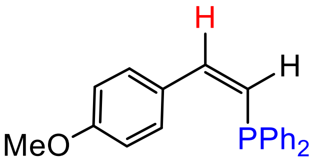

4b

(*Z*)-(4-methoxystyryl)diphenylphosphane

Conversion: >99%; **3b** – 2%, **4b** – 98%.

^{1}H NMR (400 MHz, 298 K, pyridine): δ = 3.62 (s, 3H, OC*H*$_3$, **4b**), 6.49 (dd, $^{3}J_{HH}$ = 12.33 Hz, $^{3}J_{HP}$ = 2.87 Hz, 1H, C*H*=CHPPh$_2$, **4b**), 6.93 (d, $^{3}J_{HH}$ = 8.62 Hz, 2H, Ar–*H*, **3b**/**4b**), 7.34–7.40 (m, 6H, Ar–*H*, **3b**/**4b**), 7.46 (dd, $^{2}J_{HP}$ = 24.33 Hz, $^{3}J_{HH}$ = 12.33 Hz, 1H, CH=C*H*PPh$_2$, **4b**), 7.63 (m, 4H, Ar–*H*, **3b**/**4b**), 7.73 (d, $^{3}J_{HH}$ = 8.37 Hz, 2H, Ar–*H*, **3b**/**4b**). *Note – **3b** CH=CH resonances could not be identified, too low conversion.*

^{31}P{^{1}H} NMR (162 MHz, 298 K, pyridine): δ = –11.88 (s, **3b**), –24.70 (s, **4b**).

Mass spectrometry (APCI): $C_{21}H_{19}OP$+H ([M+H]$^+$); Calcd. = 319.1246, Found = 319.1240.

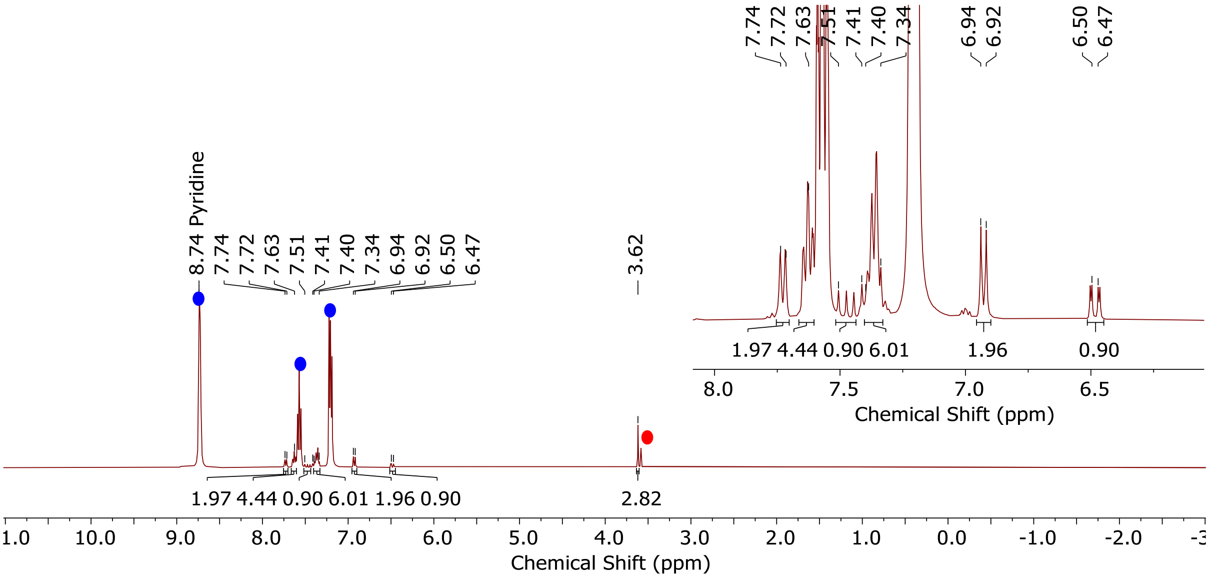

Figure S26. ^{1}H NMR spectrum (pyridine) of crude **3b/4b**. ● = pyridine, ● = 18-c-6.

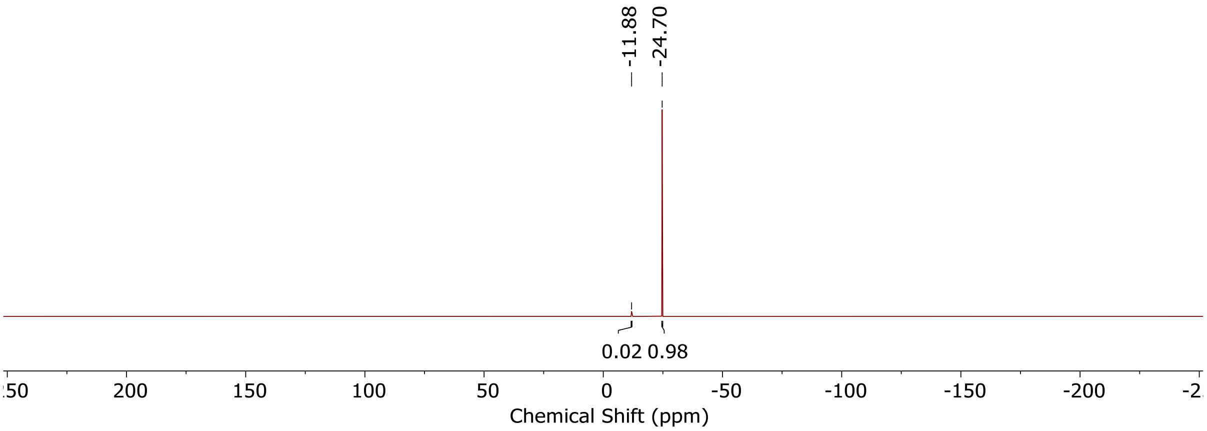

Figure S27. $^{31}P\{^{1}H\}$ NMR spectrum (pyridine) of crude **3b/4b**.

3.1.3. **3c/4c** – (*E*/*Z*)-(4-methylstyryl)diphenylphosphane

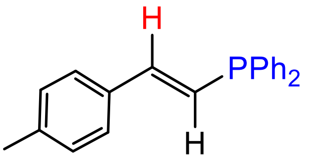

3c

(*E*)-(4-methylstyryl)diphenylphosphane

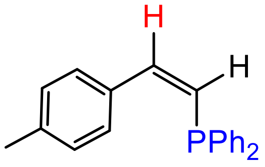

4c

(*Z*)-(4-methylstyryl)diphenylphosphane

Conversion: 82%; **3c** – 6%, **4c** – 76%.

^{1}H NMR (400 MHz, 298 K, pyridine): δ = 2.19 (s, 3H, C*H*$_3$, **4c**), 6.57 (dd, $^{3}J_{HH}$ = 12.95 Hz, $^{3}J_{HP}$ = 2.39 Hz, 1H, C*H*=CHPPh$_2$, **4c**), 7.12 (d, $^{3}J_{HH}$ = 7.96 Hz, 2H, Ar–*H*, **3c/4c**), 7.32–7.39 (m, 10H, Ar–*H*, **3c/4c**), 7.48 (dd, $^{2}J_{HP}$ = 23.77 Hz, $^{3}J_{HH}$ = 12.95 Hz, 1H, CH=C*H*PPh$_2$, **4c**), 7.67 (d, $^{3}J_{HH}$ = 7.24 Hz, 2H, Ar–*H*, **3c/4c**). *Note – **3c** CH=CH resonances could not be identified, too low conversion.*

$^{31}P\{^1H\}$ NMR (162 MHz, 298 K, pyridine): δ = –11.92 (s, **3c**), –24.84 (s, **4c**).

Mass spectrometry (APCI): $C_{21}H_{19}P$+H ([M+H]$^+$); Calcd. = 303.1297, Found = 303.1287.

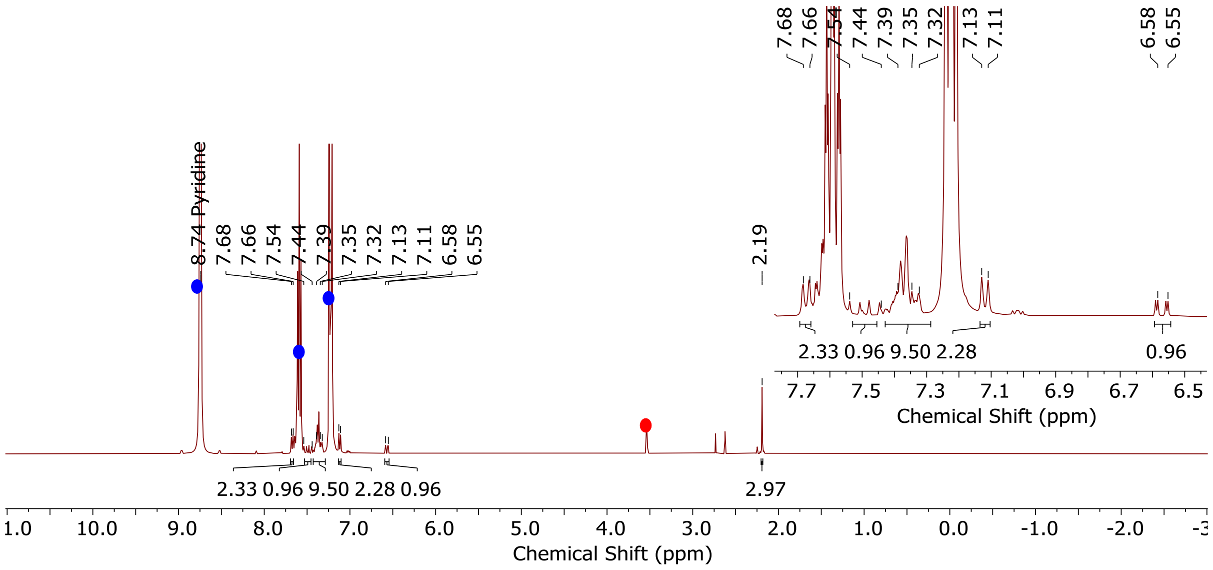

Figure S28. ^{1}H NMR spectrum (pyridine) of crude **3c**/**4c**. ● = pyridine, ● = 18-c-6.

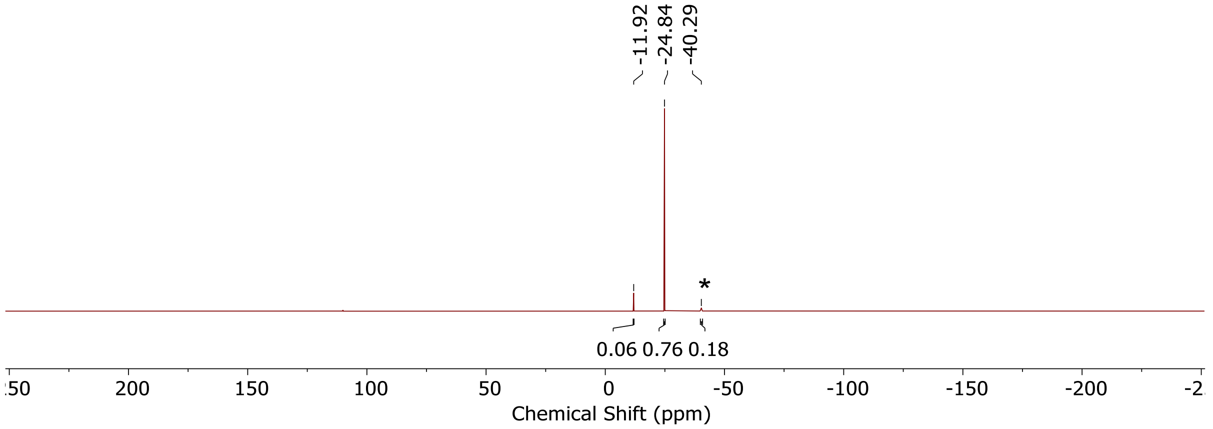

Figure S29. $^{31}P\{^1H\}$ NMR spectrum (pyridine) of crude **3c**/**4c**. * denotes unreacted $HPPh_2$.

3.1.4. **3d**/**4d** – (*E*/*Z*)-diphenyl(4-(trifluoromethyl)styryl)phosphane

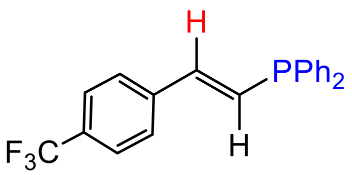

3d
(*E*)-diphenyl(4-(trifluoromethyl)styryl) phosphane

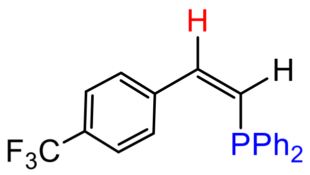

4d
(*Z*)-diphenyl(4-(trifluoromethyl)styryl) phosphane

Conversion: >99%; **3d** – 31%, **4d** – 69%.

^{1}H NMR (400 MHz, 298 K, pyridine): δ = 6.77 (dd, $^{3}J_{HH}$ = 12.77 Hz, $^{3}J_{HP}$ = 2.34 Hz, 1H, C*H*=CHPPh$_2$, **4d**), 7.35–7.43 (m, 9H, Ar–*H*, **3d**/**4d**), 7.48 (dd, $^{2}J_{HP}$ = 23.09 Hz, $^{3}J_{HH}$ = 12.77 Hz, 1H, CH=C*H*PPh$_2$, **4d**), 7.63–7.67 (m, 3H, Ar–*H*, **3d**/**4d**), 7.74 (d, $^{3}J_{HH}$ = 8.05 Hz, 2H, Ar–*H*, **3d**/**4d**). *Note – **3d** CH=CH resonances could not be identified, obscured by solvent resonances.*

^{19}F{^{1}H} NMR (376 MHz, 298 K, pyridine): δ = –63.91 (s, **3d**), –64.05 (s, **4d**).

^{31}P{^{1}H} NMR (162 MHz, 298 K, pyridine): δ = –11.32 (s, **3d**), –25.20 (s, **4d**).

Mass spectrometry (APCI): $C_{21}H_{16}F_3P$+H ([M+H]$^+$); Calcd. = 357.1014, Found = 357.1002.

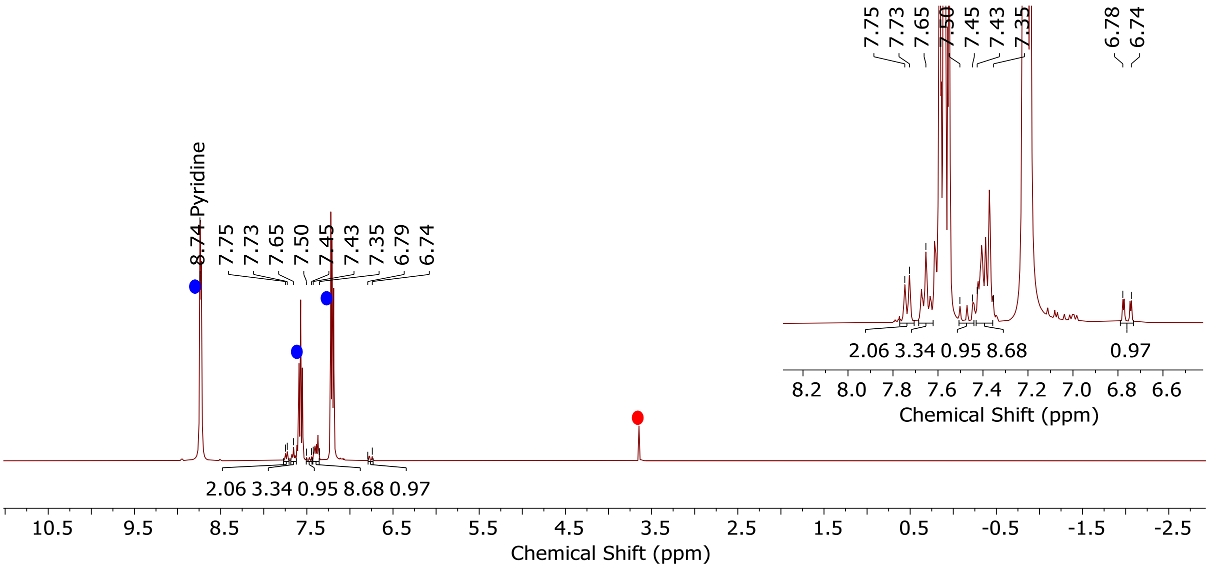

Figure S30. ^{1}H NMR spectrum (pyridine) of crude **3d**/**4d**. ● = pyridine, ● = 18-c-6.

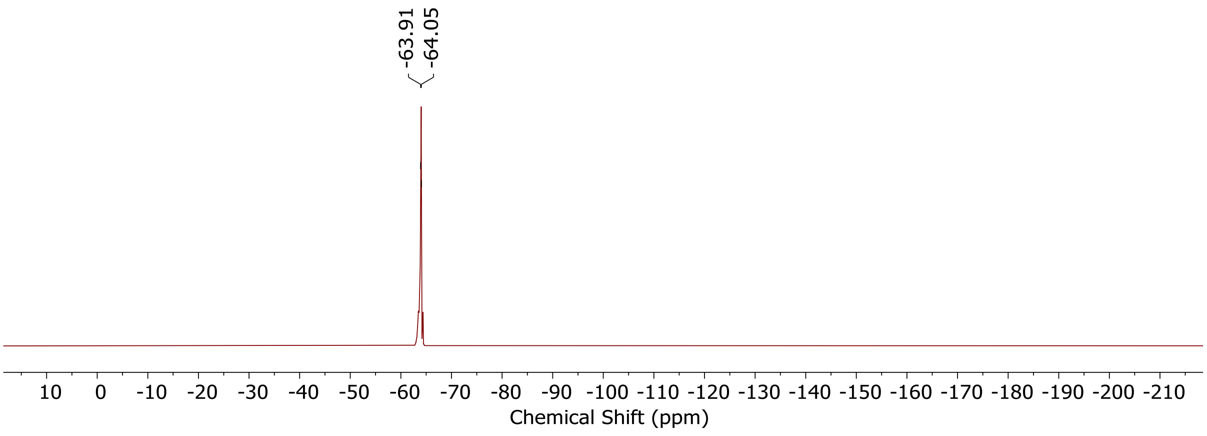

Figure S31. ^{19}F{^{1}H} NMR spectrum (pyridine) of crude **3d**/**4d**.

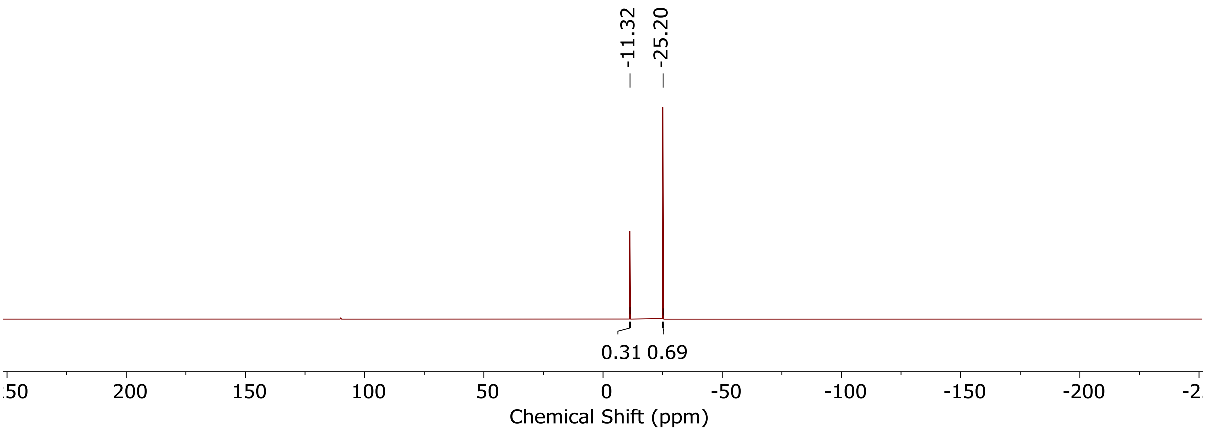

Figure S32. $^{31}P\{^{1}H\}$ NMR spectrum (pyridine) of crude **3d/4d**.

3.1.5. **3e**/**4e** – (*E*/*Z*)-(4-fluorostyryl)diphenylphosphane

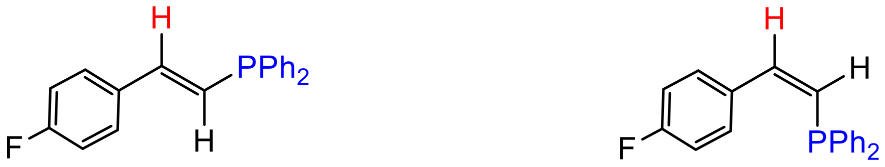

3e (*E*)-(4-fluorostyryl)diphenylphosphane

4e (*Z*)-(4-fluorostyryl)diphenylphosphane

Conversion: >99%; **3e** – 5%, **4e** – 95%.

^{1}H NMR (400 MHz, 298 K, pyridine): δ = 6.59 (dd, $^{3}J_{HH}$ = 12.60 Hz, $^{3}J_{HP}$ = 2.23 Hz, 1H, C*H*=CHPPh$_2$, **4e**), 7.08 (m, 3H, Ar–*H*, **3e**/**4e**), 7.33–7.40 (m, 9H, Ar–*H*, **3e**/**4e**), 7.46 (dd, $^{2}J_{HP}$ = 24.56 Hz, $^{3}J_{HH}$ = 12.60 Hz, 1H, CH=C*H*PPh$_2$, **4e**), 7.66–7.70 (m, 2H, Ar–*H*, **3e**/**4e**). *Note – **3e** CH=CH resonances could not be identified, too low conversion.*

$^{19}F\{^{1}H\}$ NMR (376 MHz, 298 K, pyridine): δ = –112.85 (s, **3e**), –113.21 (s, **4e**).

$^{31}P\{^{1}H\}$ NMR (162 MHz, 298 K, pyridine): δ = –11.90 (s, **3e**), –25.49 (s, **4e**).

Mass spectrometry (APCI): $C_{20}H_{16}FP$+H ([M+H]$^+$); Calcd. = 307.1046, Found = 307.1039.

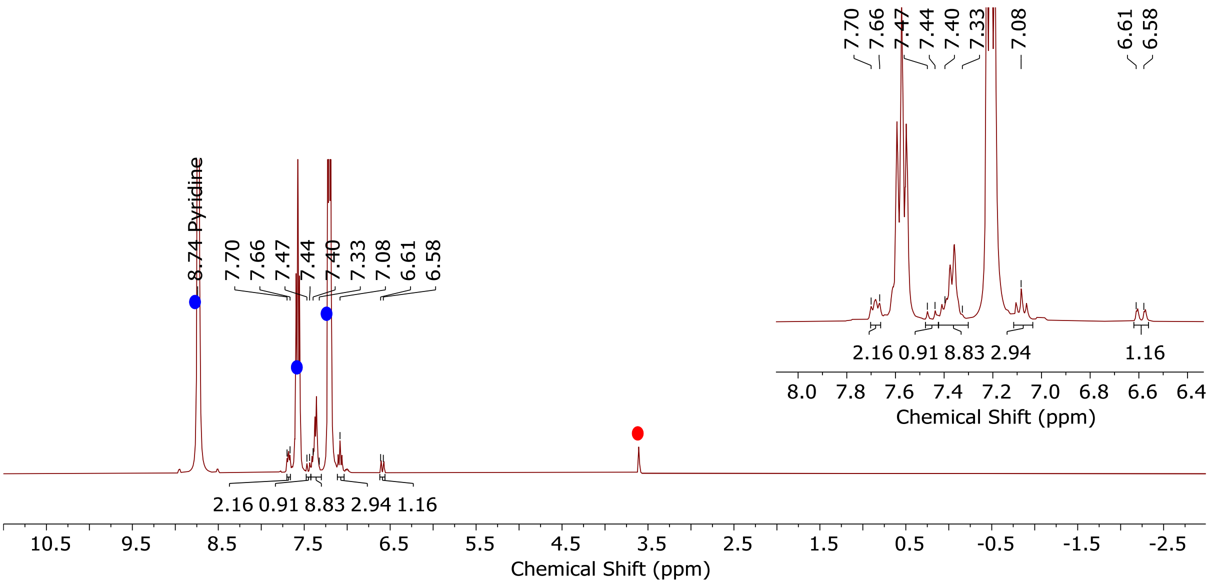

Figure S33. ^{1}H NMR spectrum (pyridine) of crude **3e**/**4e**. ● = pyridine, ● = 18-c-6.

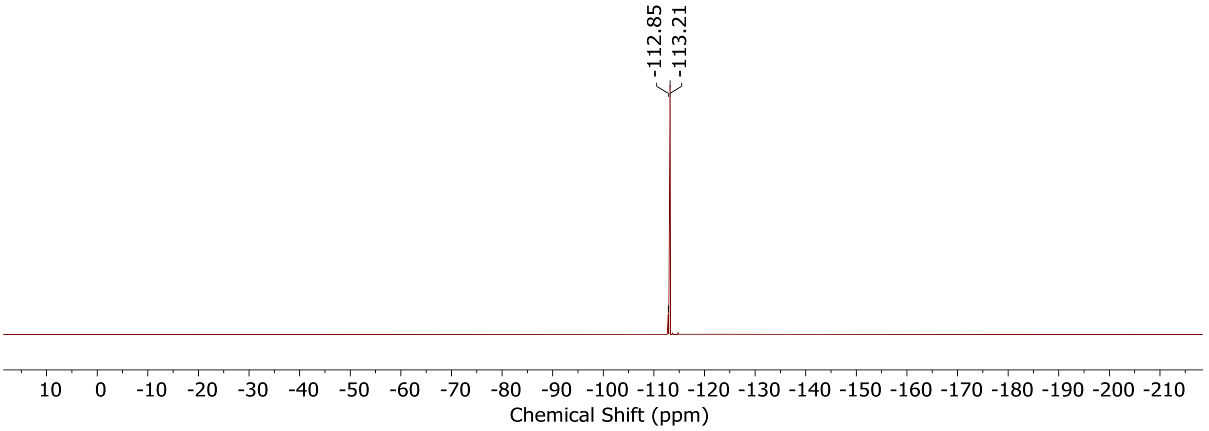

Figure S34. ^{19}F{^{1}H} NMR spectrum (pyridine) of crude **3e**/**4e**.

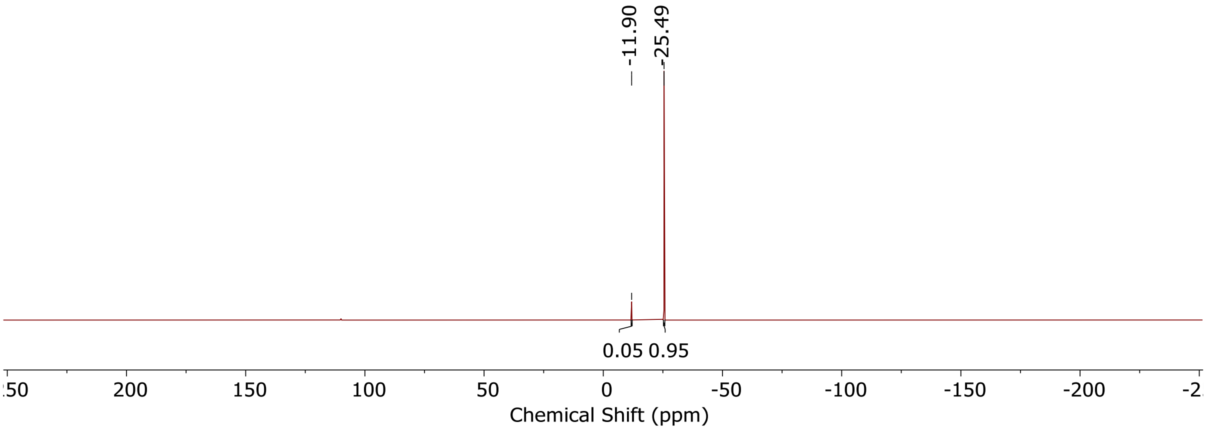

Figure S35. ^{31}P{^{1}H} NMR spectrum (pyridine) of crude **3e**/**4e**.

3.1.6. **3f**/**4f** – (*E*/*Z*)-(4-chlorostyryl)diphenylphosphane

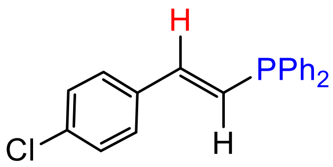

3f
(*E*)-(4-chlorostyryl)diphenylphosphane

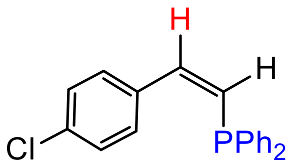

4f
(*Z*)-(4-chlorostyryl)diphenylphosphane

Conversion: 91%; **3f** – 26%, **4f** – 65%.

^{1}H NMR (400 MHz, 298 K, pyridine): δ = 6.62 (dd, $^3J_{HH}$ = 12.52 Hz, $^3J_{HP}$ = 2.55 Hz, 1H, C*H*=CHPPh$_2$, **4f**), 7.29–7.47 (m, 15H, Ar–*H*, **3f**/**4f**, C*H*=CHPPh$_2$, **4f**). *Note – **3f** CH=CH resonances could not be identified, obscured by solvent resonances.*

^{31}P{^{1}H} NMR (162 MHz, 298 K, pyridine): δ = –11.67 (s, **3f**), –25.33 (s, **4f**).

Mass spectrometry (APCI): $C_{20}H_{16}ClP$+H ([M+H]$^+$); Calcd. = 323.0751, Found = 323.0745.

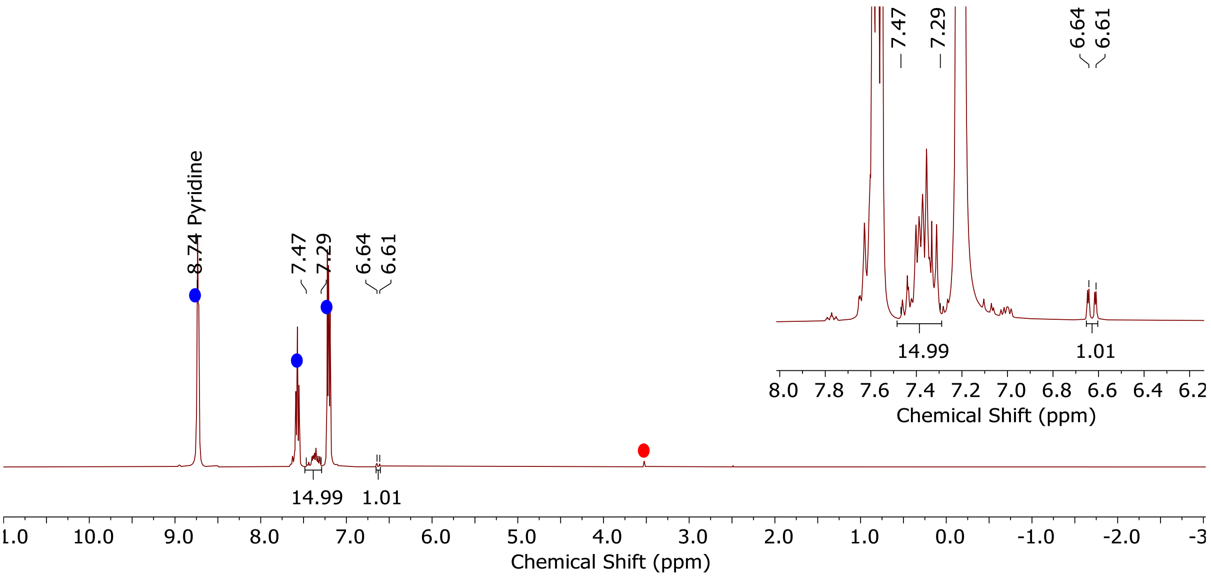

Figure S36. ^{1}H NMR spectrum (pyridine) of crude **3f**/**4f**. ● = pyridine, ● = 18-c-6.

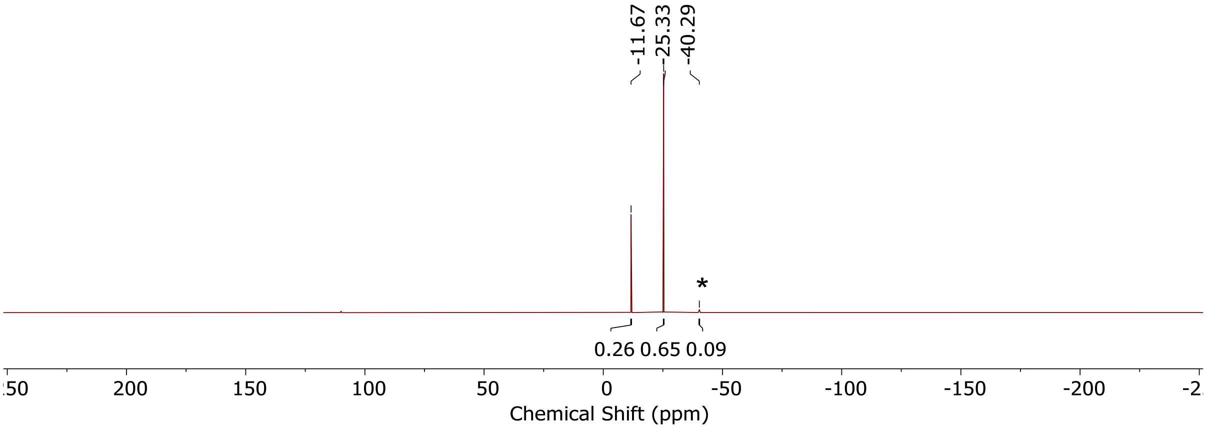

Figure S37. $^{31}P\{^1H\}$ NMR spectrum (pyridine) of crude **3f**/**4f**. * denotes unreacted $HPPh_2$.

3.1.7. **3g**/**4g** – (*E*/*Z*)-(4-bromostyryl)diphenylphosphane

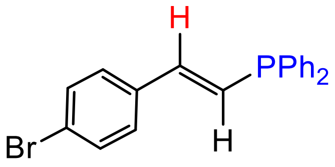

3g
(*E*)-(4-bromostyryl)diphenylphosphane

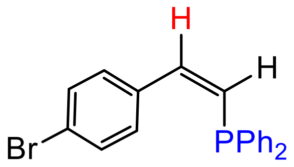

4g
(*Z*)-(4-bromostyryl)diphenylphosphane

Conversion: 90%; **3g** – 27%, **4g** – 62%.

^{1}H NMR (400 MHz, 298 K, pyridine): δ = 6.64 (dd, $^3J_{HH}$ = 12.42 Hz, $^3J_{HP}$ = 2.45 Hz, 1H, C*H*=CHPPh$_2$, **4g**), 7.32–7.48 (m, 15H, Ar–*H*, **3f**/**4f**, C*H*=CHPPh$_2$, **4g**). *Note – **3g** CH=CH resonances could not be identified, obscured by solvent resonances.*

^{31}P{^{1}H} NMR (162 MHz, 298 K, pyridine): δ = –11.62 (s, **3g**), –25.38 (s, **4g**).

Mass spectrometry (APCI): $C_{20}H_{16}BrP+H$ ([M+H]$^+$); Calcd. = 367.0246, Found = 367.0240.

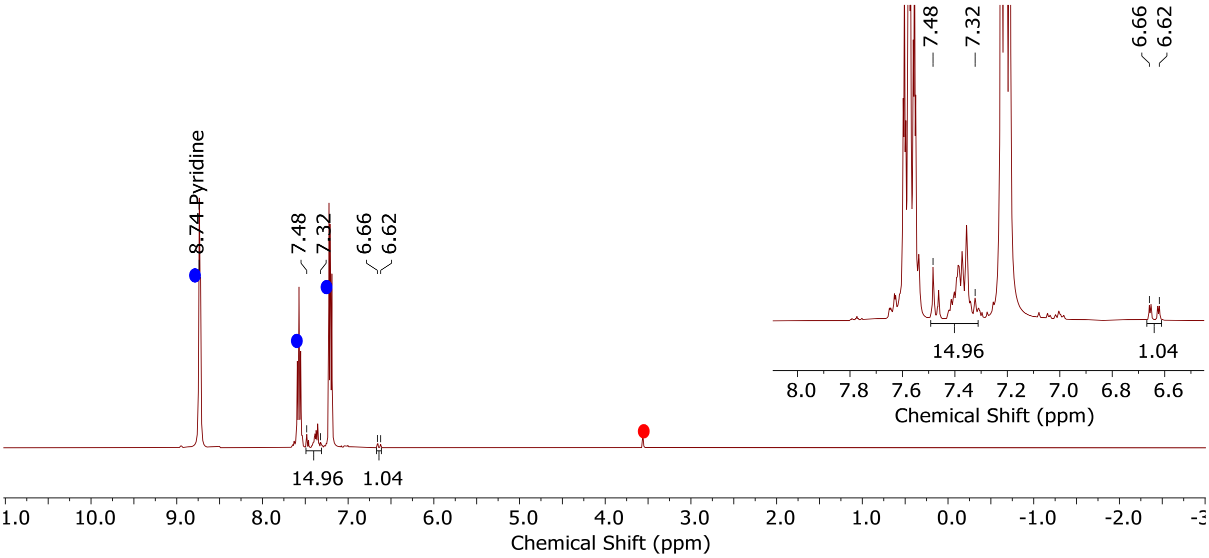

Figure S38. ^{1}H NMR spectrum (pyridine) of crude **3f**/**4f**. ● = pyridine, ● = 18-c-6.

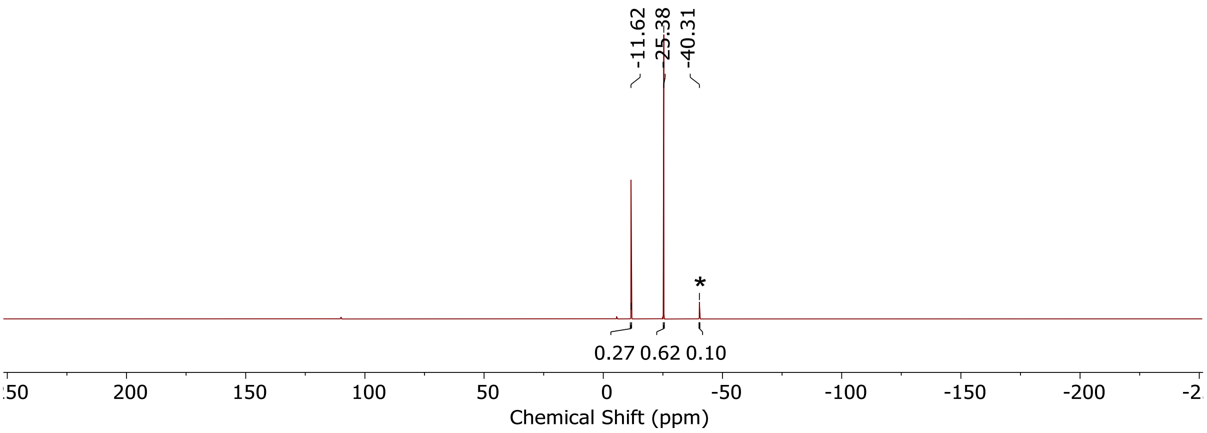

Figure S39. $^{31}P\{^1H\}$ NMR spectrum (pyridine) of crude **3f/4f**. * denotes unreacted $HPPh_2$.

3.1.8. **4h** – (*Z*)-4-(2-(diphenylphosphaneyl)vinyl)aniline

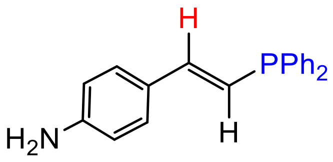

3h
(*E*)-4-(2-(diphenylphosphaneyl)vinyl)aniline

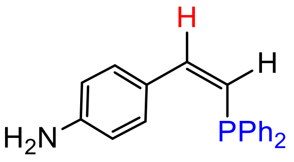

4h
(*Z*)-4-(2-(diphenylphosphaneyl)vinyl)aniline

Conversion: >99%; **3h** – 0%, **4h** – 100%.

^{1}H NMR (400 MHz, 298 K, pyridine): δ = 5.89 (s, 2H, NH_2, **4h**), 6.31 (dd, $^3J_{HH}$ = 13.83 Hz, $^3J_{HP}$ = 2.25 Hz, 1H, C*H*=CHPPh$_2$, **4h**), 6.90 (d, $^3J_{HH}$ = 8.43 Hz, 2H, Ar–*H*, **4h**), 7.31–7.38 (m, 10H, Ar–*H*, **4h**), 7.46 (dd, $^2J_{HP}$ = 24.49 Hz, $^3J_{HH}$ = 13.83 Hz, 1H, CH=C*H*PPh$_2$, **4h**), 7.74 (d, $^3J_{HH}$ = 8.43 Hz, 2H, Ar–*H*, **4h**).

^{31}P{^{1}H} NMR (162 MHz, 298 K, pyridine): δ = –24.38 (s, **4h**).

Mass spectrometry (APCI): $C_{20}H_{18}NP$+H ([M+H]$^+$); Calcd. = 304.1250, Found = 304.1243.

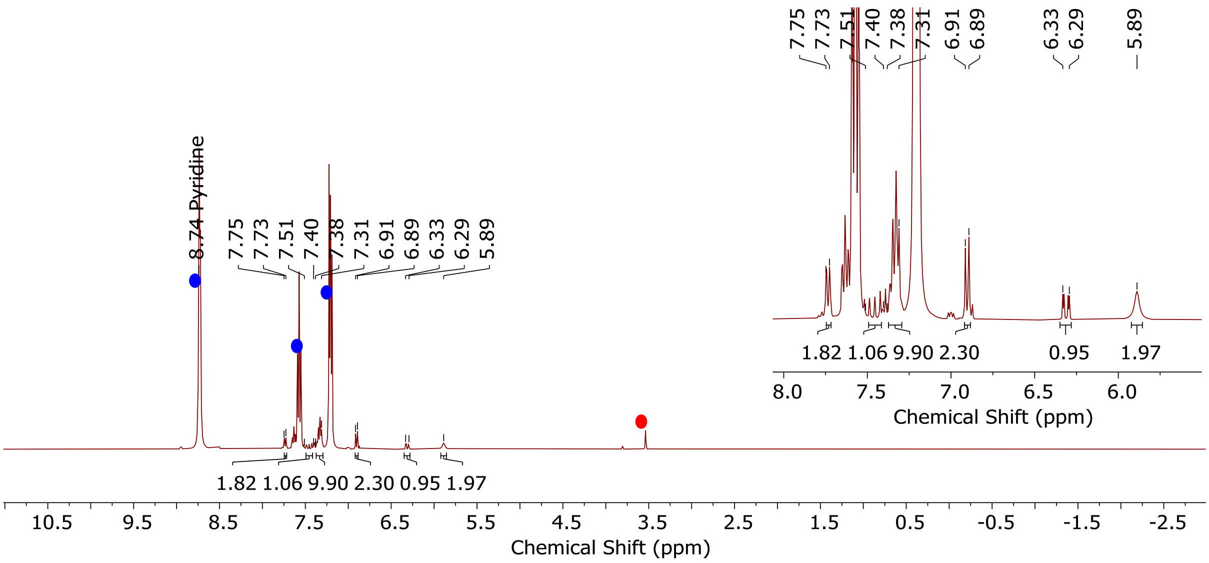

Figure S40. ^{1}H NMR spectrum (pyridine) of crude **4h**. ● = pyridine, ● = 18-c-6.

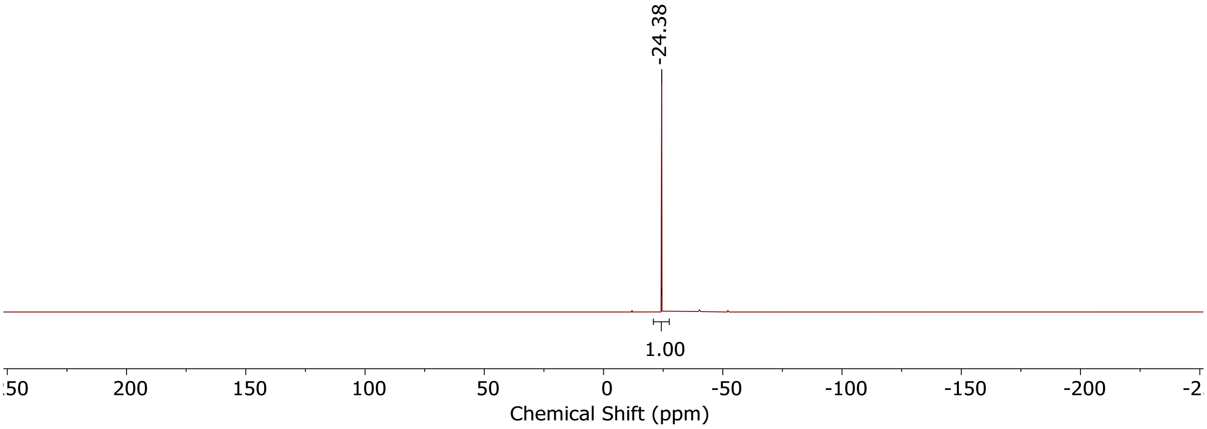

Figure S41. ^{31}P{^{1}H} NMR spectrum (pyridine) of crude **4h**.

3.1.9. **3i**/**4i** – (*E*/*Z*)-(3-methylstyryl)diphenylphosphane

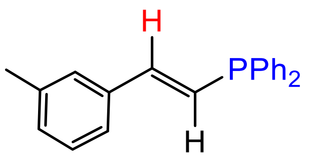

3i

(*E*)-(3-methylstyryl)diphenylphosphane

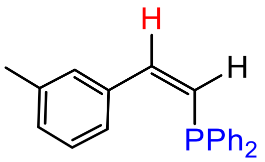

4i

(*Z*)-(3-methylstyryl)diphenylphosphane

Conversion: >99%; **3i** – 13%, **4i** – 87%.

^{1}H NMR (400 MHz, 298 K, pyridine): δ = 2.20 (s, 3H, C*H*$_3$, **4i**), 2.25 (s, 3H(13%) observed integration 0.39, C*H*$_3$, **3i**), 6.62 (dd, $^{3}J_{HH}$ = 12.62 Hz, $^{3}J_{HP}$ = 2.52 Hz, 1H, C*H*=CHPPh$_2$, **4i**), 7.01 (s, 1H, Ar–*H*, **3i**/**4i**), 7.33–7.51 (m, 11H, Ar–*H*, **3i**/**4i**, CH=C*H*PPh$_2$, **4i**), 7.61–7.65 (m, 3H, Ar–*H*, **3i**/**4i**). *Note – **3i** CH=CH resonances could not be identified, obscured by solvent resonances.*

^{31}P{^{1}H} NMR (162 MHz, 298 K, pyridine): δ = –11.81 (s, **3i**), –24.81 (s, **4i**).

Mass spectrometry (APCI): $C_{21}H_{19}P+H$ ($[M+H]^+$); Calcd. = 303.1297, Found = 303.1287.

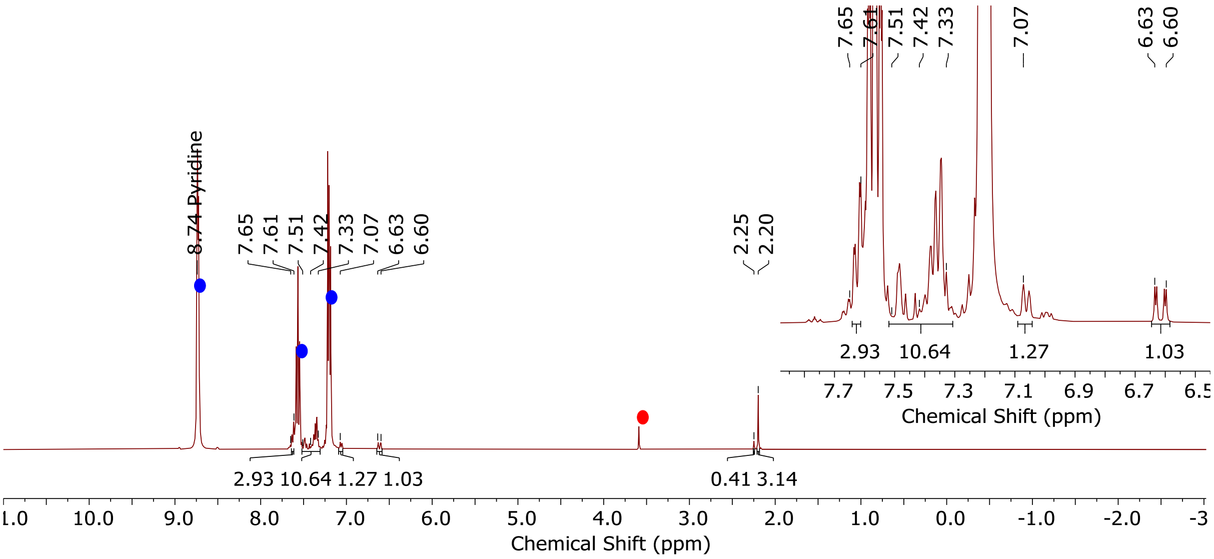

Figure S42. ^{1}H NMR spectrum (pyridine) of crude **3i**/**4i**. ● = pyridine, ● = 18-c-6.

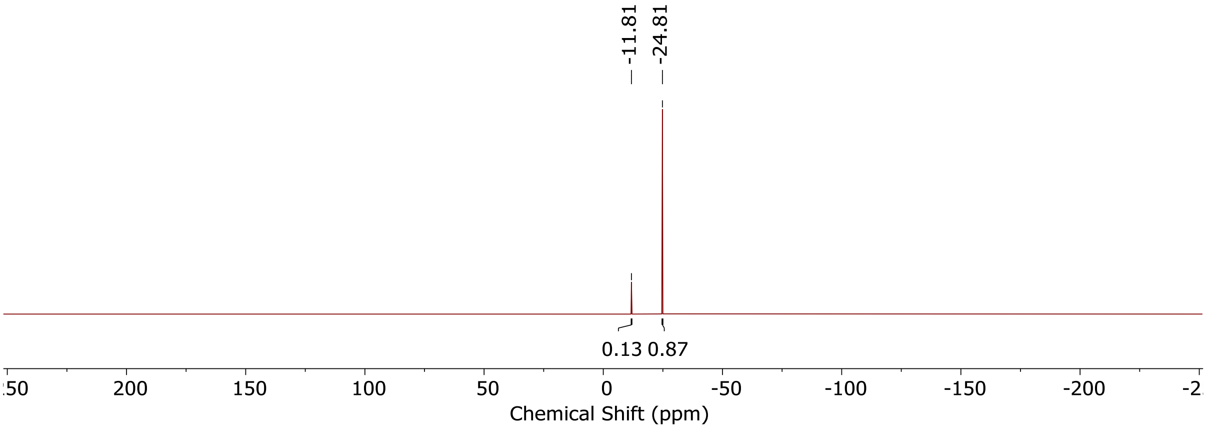

Figure S43. ^{31}P{^{1}H} NMR spectrum (pyridine) of crude **3i**/**4i**.

3.1.10. **3j**/**4j** – (*E*/*Z*)-(2-methylstyryl)diphenylphosphane

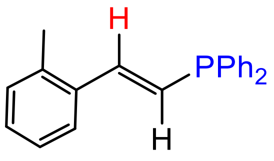

3j

(*E*)-(2-methylstyryl)diphenylphosphane

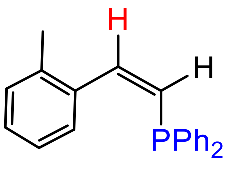

4j

(*Z*)-(2-methylstyryl)diphenylphosphane

Conversion: >99%; **3j** – 16%, **4j** – 84%.

^{1}H NMR (400 MHz, 298 K, pyridine): δ = 2.24 (s, 3H(16%) observed integration 0.48, C*H*$_3$, **3j**), 2.28 (s, 3H, C*H*$_3$, **4j**), 6.73 (dd, $^3J_{HH}$ = 12.43 Hz, $^3J_{HP}$ = 2.87 Hz, 1H, C*H*=CHPPh$_2$, **4j**), 7.15–7.19 (m, 4H, Ar–*H*, **3j**/**4j**), 7.34–7.55 (m, 9H, Ar–*H*, **3j**/**4j**,

CH=C*H*PPh$_2$, **4j**), 7.64–7.69 (m, 2H, Ar–*H*, **3j**/**4j**). *Note – **3j** CH=CH resonances could not be identified, obscured by solvent resonances.*

^{31}P{^{1}H} NMR (162 MHz, 298 K, pyridine): δ = –12.33 (s, **3j**), –27.99 (s, **4j**).

Mass spectrometry (APCI): $C_{21}H_{19}P$+H ([M+H]$^+$); Calcd. = 303.1297, Found = 303.1289.

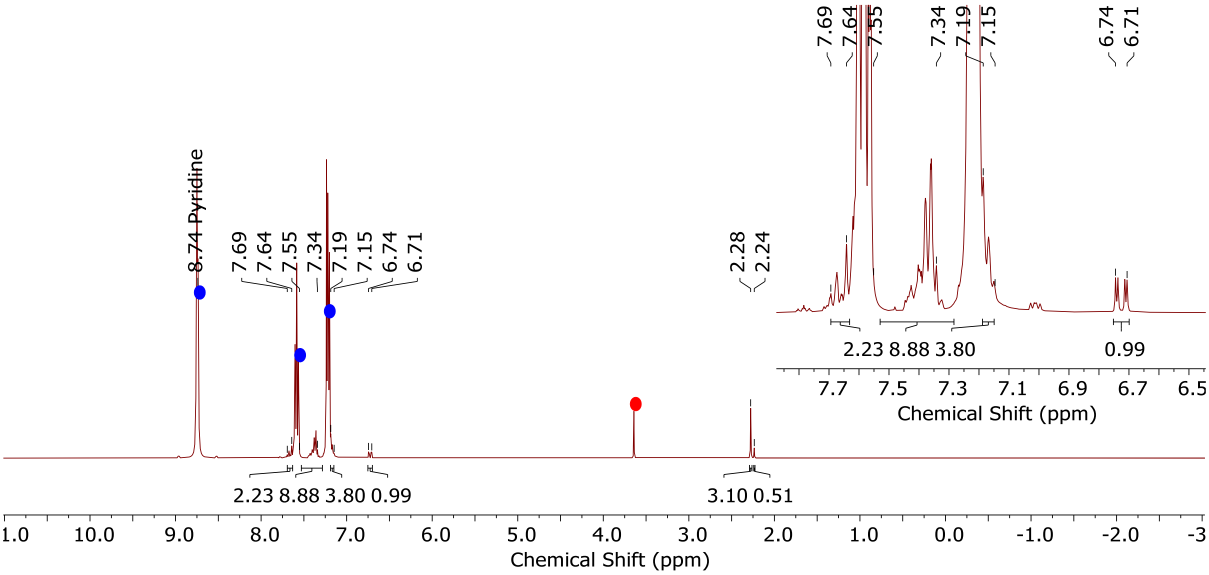

Figure S44. ^{1}H NMR spectrum (pyridine) of crude **3j**/**4j**. ● = pyridine, ● = 18-c-6.

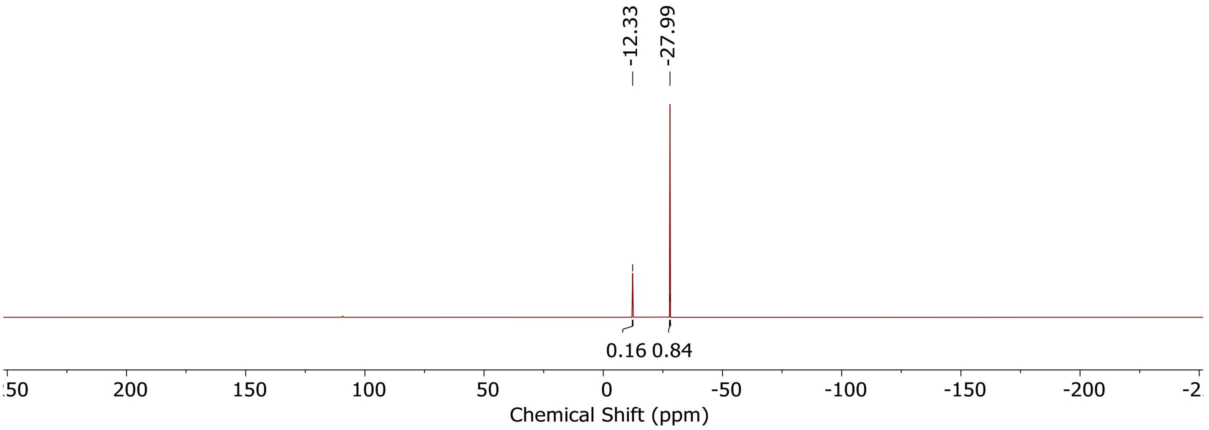

Figure S45. ^{31}P{^{1}H} NMR spectrum (pyridine) of crude **3j**/**4j**.

3.1.11. **3k**/**4k** – (*E*/*Z*)-(2-cyclohexylvinyl)diphenylphosphane

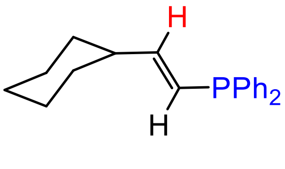

3k

(*E*)-(2-cyclohexylvinyl)diphenylphosphane

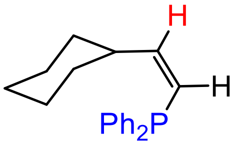

4k

(*Z*)-(2-cyclohexylvinyl)diphenylphosphane

Conversion: >99%; **3k** – 32%, **4k** – 68%.

^{1}H NMR (400 MHz, 298 K, pyridine): δ = 1.04–1.75 (m, 11H, Cy–*H*, **3k**/**4k**), 6.25–6.41 (m, 2H, C*H*=CHPPh$_2$, **4k**, CH=C*H*PPh$_2$, **4k**), 7.28–7.44 (m, 10H, Ar–*H*, **3k**/**4k**). *Note – **3k** CH=CH resonances could not be identified, obscured by solvent resonances.*

^{31}P{^{1}H} NMR (162 MHz, 298 K, pyridine): δ = –5.55 (s, **3k**), –31.52 (s, **4k**).

Mass spectrometry (APCI): $C_{20}H_{23}P$+H ([M+H]$^+$); Calcd. = 295.1610, Found = 295.1612.

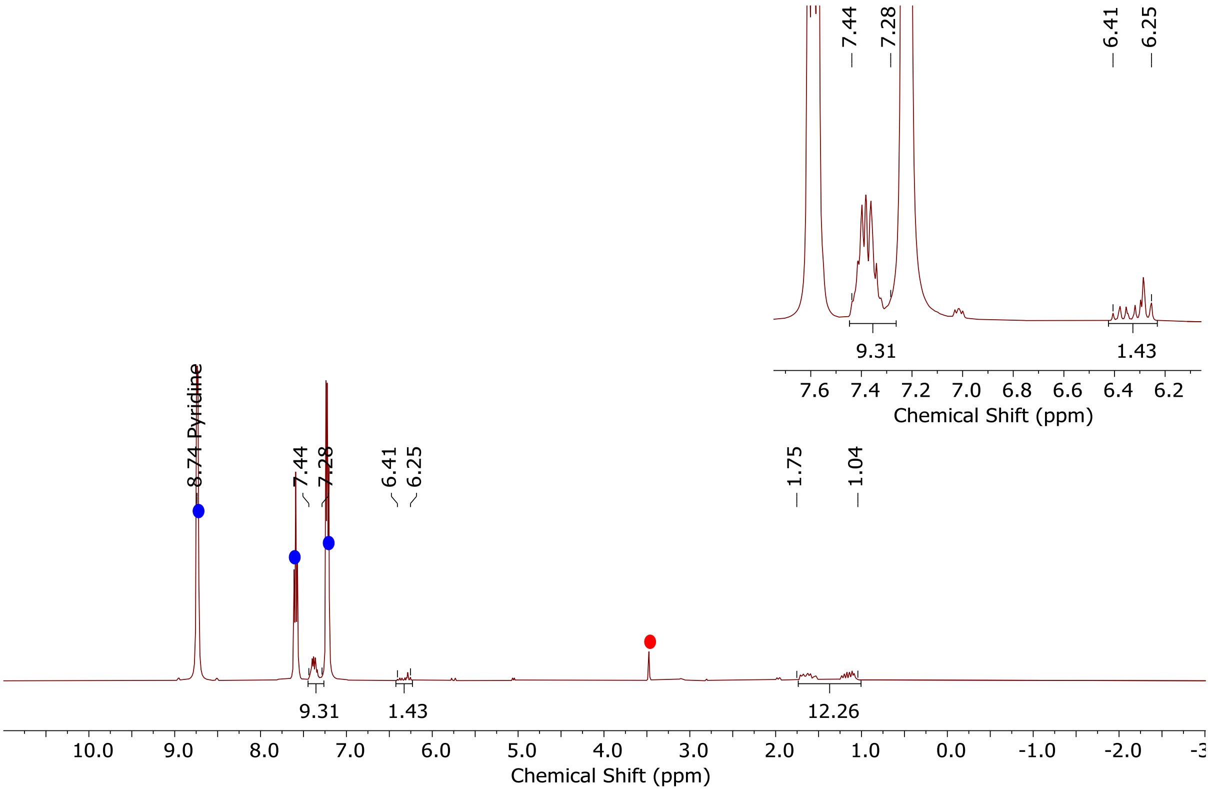

Figure S46. ^{1}H NMR spectrum (pyridine) of crude **3k**/**4k**. ● = pyridine, ● = 18-c-6.

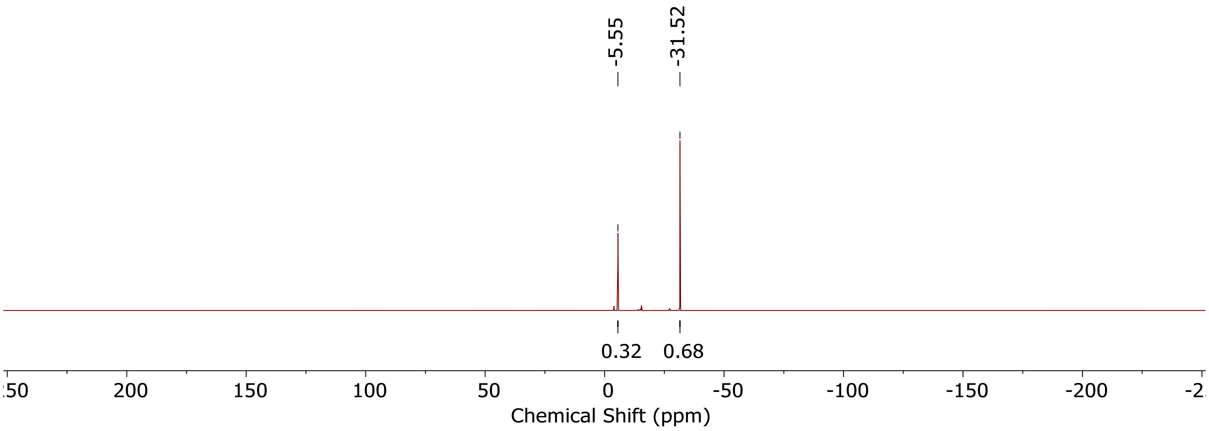

Figure S47. ^{31}P{^{1}H} NMR spectrum (pyridine) of crude **3k**/**4k**.

3.1.12. **3l**/**4l** – (*E*/*Z*)-hex-1-en-1-yldiphenylphosphane

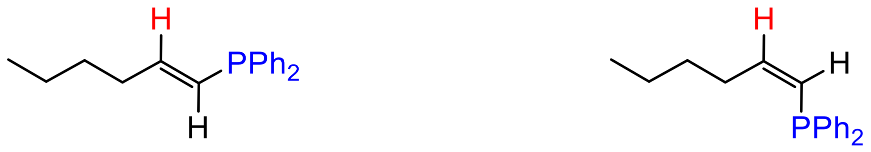

3l
(*E*)-hex-1-en-1-yldiphenylphosphane

4l
(*Z*)-hex-1-en-1-yldiphenylphosphane

Conversion: 96%; **3l** – 47%, **4l** – 49%.

^{1}H NMR (400 MHz, 298 K, pyridine): δ = 0.77–2.51 (m, 9H, C*H*$_3$, C*H*$_2$, **3l**/**4l**), 5.14 (d, $^{3}J_{HH}$ = 8.82 Hz, 1H(49%) observed integration 0.49, C*H*=CHPPh$_2$, **4l**), 5.70 (d, $^{2}J_{HP}$ = 21.00 Hz, 1H(49%) observed integration 0.49, CH=C*H*PPh$_2$, **4l**), 6.33–6.54 (m, 2H(47%) observed integration 0.94, C*H*=CHPPh$_2$, CH=C*H*PPh$_2$, **3l**), 7.33–7.43 (m, 10H, Ar–*H*, **3l**/**4l**).

^{31}P{^{1}H} NMR (162 MHz, 298 K, pyridine): δ = –2.97 (s, **3l**), –31.77 (s, **4l**).

Mass spectrometry (APCI): $C_{18}H_{21}P$+H ([M+H]$^+$); Calcd. = 269.1454, Found = 269.1454.

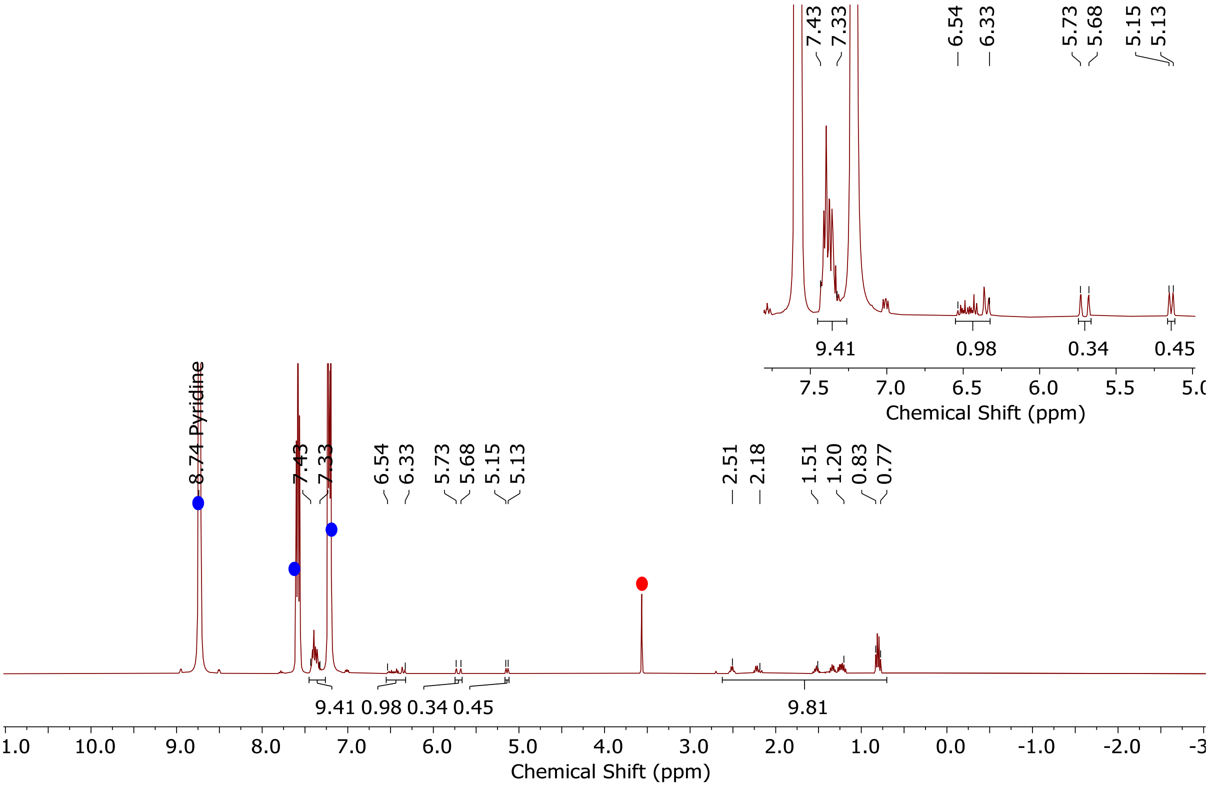

Figure S48. ^{1}H NMR spectrum (pyridine) of crude **3l**/**4l**. ● = pyridine, ● = 18-c-6.

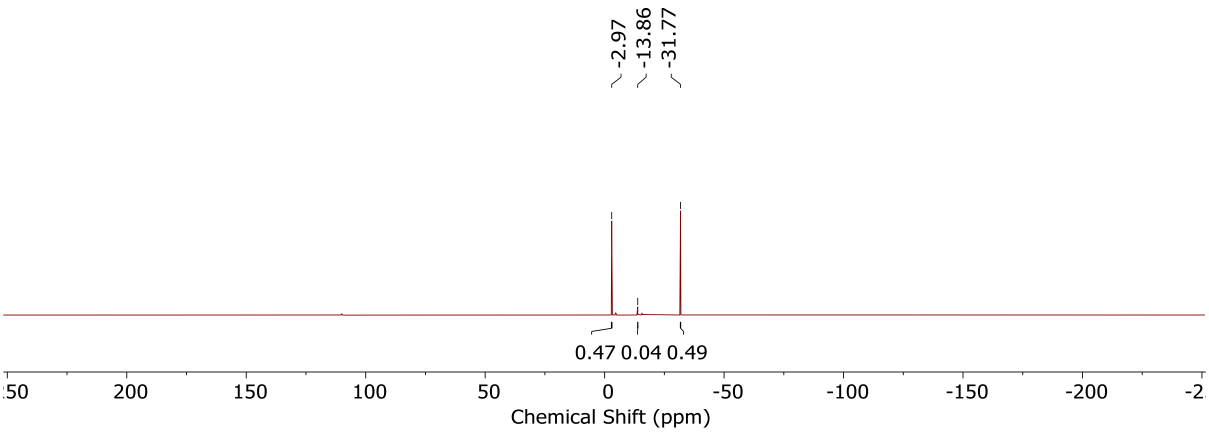

Figure S49. $^{31}P\{^1H\}$ NMR spectrum (pyridine) of crude **3l**/**4l**. Resonance at –13.86 ppm is attributed to $Ph_2P–PPh_2$.[10]

3.1.13. **3m**/**4m** – (*E*/*Z*)-(1,2-diphenylvinyl)diphenylphosphane

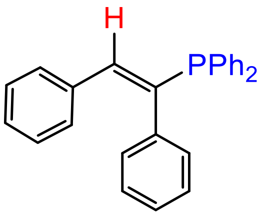

3m
(*E*)-(1,2-diphenylvinyl)diphenylphosphane

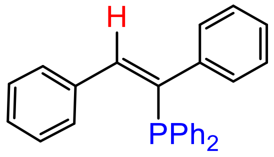

4m
(*Z*)-(1,2-diphenylvinyl)diphenylphosphane

Conversion: >99%; **3m** – 60%, **4m** – 40%.

^{1}H NMR (400 MHz, 298 K, pyridine): δ = 6.83 (d, $^3J_{HP}$ = 8.22 Hz, 1H, *trans*-CH=CPPh$_2$, **4m**), 7.11–7.15 (m, 5H, Ar–*H*, **3m**/**4m**), 7.35–7.46 (m, 10H, Ar–*H*, **3m**/**4m**), 7.68–7.72 (m, 5H, Ar–*H*, **3m**/**4m**), 7.84 (d, $^3J_{HP}$ = 7.78 Hz, 1H, *cis*-CH=C*H*PPh$_2$, **3m**).

^{31}P{^{1}H} NMR (162 MHz, 298 K, pyridine): δ = –2.97 (s, **3m**), –31.77 (s, **4m**).

Mass spectrometry (APCI): $C_{26}H_{21}P$+H ([M+H]$^+$); Calcd. = 365.1454, Found = 365.1438.

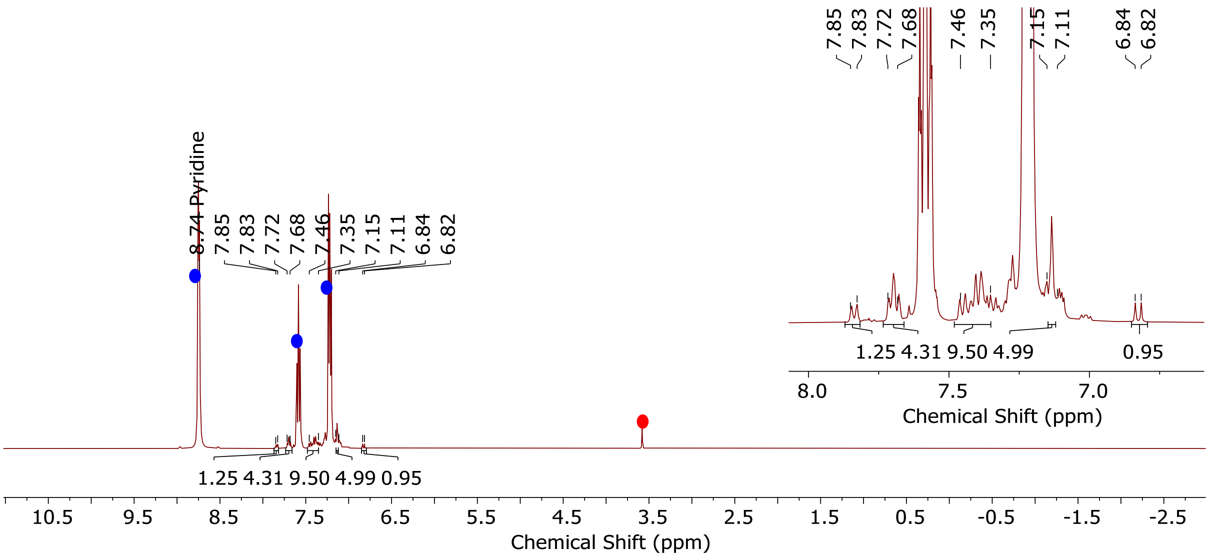

Figure S50. ^{1}H NMR spectrum (pyridine) of crude **3m/4m**. ● = pyridine, ● = 18-c-6.

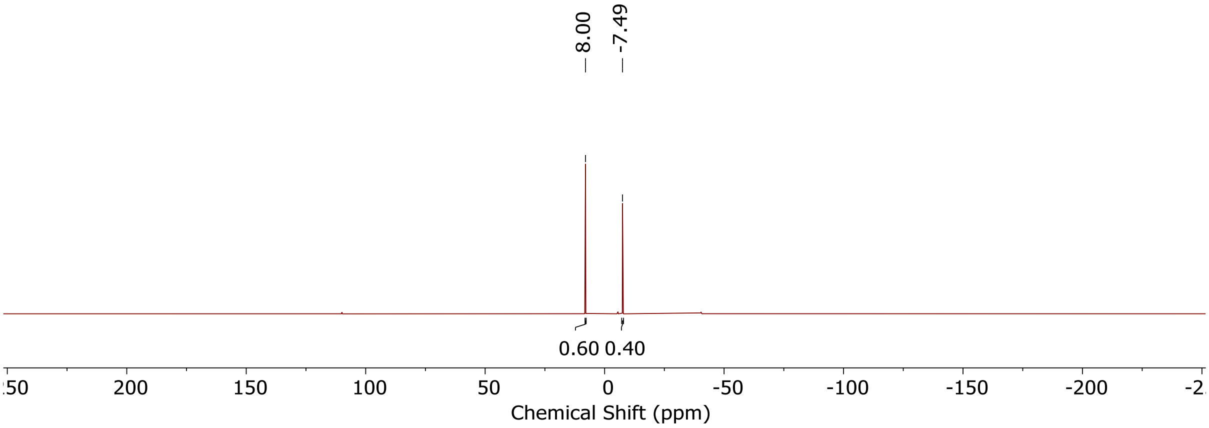

Figure S51. $^{31}P\{^{1}H\}$ NMR spectrum (pyridine) of crude **3m/4m**.

3.2. Products of Alkene Hydrophosphination

3.2.1. **6a** – phenethyldiphenylphosphane

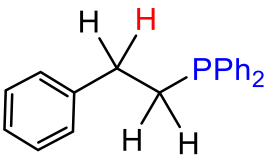

6a
phenethyldiphenylphosphane

Conversion: >99%.

^{1}H NMR (400 MHz, 298 K, pyridine): δ = 2.44–2.48 (m, 2H, CH_2CH$_2$PPh$_2$), 2.76–2.83 (m, 2H, CH$_2$CH_2PPh$_2$), 7.25–7.38 (m, 15H, Ar–*H*).

^{31}P{^{1}H} NMR (162 MHz, 298 K, pyridine): δ = –16.60 (s).

Mass spectrometry (APCI): $C_{20}H_{19}P$+H ([M+H]$^{+}$); Calcd. = 291.1297, Found = 291.1289.

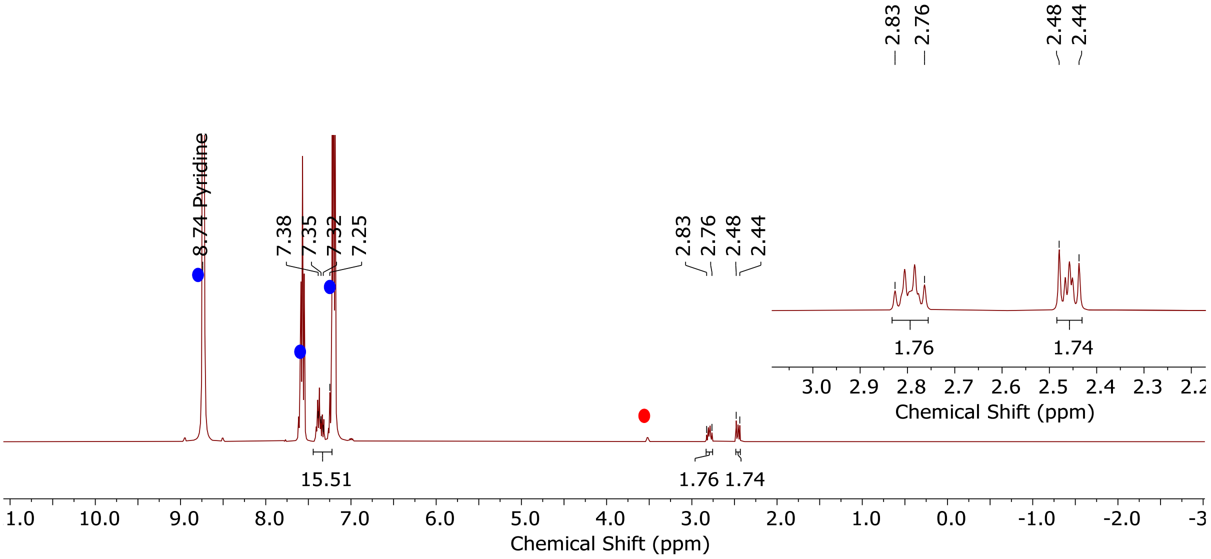

Figure S52. ^{1}H NMR spectrum (pyridine) of crude **6a**. ● = pyridine, ● = 18-c-6.

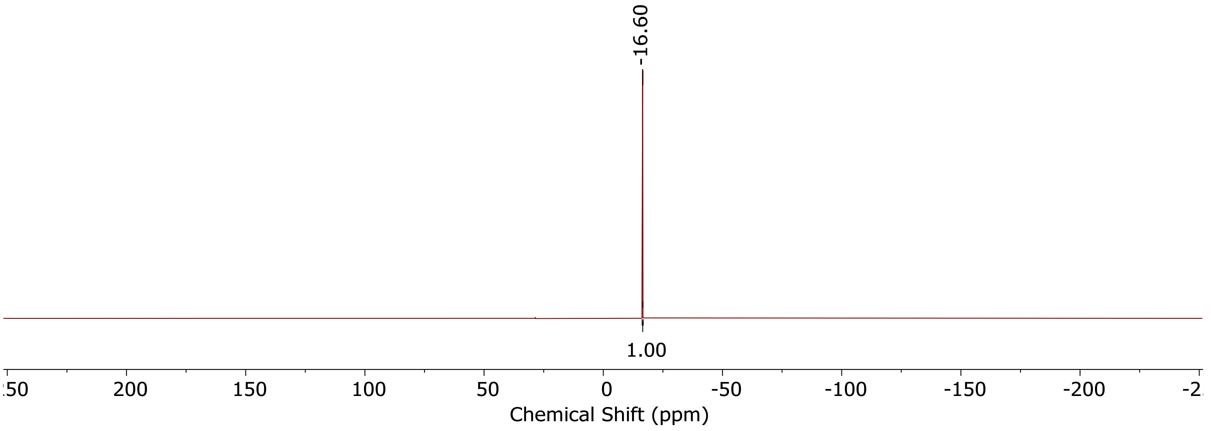

Figure S53. ^{31}P{^{1}H} NMR spectrum (pyridine) of crude **6a**.

3.2.2. **6b** – (4-methoxyphenethyl)diphenylphosphane

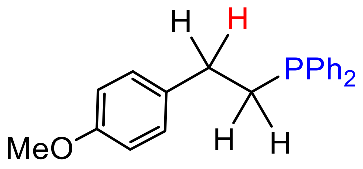

6b

(4-methoxyphenethyl)diphenylphosphane

Conversion: >99%

^{1}H NMR (400 MHz, 298 K, pyridine): δ = 2.47–2.51 (m, 2H, CH_2CH$_2$PPh$_2$), 2.77–2.83 (m, 2H, CH$_2$CH_2PPh$_2$), 3.70 (s, 3H, OCH_3), 6.99–7.02 (m, 2H, Ar–*H*), 7.32–7.45 (m, 8H, Ar–*H*), 7.63–7.65 (m, 4H, Ar–*H*).

^{31}P{^{1}H} NMR (162 MHz, 298 K, pyridine): δ = –16.63 (s).

Mass spectrometry (APCI): $C_{21}H_{21}OP+H$ ($[M+H]^+$); Calcd. = 321.1403, Found = 321.1389.

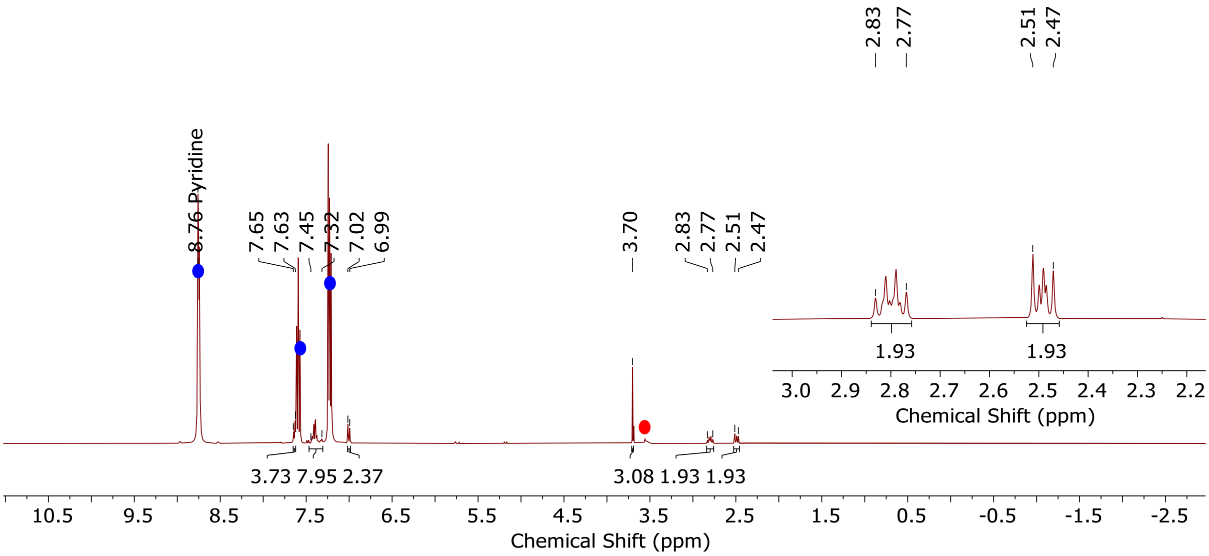

Figure S54. ^{1}H NMR spectrum (pyridine) of crude **6b**. ● = pyridine, ● = 18-c-6.

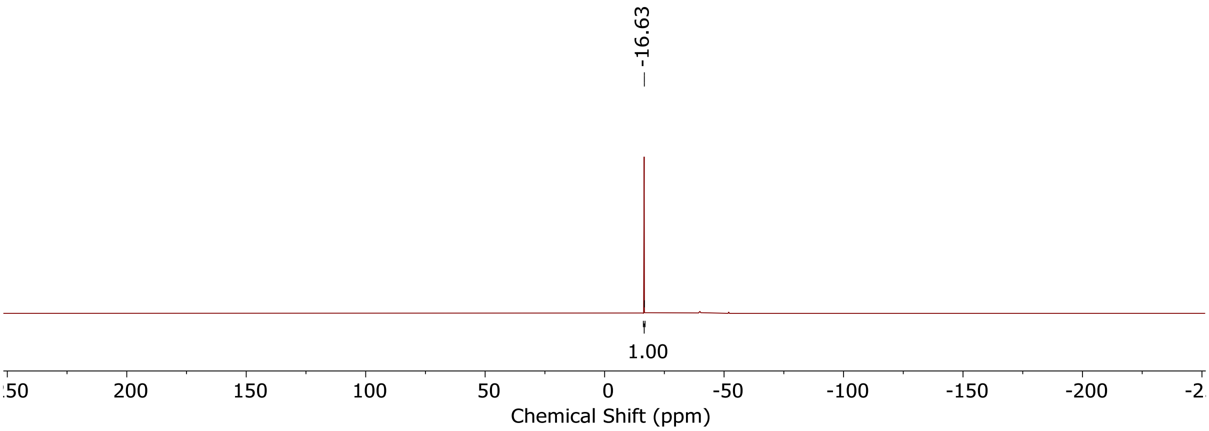

Figure S55. $^{31}P\{^{1}H\}$ NMR spectrum (pyridine) of crude **6b**.

3.2.3. **6c** – (4-methylphenethyl)diphenylphosphane

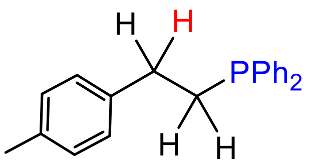

6c

(4-methylphenethyl)diphenylphosphane

Conversion: >99%.

^{1}H NMR (400 MHz, 298 K, pyridine): δ = 2.25 (s, 3H, C*H*$_3$), 2.45–2.49 (m, 2H, C*H*$_2$CH$_2$PPh$_2$), 2.76–2.82 (m, 2H, CH$_2$C*H*$_2$PPh$_2$), 7.35–7.42 (m, 9H, Ar–*H*), 7.60–7.63 (m, 5H, Ar–*H*).

^{31}P{^{1}H} NMR (162 MHz, 298 K, pyridine): δ = –16.55 (s).

Mass spectrometry (APCI): $C_{21}H_{21}P$+H ($[M+H]^+$); Calcd. = 305.1454, Found = 305.1453.

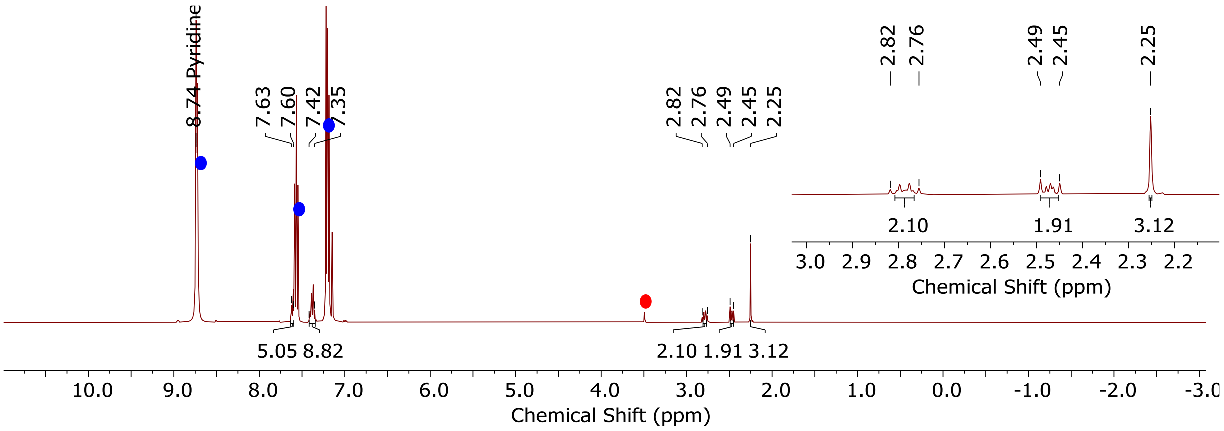

Figure S56. ^1H NMR spectrum (pyridine) of crude **6c**. ● = pyridine, ● = 18-c-6.

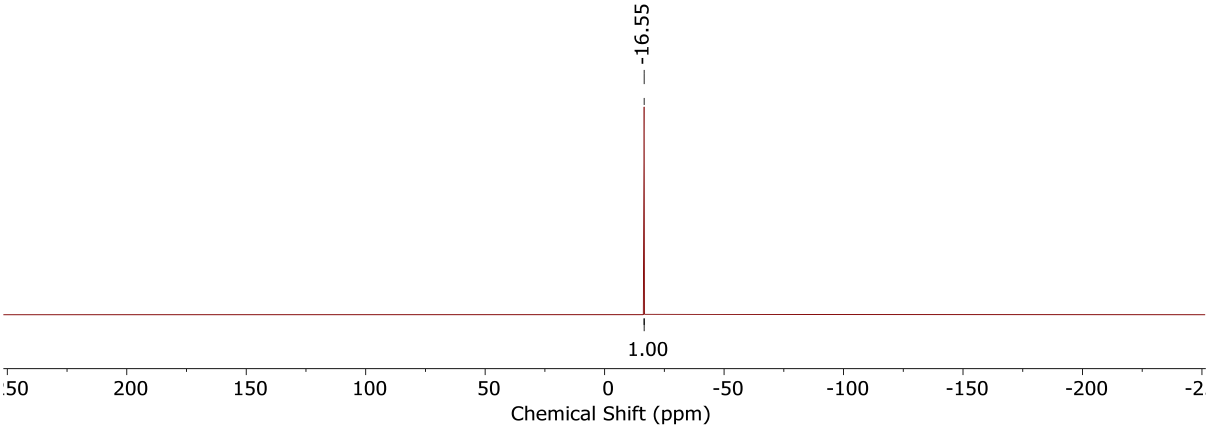

Figure S57. $^{31}P\{^1H\}$ NMR spectrum (pyridine) of crude **6c**.

3.2.4. **6d** – (4-fluorophenethyl)diphenylphosphane

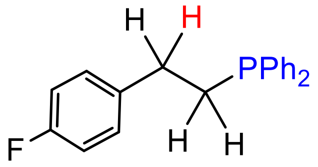

6d
(4-fluorophenethyl)diphenylphosphane

Conversion: >99%.

^{1}H NMR (400 MHz, 298 K, pyridine): δ = 2.41–2.45 (m, 2H, CH_2CH$_2$PPh$_2$), 2.71–2.78 (m, 2H, CH$_2$CH_2PPh$_2$), 7.07–7.11 (m, 5H, Ar–*H*), 7.34–7.42 (m, 7H, Ar–*H*), 7.60–7.77 (m, 2H, Ar–*H*).

$^{19}F\{^1H\}$ NMR (376 MHz, 298 K, pyridine): δ = –117.35 (s).

$^{31}P\{^1H\}$ NMR (162 MHz, 298 K, pyridine): δ = –16.63 (s).

Mass spectrometry (APCI): $C_{20}H_{18}FP+H$ ($[M+H]^+$); Calcd. = 309.1203, Found = 309.1194.

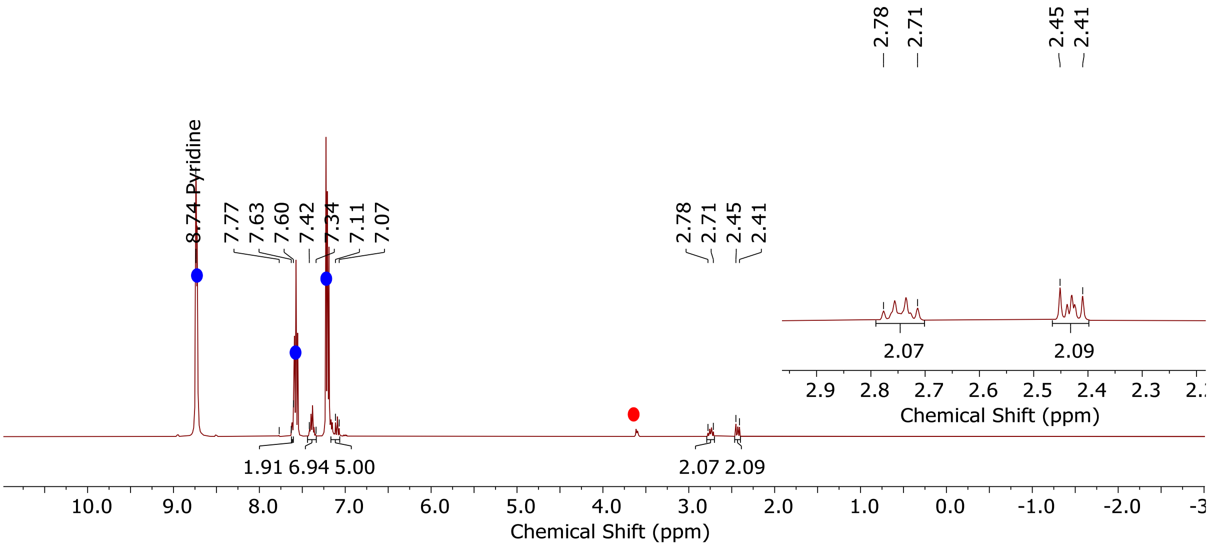

Figure S58. ^{1}H NMR spectrum (pyridine) of crude **6d**. ● = pyridine, ● = 18-c-6.

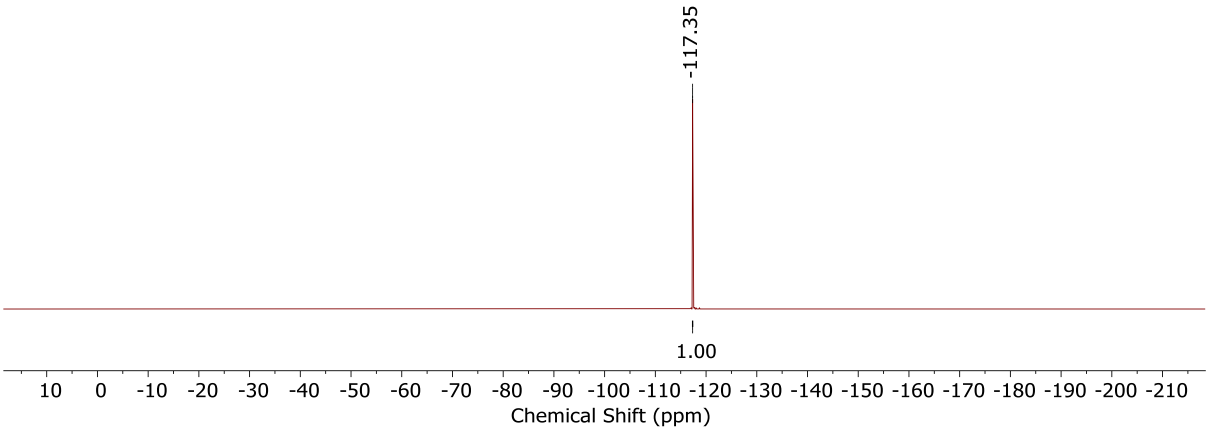

Figure S59. ^{19}F{^{1}H} NMR spectrum (pyridine) of crude **6d**.

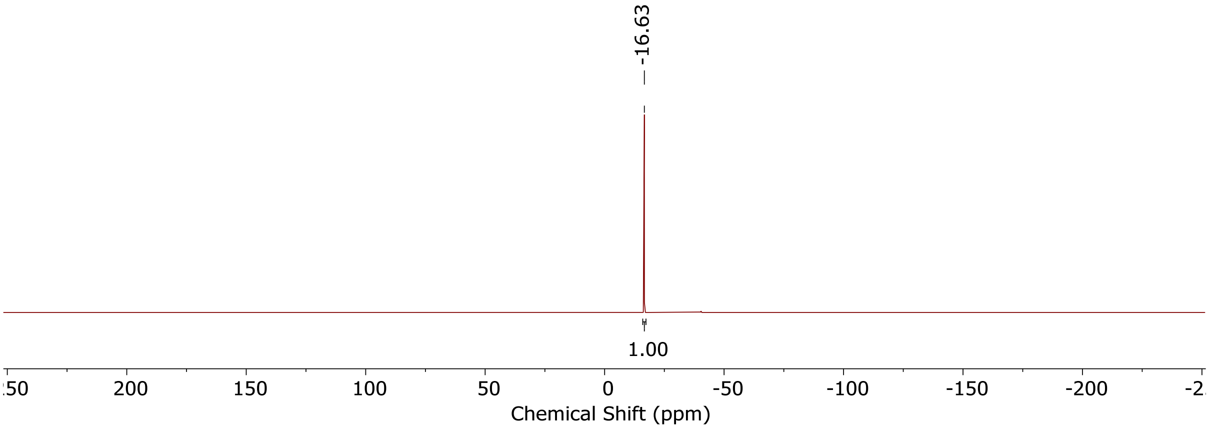

Figure S60. ^{31}P{^{1}H} NMR spectrum (pyridine) of crude **6d**.

3.2.5. **6e** – (4-bromophenethyl)diphenylphosphane

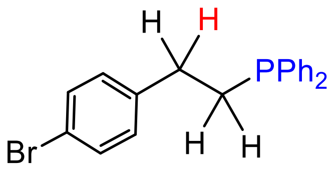

6e

(4-bromophenethyl)diphenylphosphane

Conversion: >99%.

^{1}H NMR (400 MHz, 298 K, pyridine): δ = 2.40–2.44 (m, 2H, CH_2CH$_2$PPh$_2$), 2.68–2.74 (m, 2H, CH$_2$CH_2PPh$_2$), 7.07–7.09 (m, 2H, Ar–*H*), 7.28–7.51 (m, 12H, Ar–*H*).

^{31}P{^{1}H} NMR (162 MHz, 298 K, pyridine): δ = –16.56 (s).

Mass spectrometry (APCI): $C_{20}H_{18}BrP$+H ([M+H]$^{+}$); Calcd. = 369.0402, Found = 369.0397.

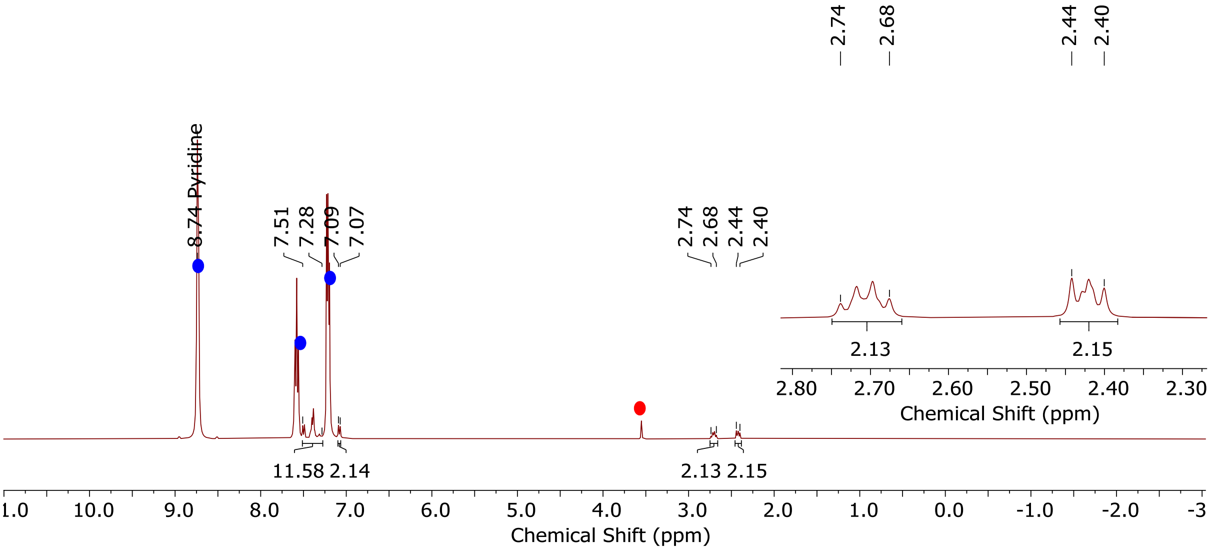

Figure S61. ^{1}H NMR spectrum (pyridine) of crude **6e**. ● = pyridine, ● = 18-c-6.

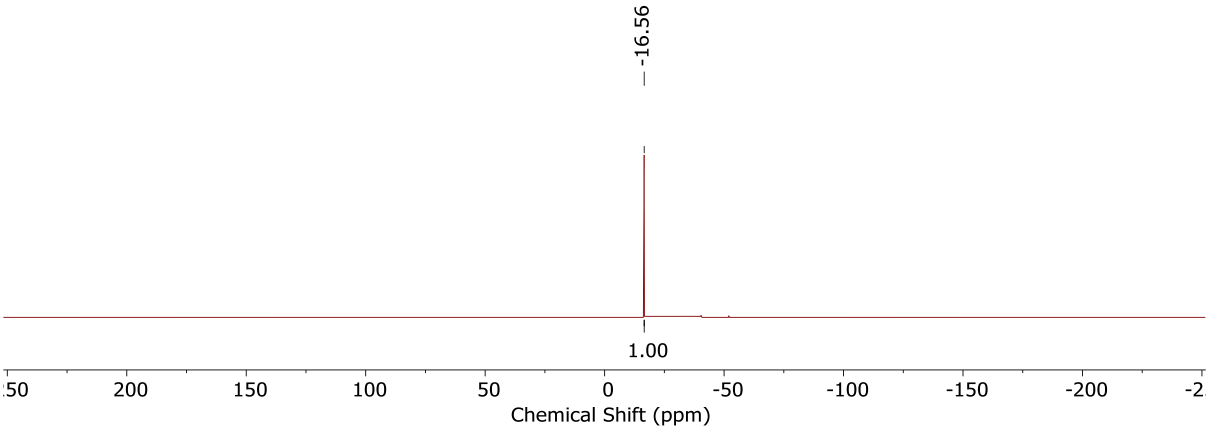

Figure S62. ^{31}P{^{1}H} NMR spectrum (pyridine) of crude **6e**.

3.2.6. **6f** – (2-(naphthalen-2-yl)ethyl)diphenylphosphane

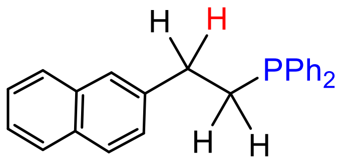

6f

(2-(naphthalen-2-yl)ethyl)diphenylphosphane

Conversion: 9%.

^{1}H NMR (400 MHz, 298 K, pyridine): Due to low conversion, ^{1}H NMR could not be assigned.

^{31}P{^{1}H} NMR (162 MHz, 298 K, pyridine): δ = –17.14 (s).

Mass spectrometry (APCI): $C_{26}H_{23}P$+H ($[M+H]^+$); Calcd. = 367.1610, Found = 367.1599.

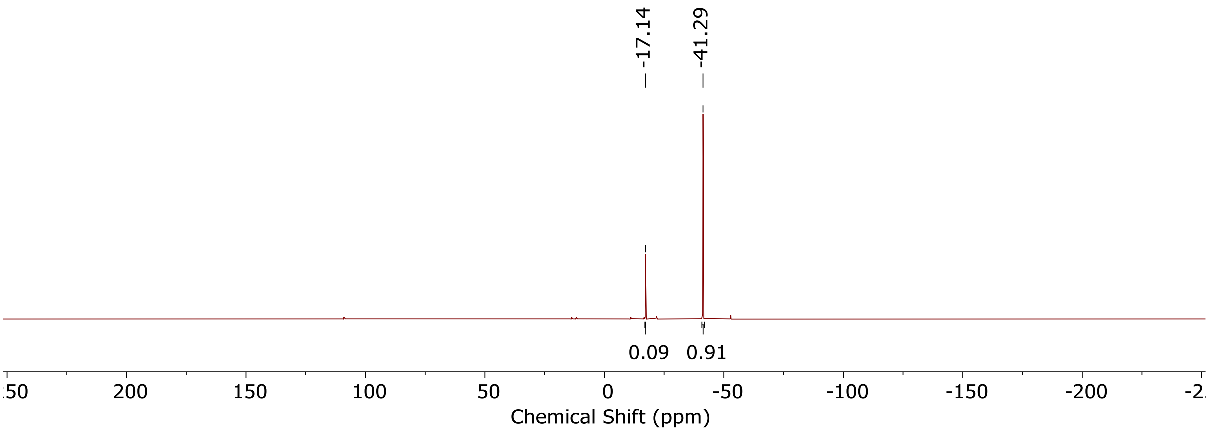

Figure S63. ^{31}P{^{1}H} NMR spectrum (pyridine) of crude **6f**.

3.2.7. **6g** – diphenyl(2-(triethoxysilyl)ethyl)phosphane

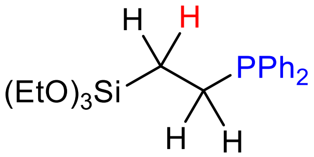

6g

diphenyl(2-(triethoxysilyl)ethyl)phosphane

Conversion: >99%.

^{1}H NMR (400 MHz, 298 K, pyridine): δ = 0.91–0.98 (m, 2H, $CH_2CH_2PPh_2$), 1.23 (t, $^{3}J_{HH}$ = 6.76 Hz, 9H, OCH_2CH_3), 2.38–2.43 (m, 2H, $CH_2CH_2PPh_2$), 3.88 (q, $^{3}J_{HH}$ = 6.76 Hz, 6H, OCH_2CH_3), 7.32–7.40 (m, 6H, Ar–*H*), 7.61–7.64 (m, 4H, Ar–*H*).

^{29}Si{^{1}H} NMR (79 MHz, 298 K, pyridine): δ = –46.89 (d, $^{3}J_{SiP}$ = 28.4 Hz).

^{31}P{^{1}H} NMR (162 MHz, 298 K, pyridine): δ = –9.55 (s).

Mass spectrometry (APCI): $C_{20}H_{29}O_3PSi+H$ ($[M+H]^+$); Calcd. = 377.1696, Found = 377.1689.

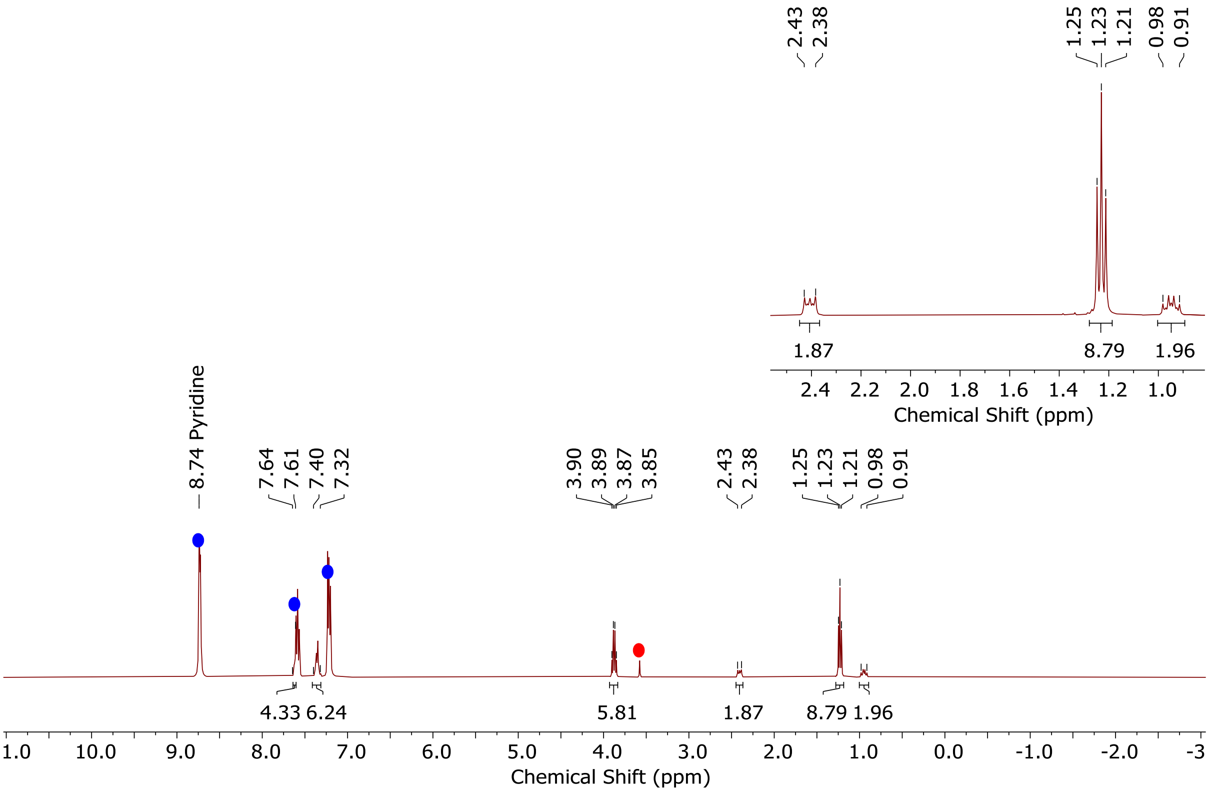

Figure S64. ^{1}H NMR spectrum (pyridine) of crude **6g**. ● = pyridine, ● = 18-c-6.

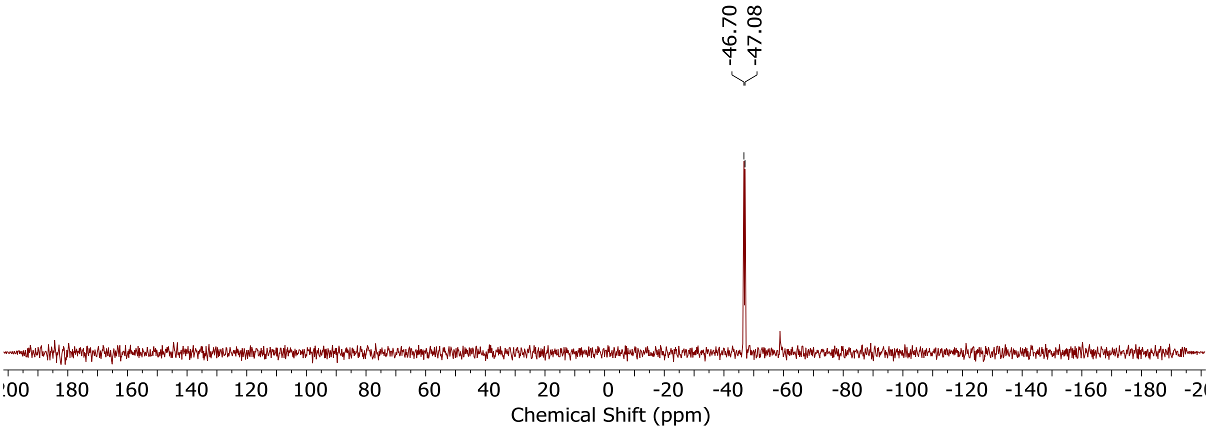

Figure S65. $^{29}Si\{^1H\}$ NMR spectrum (pyridine) of crude **6g**.

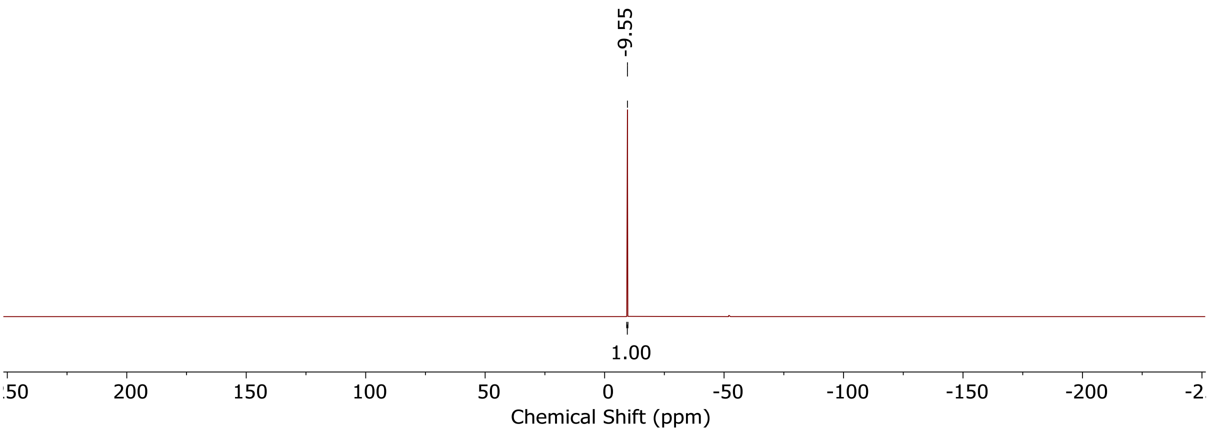

Figure S66. $^{31}P\{^1H\}$ NMR spectrum (pyridine) of crude **6g**.

3.2.8. **6h** – (1,2-diphenylethyl)diphenylphosphane

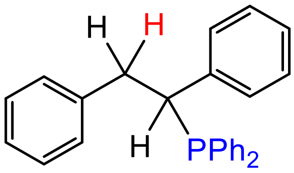

6h

(1,2-diphenylethyl)diphenylphosphane

Conversion: 34%.

^{1}H NMR (400 MHz, 298 K, pyridine): δ = 3.16–3.22 (m, 2H, CH_2), 4.03–4.09 (m, 1H, C*H*), 7.31–7.42 (m, 20H, Ar–*H*).

$^{31}P\{^1H\}$ NMR (162 MHz, 298 K, pyridine): δ = –1.55 (s).

Mass spectrometry (APCI): $C_{26}H_{23}P$+H ($[M+H]^+$); Calcd. = 367.1616, Found = 367.1610.

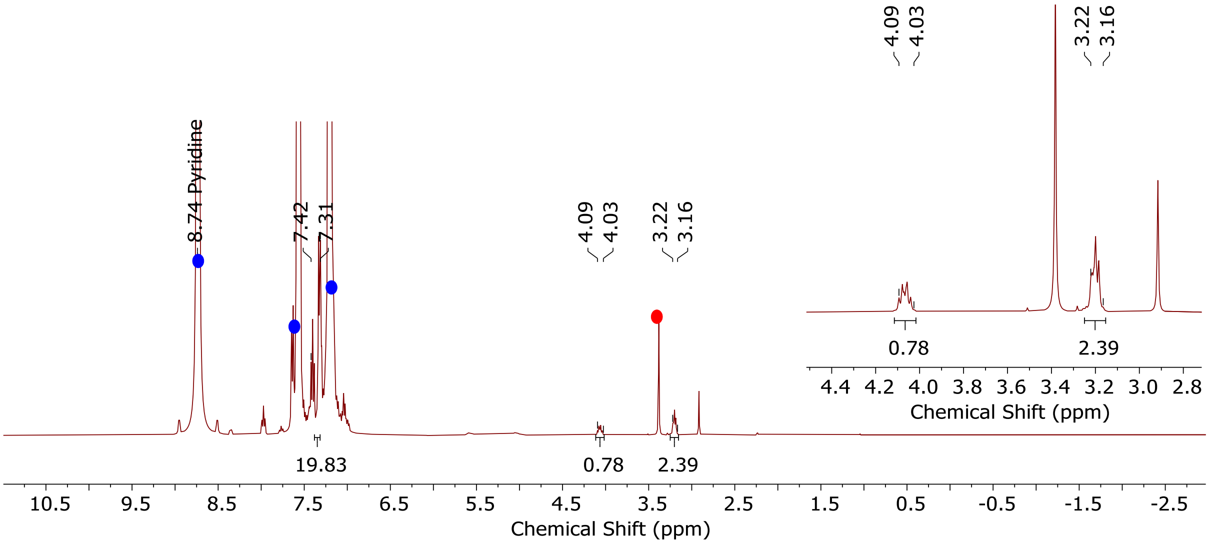

Figure S67. ^{1}H NMR spectrum (pyridine) of crude **6h**. ● = pyridine, ● = 18-c-6.

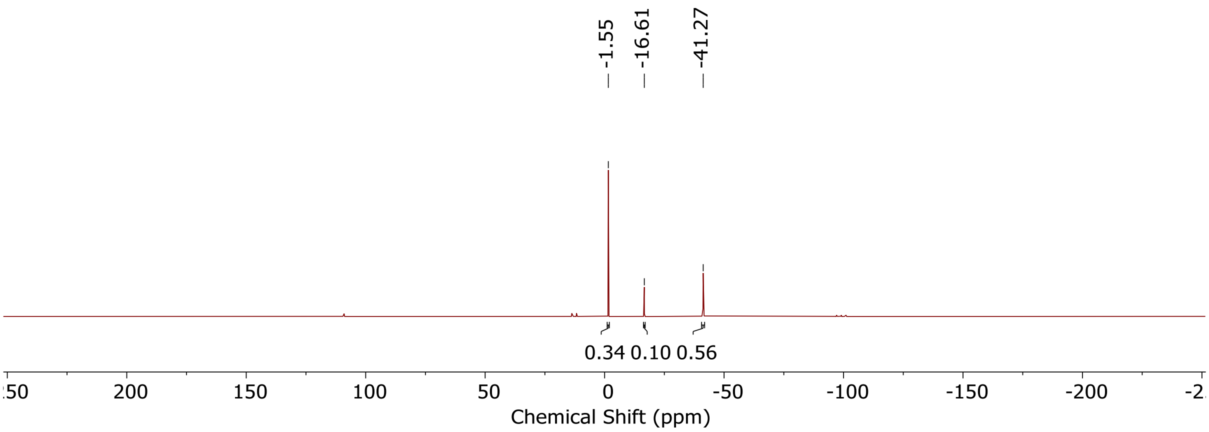

Figure S68. $^{31}P\{^1H\}$ NMR spectrum (pyridine) of crude **6h**. Resonance at –16.61 ppm is attributed to $Ph_2P–PPh_2$.[10]

3.3. Products of Imine Hydrophosphination

3.3.1. **8a** – *N*-((diphenylphosphaneyl)(phenyl)methyl)aniline

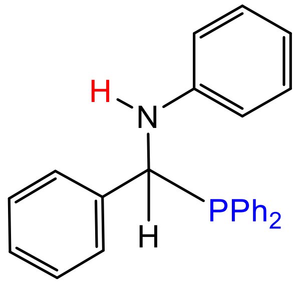

8a

N-((diphenylphosphaneyl)(phenyl)methyl)aniline

Conversion: >99%.

^{1}H NMR (400 MHz, 298 K, pyridine): δ = 5.59 (br, s, 1H, C*H*), 6.76 (t, $^3J_{HH}$ = 7.33 Hz, 1H, Ar–*H*), 6.89 (br, s, 1H, N*H*), 7.04–7.14 (m, 4H, Ar–*H*), 7.26–7.44 (m, 13H, Ar–*H*), 7.75–7.78 (m, 2H, Ar–*H*).

^{31}P{^{1}H} NMR (162 MHz, 298 K, pyridine): δ = 1.47 (s).

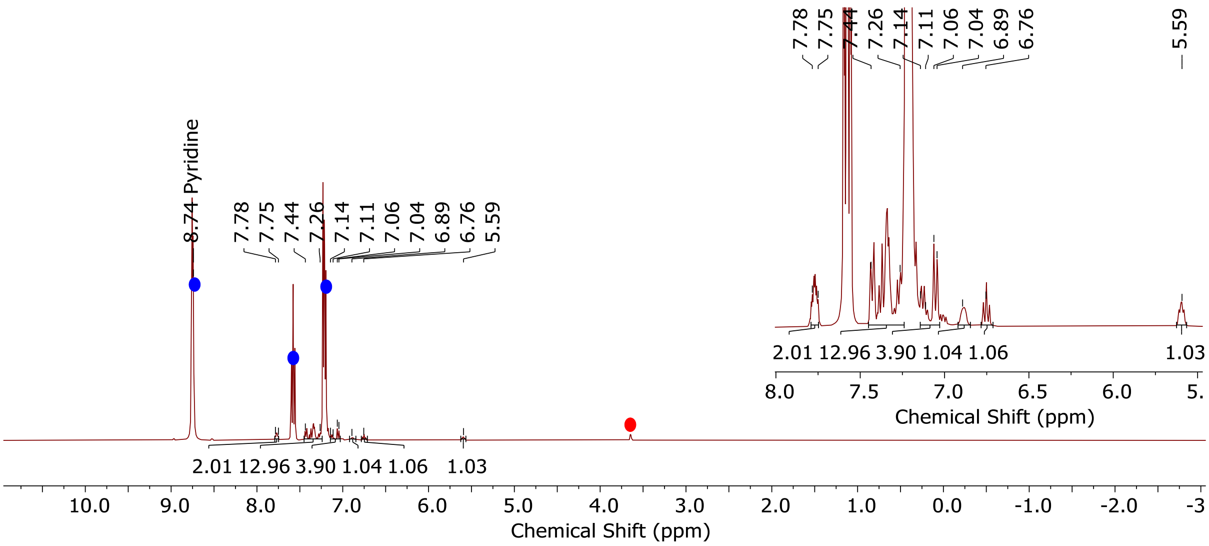

Figure S69. ^{1}H NMR spectrum (pyridine) of crude **8a**. ● = pyridine, ● = 18-c-6.

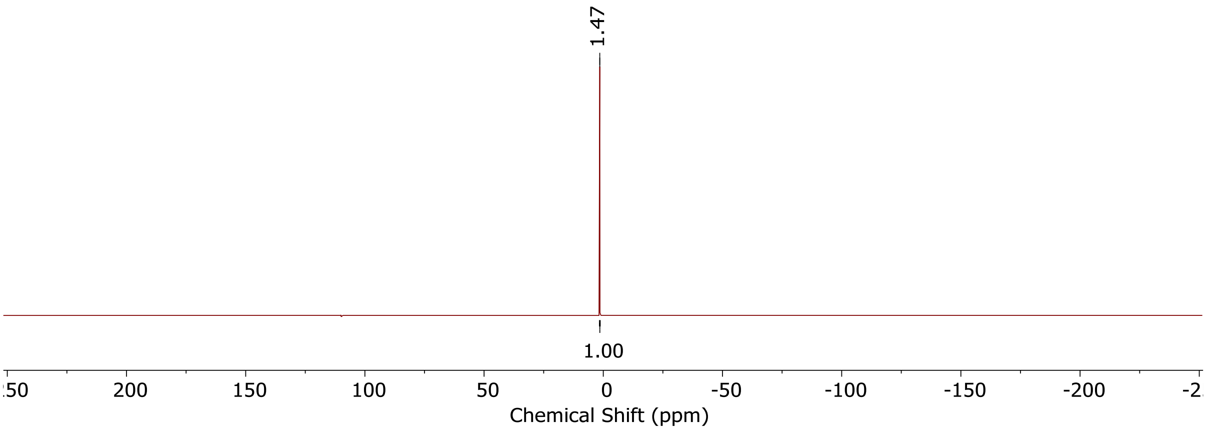

Figure S70. ^{31}P{^{1}H} NMR spectrum (pyridine) of crude **8a**.

3.3.2. **8b** – *N*-((diphenylphosphaneyl)(4-methoxyphenyl)methyl)aniline

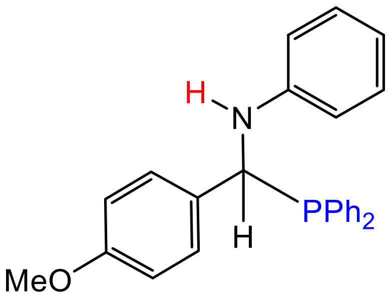

8b

N-((diphenylphosphaneyl)(4-methoxyphenyl)methyl)aniline

Conversion: >99%.

^{1}H NMR (400 MHz, 298 K, pyridine): δ = 3.53 (s, 3H, OCH_3), 5.56 (br, s, 1H, C*H*), 6.73–6.83 (m, 4H, N*H*, Ar–*H*), 7.01–7.07 (m, 2H, Ar–*H*), 7.27–7.43 (m, 12H, Ar–*H*), 7.74–7.80 (m, 2H, Ar–*H*).

^{31}P{^{1}H} NMR (162 MHz, 298 K, pyridine): δ = 0.16 (s).

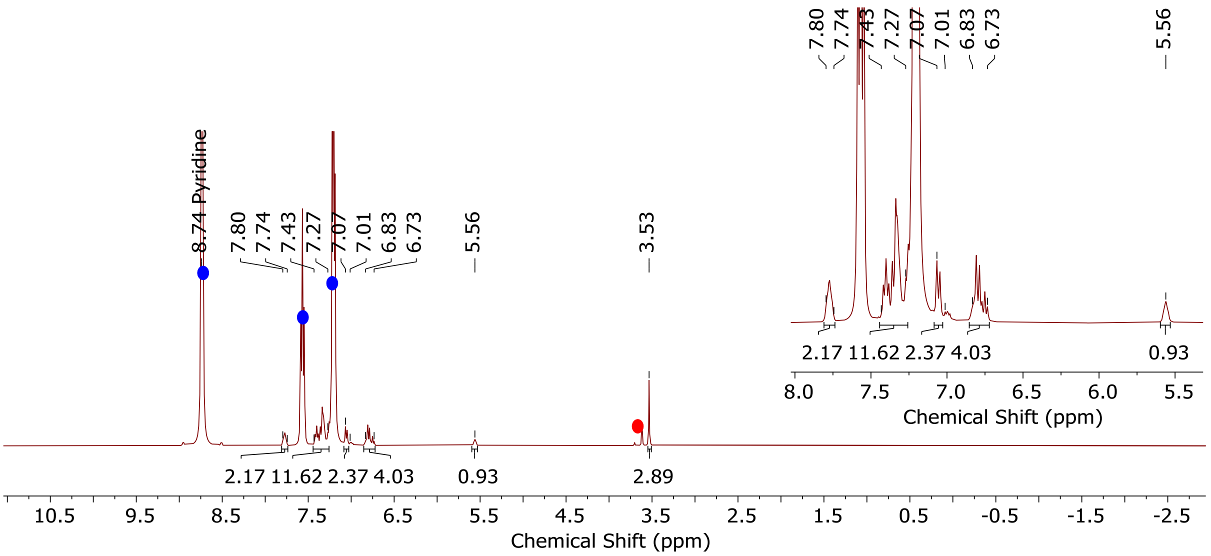

Figure S71. ^{1}H NMR spectrum (pyridine) of crude **8b**. ● = pyridine, ● = 18-c-6.

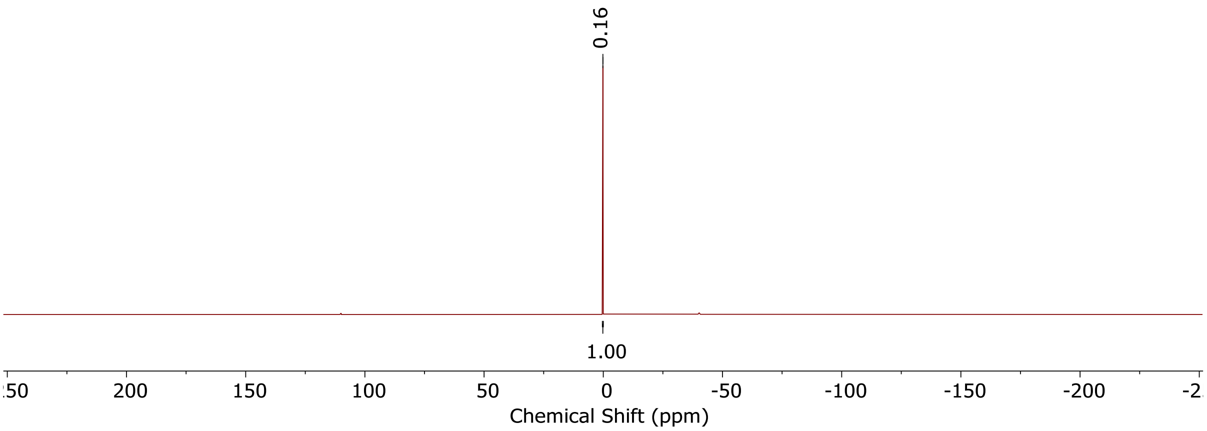

Figure S72. ^{31}P{^{1}H} NMR spectrum (pyridine) of crude **8b**.

3.3.3. **8c** – *N*-((diphenylphosphaneyl)(p-tolyl)methyl)aniline

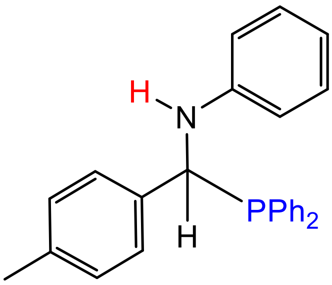

8c

N-((diphenylphosphaneyl)(*p*-tolyl)methyl)aniline

Conversion: >99%.

^{1}H NMR (400 MHz, 298 K, pyridine): δ = 2.10 (s, 3H, C*H*$_3$), 5.57 (br, s, 1H, C*H*), 6.73 (t, $^{3}J_{HH}$ = 7.26 Hz, 1H, Ar–*H*), 6.79 (br, s, 1H, N*H*), 6.96 (d, $^{3}J_{HH}$ = 7.26 Hz, 2H, Ar–*H*), 7.04 (d, $^{3}J_{HH}$ = 7.26 Hz, 2H, Ar–*H*), 7.26–7.42 (m, 12H, Ar–*H*), 7.73–7.78 (m, 2H, Ar–*H*).

$^{31}P\{^1H\}$ NMR (162 MHz, 298 K, pyridine): δ = 0.68 (s).

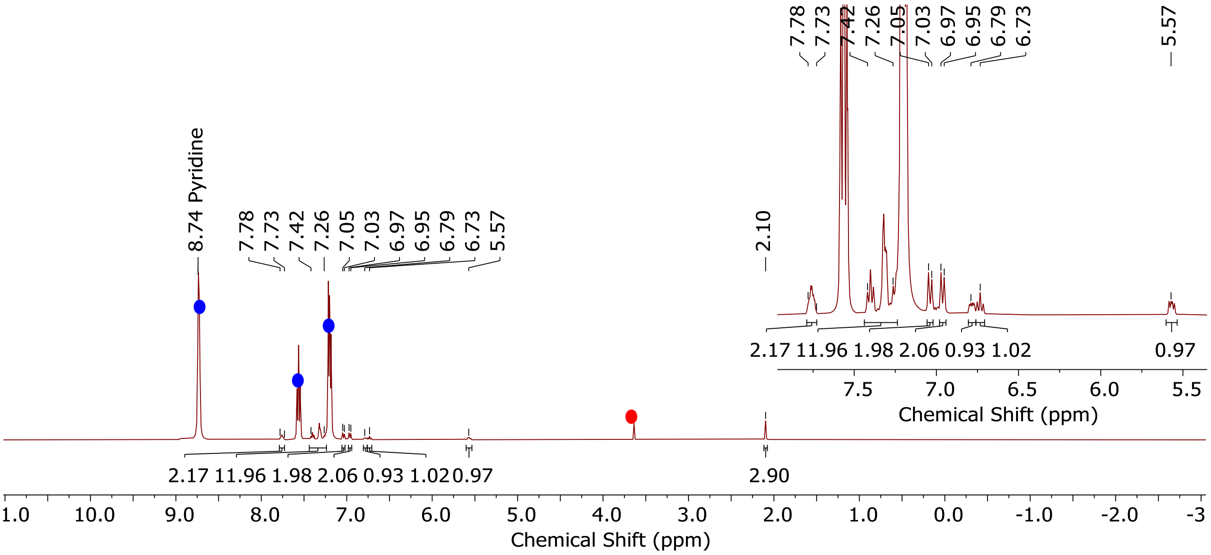

Figure S73. ^{1}H NMR spectrum (pyridine) of crude **8c**. ● = pyridine, ● = 18-c-6.

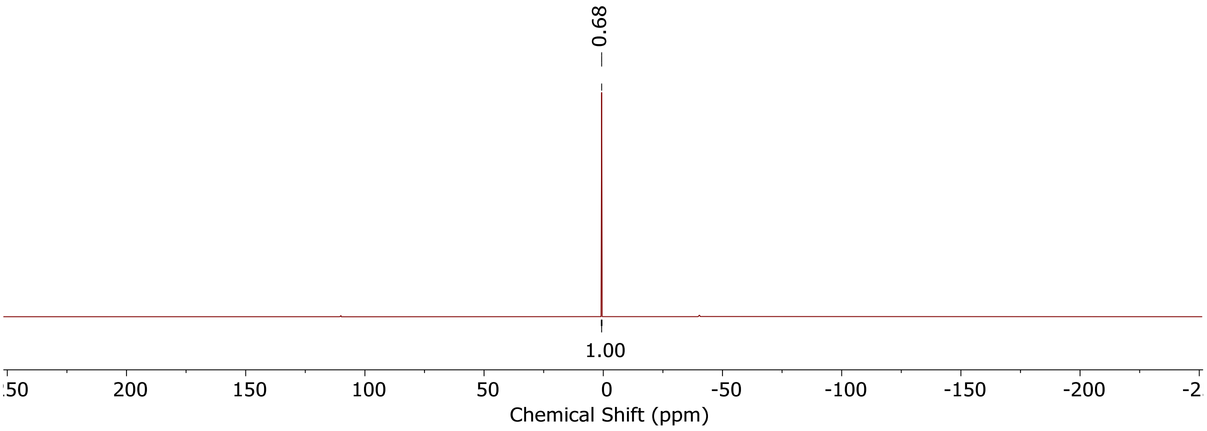

Figure S74. $^{31}P\{^1H\}$ NMR spectrum (pyridine) of crude **8c**.

3.3.4. **8d** – *N*-((4-bromophenyl)(diphenylphosphaneyl)methyl)aniline

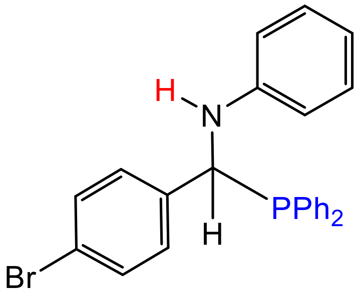

8d

N-((4-bromophenyl)(diphenylphosphaneyl)methyl)aniline

Conversion: 89%.

^{1}H NMR (400 MHz, 298 K, pyridine): δ = 5.50 (br, s, 1H, C*H*), 6.78 (t, $^{3}J_{HH}$ = 7.30 Hz, 1H, Ar–*H*), 6.94 (br, s, 1H, N*H*), 7.04 (d, $^{3}J_{HH}$ = 8.34 Hz, 2H, Ar–*H*), 7.23–7.42 (m, 15H, Ar–*H*), 7.73–7.78 (m, 2H, Ar–*H*).

^{31}P{^{1}H} NMR (162 MHz, 298 K, pyridine): δ = 1.69 (s).

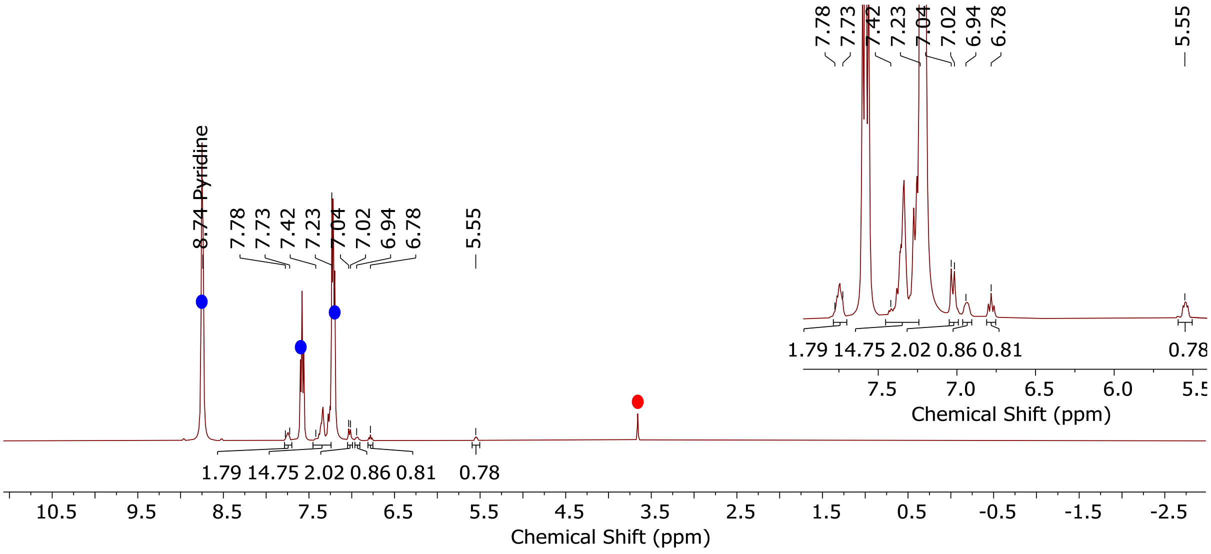

Figure S75. ^{1}H NMR spectrum (pyridine) of crude **8d**. ● = pyridine, ● = 18-c-6.

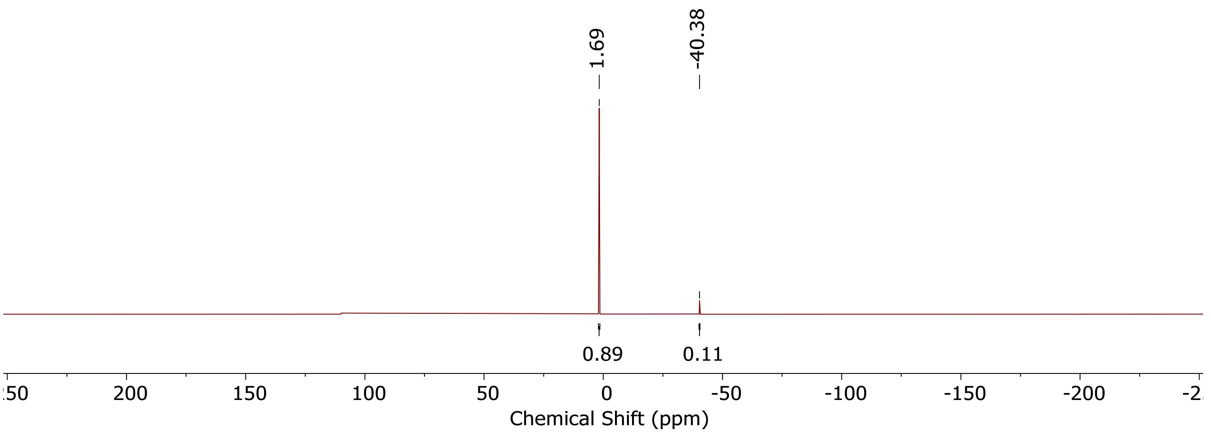

Figure S76. ^{31}P{^{1}H} NMR spectrum (pyridine) of crude **8d**.

4. Preparatory Scale Synthesis

General Procedure; An ampule was charged with initiator **A** (1 mol%) and 18-crown-6 (3 mol%). Substrate was transferred with pyridine (1 mL/ mmol) followed by the addition of an equimolar amount of diphenylphosphine. The reaction was stirred, and an aliquot taken at the indicated time to test for reaction completion (see main paper for reaction times in scope). The solution was filtered and volatiles removed under reduced pressure to yield a crude material. This material was dissolved in DCM (4 mL/

mmol) and filtered through silica. All volatiles were removed under reduced pressure and product isolated.

4.1. Preparatory Scale Synthesis of (*Z*)-4-(2-(diphenylphosphaneyl)vinyl)aniline (4h)

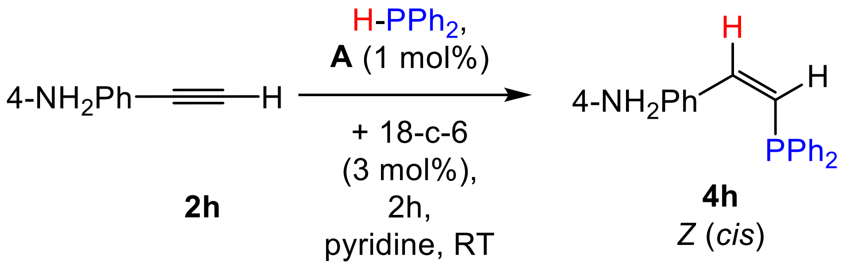

Prepared according to the general procedure with *p*-ethynylaniline (**2h**, 586 mg, 5 mmol), diphenylphosphine (931 mg, 5 mmol), and initiator **A** (17 mg, 0.05 mmol, 1 mol%) and 18-crown-6 (40 mg, 0.15 mmol, 3 mol%). The reaction was stirred for two hours before work-up. (*Z*)-4-(2-(diphenylphosphaneyl)vinyl)aniline (**4h**) was isolated as a pale yellow powder.

Isolated Yield: 1.1848 g, 3.90 mmol, 78%

^{1}H NMR (400 MHz, 298 K, C_6D_6): δ = 2.74 (s, 2H, NH_2), 6.19 (d, $^{3}J_{HH}$ = 8.54 Hz, 2H, *m*-Ar), 6.23 (dd, $^{3}J_{HH}$ = 12.78 Hz, $^{3}J_{HP}$ = 2.70 Hz, 1H, C*H*=CHPPh$_2$), 7.00–7.10 (m, 6H, *m*,*p*-PPh$_2$), 7.19 (dd, $^{2}J_{HP}$ = 24.27 Hz, $^{3}J_{HH}$ = 12.78 Hz, 1H, CH=C*H*PPh$_2$), 7.53–7.56 (m, 6H, *o*-Ar/*o*-PPh$_2$).

^{13}C{^{1}H} NMR (101 MHz, 298 K, C_6D_6): δ = 114.44 (s, *m*-Ar), 124.76 (d, $^{2}J_{CP}$ = 15.86 Hz, *o*-PPh$_2$), 128.52 (s, *i*-Ar), 128.83 (d, $^{4}J_{CP}$ = 6.57 Hz, *p*-PPh$_2$), 131.65 (d, $^{3}J_{CP}$ = 9.79 Hz, *m*-PPh$_2$), 133.18 (d, $^{1}J_{CP}$ = 19.08 Hz, *i*-PPh$_2$), 140.56 (d, $^{2}J_{CP}$ = 10.72 Hz, *C*H=CHPPh$_2$), 144.51 (d, $^{1}J_{CP}$ = 19.08 Hz, CH=*C*HPPh$_2$), 147.37 (s, *p*-Ar).

^{31}P{^{1}H} NMR (162 MHz, 298 K, C_6D_6): δ = –23.33 (s).

Mass spectrometry (APCI): $C_{20}H_{18}$NP+H ([M+H]$^+$); Calcd. = 304.1250, Found = 304.1246.

Elemental Analysis: Expected/ Found: C = 79.19/ 78.24; H = 5.98/5.93; N = 4.62/ 4.70. Despite multiple efforts we could not obtain more accurate elemental analysis in C. See Section 1.2 for further details.

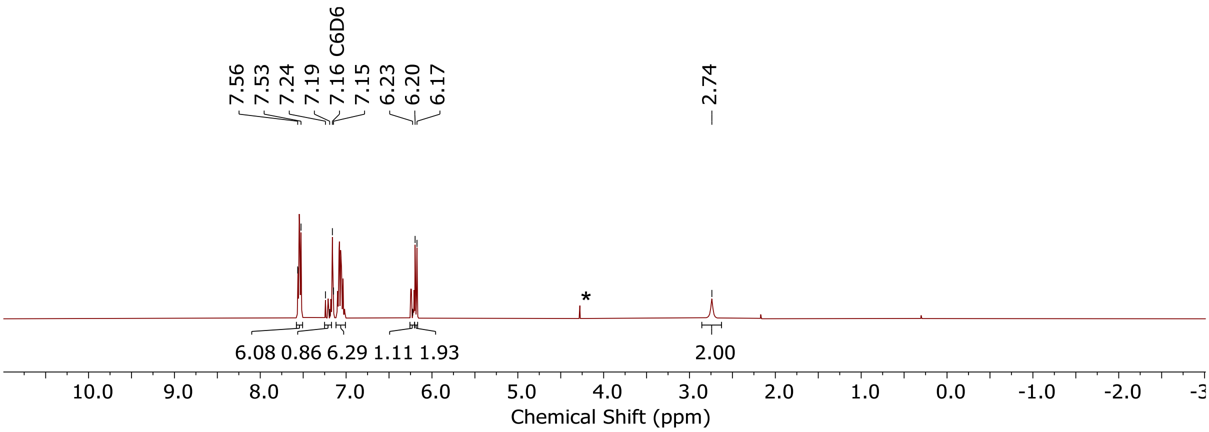

Figure S77. ^{1}H NMR spectrum (C_6D_6) of isolated **4h**. * denotes trace DCM.

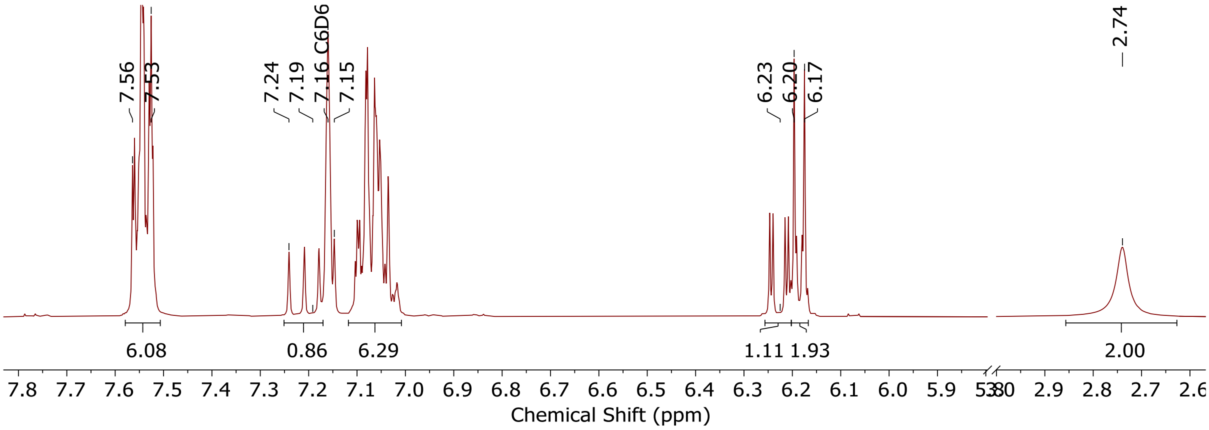

Figure S78. Zoomed in ^{1}H NMR spectrum (C_6D_6) of isolated **4h**. Region of 3.0–5.8 removed for further clarity.

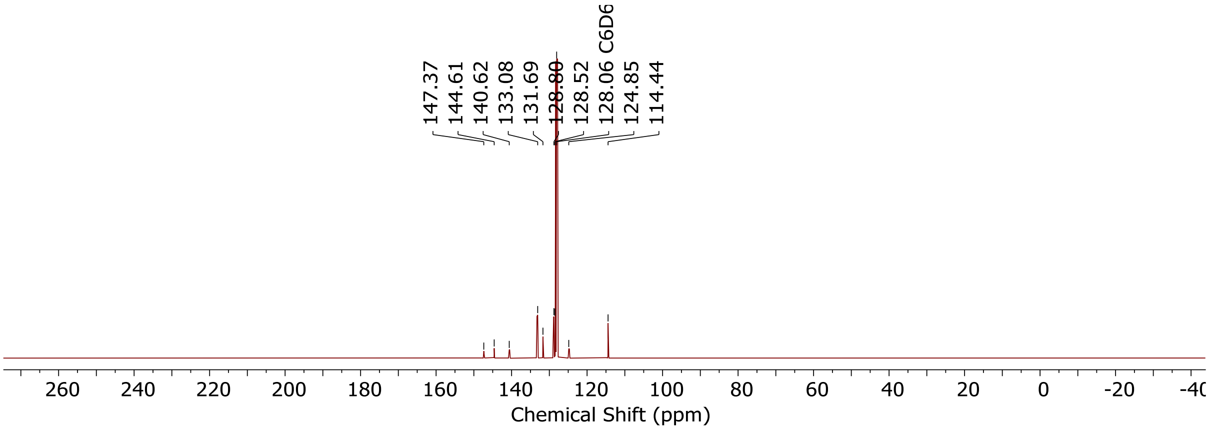

Figure S79. $^{13}C\{^{1}H\}$ NMR spectrum (C_6D_6) of isolated **4h**.

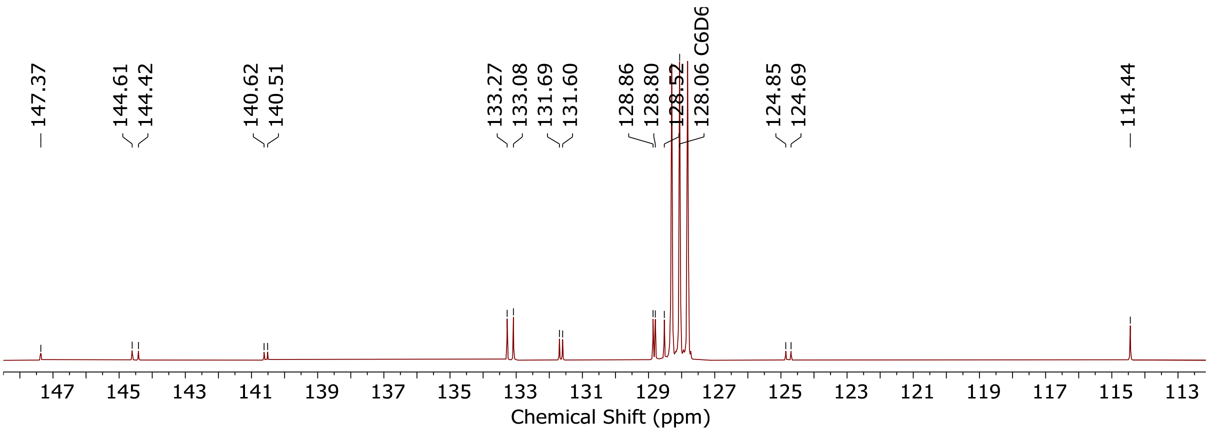

Figure S80. Zoomed in $^{13}C\{^1H\}$ NMR spectrum (C_6D_6) of isolated **4h**.

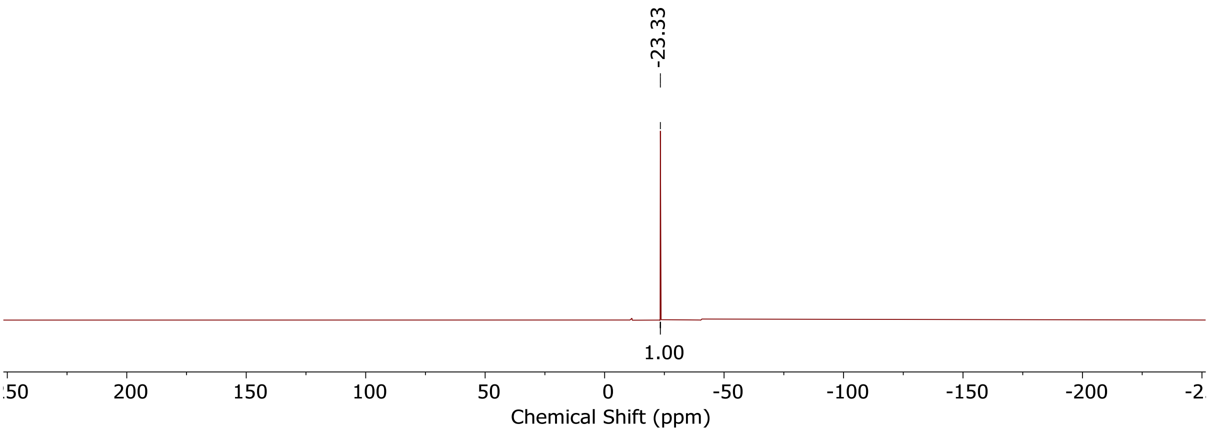

Figure S81. $^{31}P\{^1H\}$ NMR spectrum (C_6D_6) of isolated **4h**.

4.2. Preparatory Scale Synthesis of phenethyldiphenylphosphane (6a)

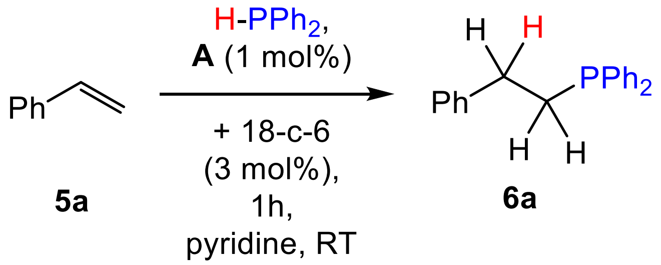

Prepared according to the general procedure with styrene (**5a**, 521 mg, 5 mmol), diphenylphosphine (931 mg, 5 mmol), and initiator **A** (17 mg, 0.05 mmol, 1 mol%) and 18-crown-6 (40 mg, 0.15 mmol, 3 mol%). The reaction was stirred for one hour before work-up. Phenethyldiphenylphosphane (**6a**) was isolated as a colorless oil.

Isolated Yield: 1.1414 g, 3.92 mmol, 78%

^{1}H NMR (400 MHz, 298 K, C_6D_6): δ = 2.22–2.26 (m, 2H, $CH_2CH_2PPh_2$), 2.65–2.72 (m, 2H, $CH_2CH_2PPh_2$), 6.96–6.99 (m, 2H, *o*-Ar), 7.05–7.14 (m, 9H, *m*-/*p*-PPh_2, *m*-/*p*-Ar), 7.39–7.44 (m, 4H, o-PPh_2).

$^{13}C\{^1H\}$ NMR (101 MHz, 298 K, C_6D_6): δ = 30.84 (d, $^2J_{CP}$ = 14.32 Hz, $CH_2CH_2PPh_2$), 32.66 (d, $^1J_{CP}$ = 18.51 Hz, $CH_2CH_2PPh_2$), 126.25 (s, *p*-PPh_2), 128.56 (s, Ar), 128.73 (s, Ar), 128.74 (s, Ar), 128.75 (d, $^3J_{CP}$ = 9.70 Hz, *m*-PPh_2), 133.18 (d, $^2J_{CP}$ = 18.46 Hz, *o*-PPh_2), 139.53 (d, $^1J_{CP}$ = 14.32 Hz, *i*-PPh_2), 142.99 (d, $^1J_{CP}$ = 14.32 Hz, *i*-PPh_2).

$^{31}P\{^1H\}$ NMR (162 MHz, 298 K, C_6D_6): δ = –16.04 (s).

Mass spectrometry (APCI): $C_{20}H_{19}P$+H ($[M+H]^+$); Calcd. = 291.1297, Found = 291.1295.

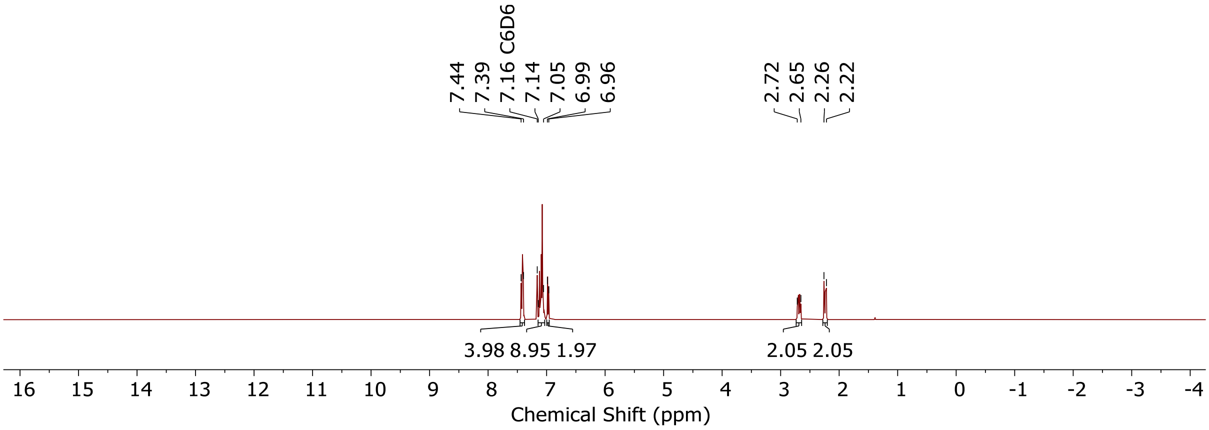

Figure S82. ^1H NMR spectrum (C_6D_6) of isolated **6a**.

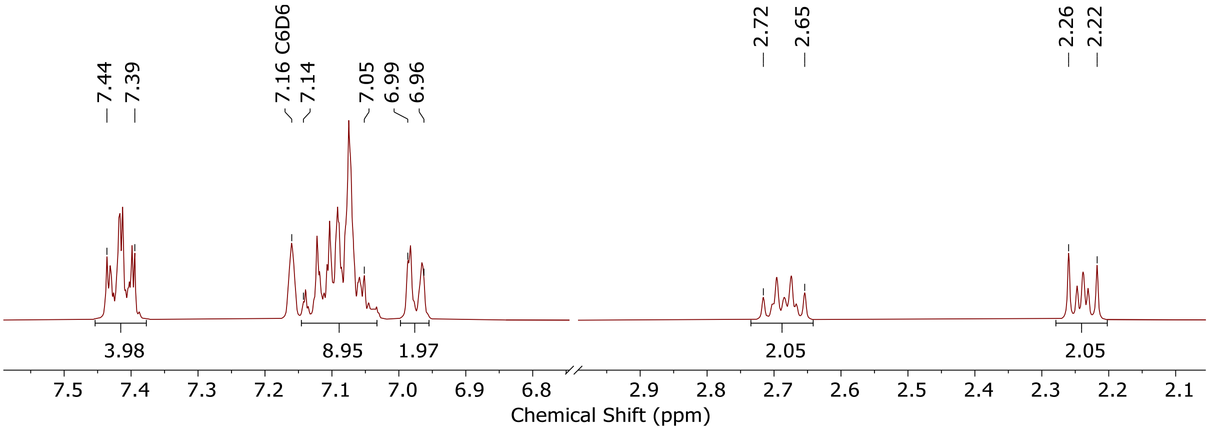

Figure S83. Zoomed in ^1H NMR spectrum (C_6D_6) of isolated **6a**. Region of 3.0–6.75 removed for further clarity.

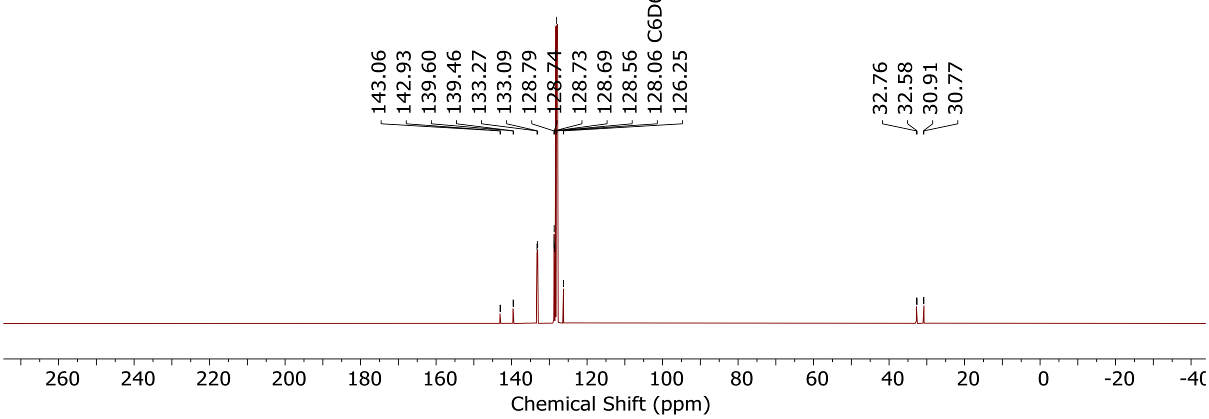

Figure S84. $^{13}C\{^{1}H\}$ NMR spectrum (C_6D_6) of isolated **6a**.

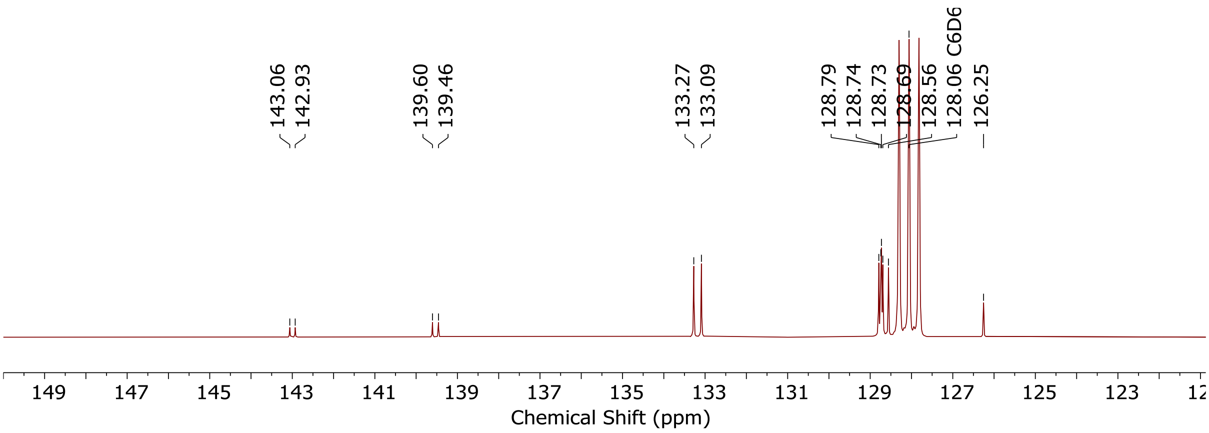

Figure S85. Zoomed in $^{13}C\{^{1}H\}$ NMR spectrum (C_6D_6) of isolated **6a**.

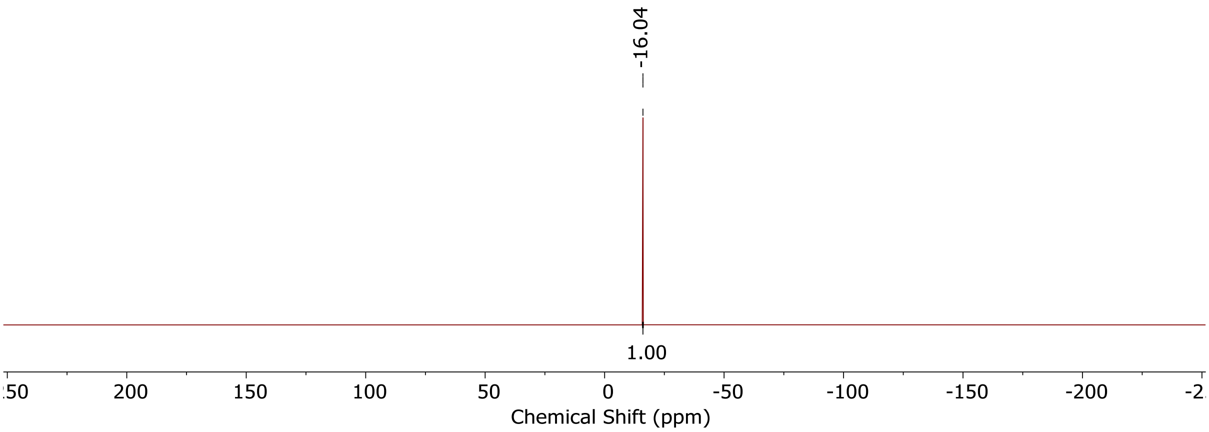

Figure S86. $^{31}P\{^{1}H\}$ NMR spectrum (C_6D_6) of isolated **6a**.

4.3. Preparatory Scale Synthesis of (4-methoxyphenethyl)diphenylphosphane (6b)

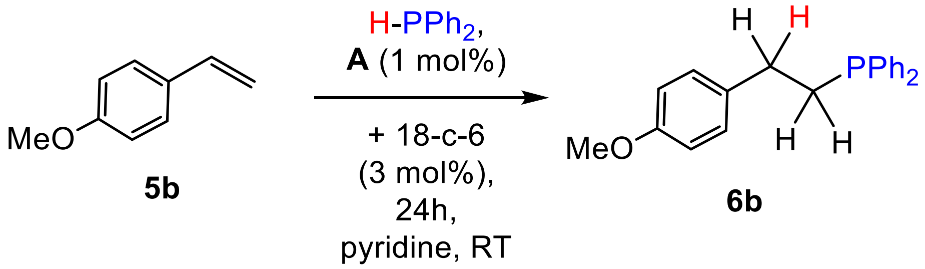

Prepared according to the general procedure with 4-methoxystyrene (**5b**, 317 mg, 2.4 mmol), diphenylphosphine (440 mg, 2.4 mmol), and initiator **A** (8 mg, 0.024 mmol, 1 mol%) and 18-crown-6 (19.5 mg, 0.072 mmol, 3 mol%). The reaction was stirred for 24 hours before work-up. (4-methoxyphenethyl)diphenylphosphane (**6b**) was isolated as a colorless oil.

Isolated Yield: 0.5475 g,1.71 mmol, 71%

^{1}H NMR (400 MHz, 298 K, C_6D_6): δ = 2.25–2.29 (m, 2H, $CH_2CH_2PPh_2$), 2.66–2.72 (m, 2H, $CH_2CH_2PPh_2$), 3.34 (s, 3H, OCH_3), 6.77 (d, $^{3}J_{HH}$ = 8.63 Hz, 2H, *o*-Ar), 6.92 (d, $^{3}J_{HH}$ = 8.63 Hz, 2H, *m*-Ar), 7.06–7.13 (m, 6H, *m*-/*p*-PPh_2), 7.42–7.46 (m, 4H, *o*-PPh_2).

$^{13}C\{^{1}H\}$ NMR (101 MHz, 298 K, C_6D_6): δ = 31.13 (d, $^{2}J_{CP}$ = 14.07 Hz, $CH_2CH_2PPh_2$), 31.82 (d, $^{1}J_{CP}$ = 18.21 Hz, $CH_2CH_2PPh_2$), 54.83 (s, OCH_3), 114.23 (s, Ar), 128.73 (s, Ar), 128.80 (s, Ar), 128.48 (s, Ar), 133.19 (d, $^{2}J_{CP}$ = 18.53 Hz, *o*-PPh_2), 134.92 (d, $^{1}J_{CP}$ = 13.06 Hz, *i*-PPh_2), 139.63 (d, $^{1}J_{CP}$ = 14.74 Hz, *i*-PPh_2), 158.62 (s, Ar).

$^{31}P\{^{1}H\}$ NMR (162 MHz, 298 K, C_6D_6): δ = –16.34 (s).

Mass spectrometry (APCI): $C_{21}H_{21}OP$+H ($[M+H]^+$); Calcd. = 321.1394, Found = 321.1406.

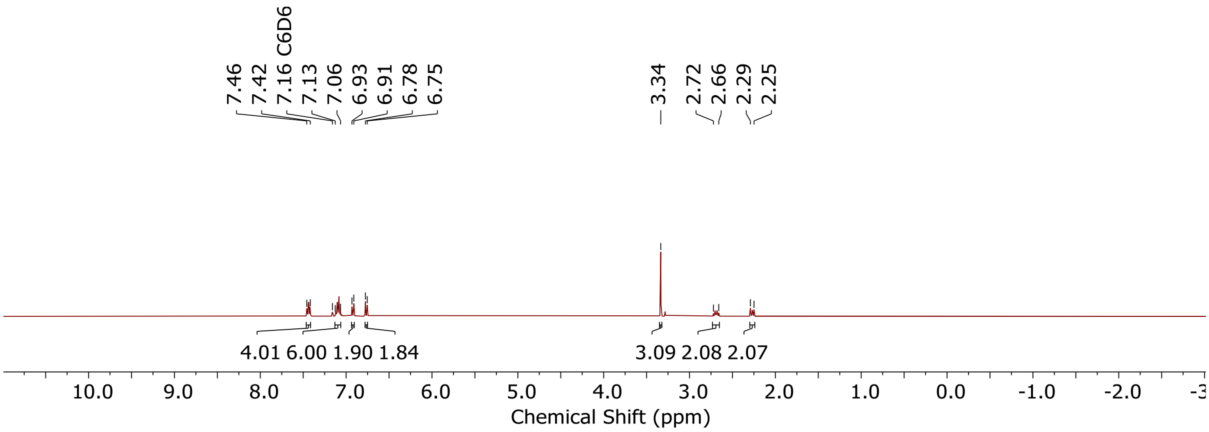

Figure S87. ^{1}H NMR spectrum (C_6D_6) of isolated **6b**.

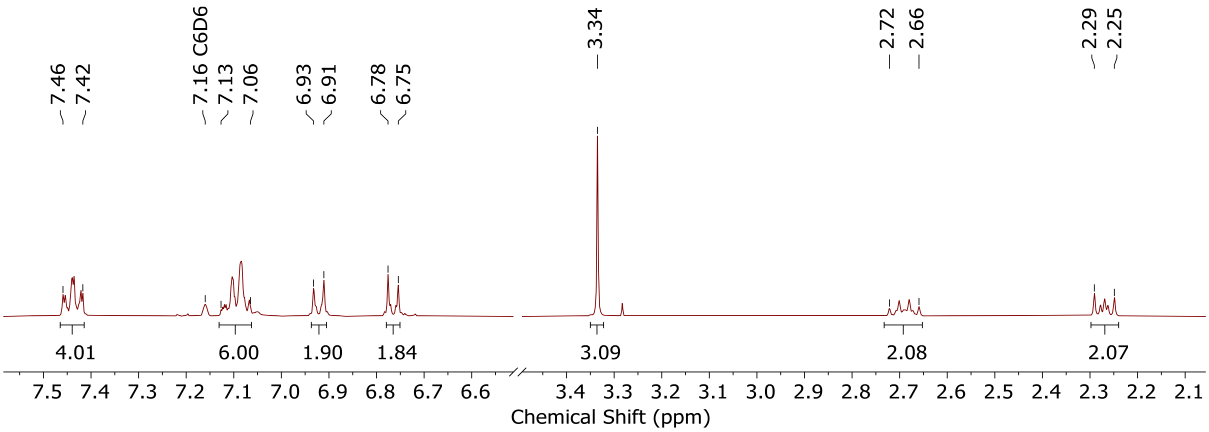

Figure S88. Zoomed in ^{1}H NMR spectrum (C_6D_6) of isolated **6b**. Region of 3.5–6.5 ppm removed for further clarity.

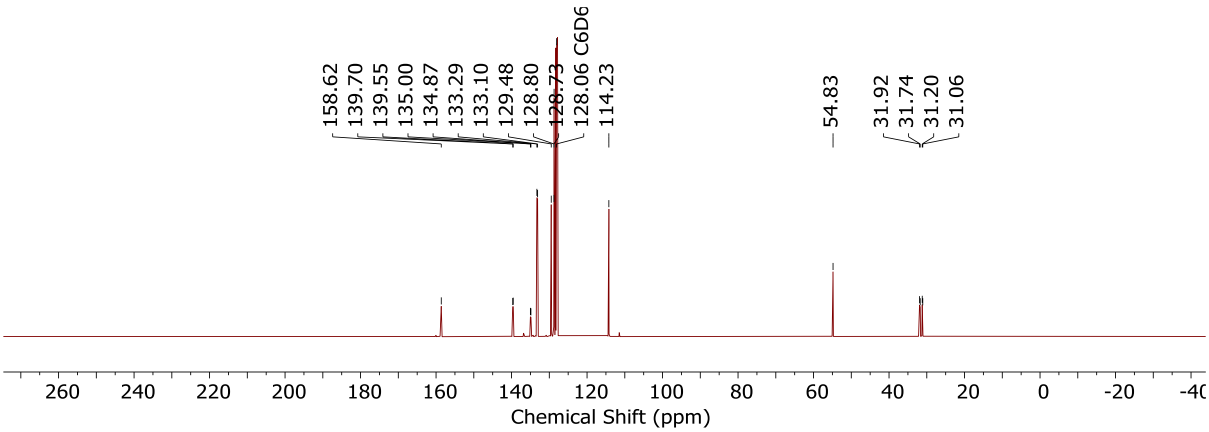

Figure S89. $^{13}C\{^{1}H\}$ NMR spectrum (C_6D_6) of isolated **6b**.

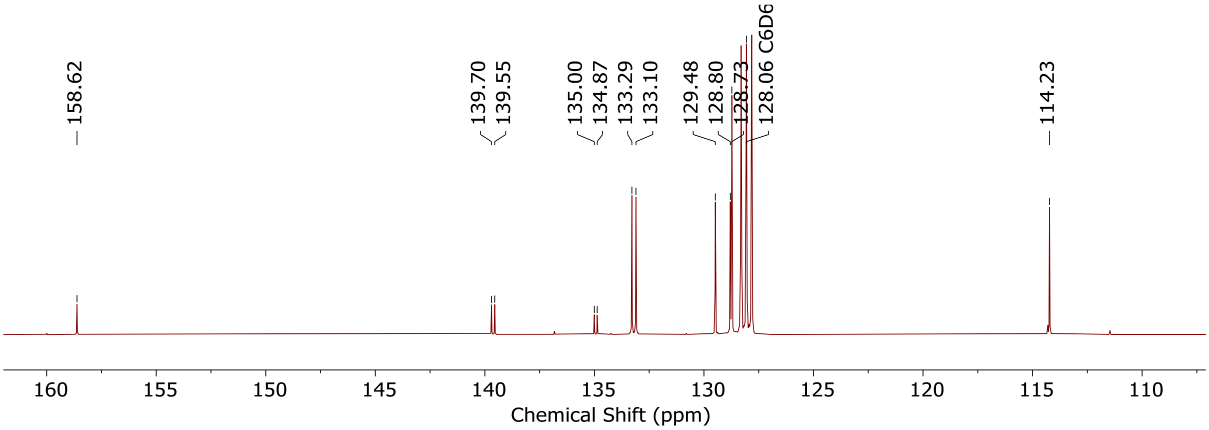

Figure S90. Zoomed in $^{13}C\{^{1}H\}$ NMR spectrum (C_6D_6) of isolated **6b**.

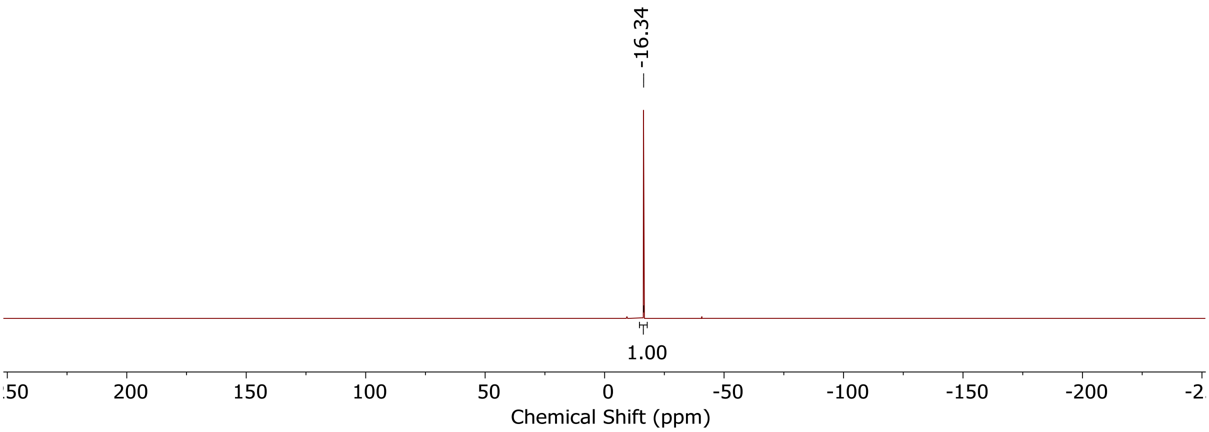

Figure S91. $^{31}P\{^{1}H\}$ NMR spectrum (C_6D_6) of isolated **6b**.

4.4. Preparatory Scale Synthesis of (4-methylphenethyl)diphenylphosphane (6c)

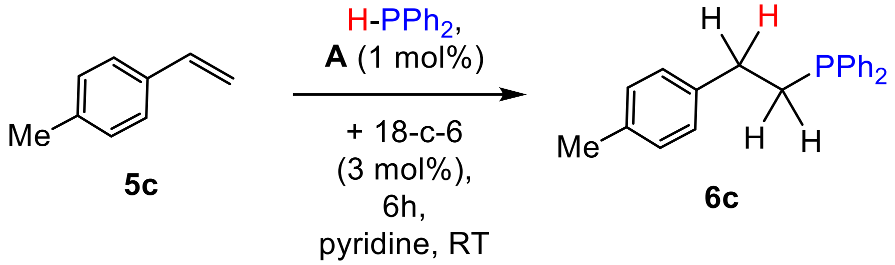

Prepared according to the general procedure with 4-methylstyrene (**5c**, 186 mg, 1.6 mmol), diphenylphosphine (297 mg, 1.6 mmol), and initiator **A** (5.3 mg, 0.016 mmol, 1 mol%) and 18-crown-6 (13 mg, 0.048 mmol, 3 mol%). The reaction was stirred for six hours before work-up. (4-methylphenethyl)diphenylphosphane (**6c**) was isolated as a colorless oil.

Isolated Yield: 0.2441 g, 0.80 mmol, 50%

^{1}H NMR (400 MHz, 298 K, C_6D_6): δ = 2.14 (s, 3H, C*H*$_3$), 2.26–2.30 (m, 2H, C*H*$_2$CH$_2$PPh$_2$), 2.67–2.74 (m, 2H, CH$_2$C*H*$_2$PPh$_2$), 6.93–6.98 (m, 4H, *o*-/*m*-Ar), 7.05–7.14 (m, 6H, *m*-/*p*-PPh$_2$), 7.41–7.45 (m, 4H, o-PPh$_2$).

^{13}C{^{1}H} NMR (101 MHz, 298 K, C_6D_6): δ = 21.09 (s, *C*H$_3$), 30.97 (d, $^2J_{CP}$ = 12.42 Hz, *C*H$_2$CH$_2$PPh$_2$), 32.28 (d, $^1J_{CP}$ = 18.96 Hz, CH$_2$*C*H$_2$PPh$_2$), 128.51 (s, Ar), 128.72 (s, Ar), 128.79 (s, Ar), 129.39 (s, Ar), 133.19 (d, $^2J_{CP}$ = 18.81 Hz, *o*-PPh$_2$), 135.42 (s, Ar), 139.60 (d, $^1J_{CP}$ = 14.96 Hz, *i*-PPh$_2$), 140.00 (d, $^1J_{CP}$ = 13.68 Hz, *i*-PPh$_2$).

^{31}P{^{1}H} NMR (162 MHz, 298 K, C_6D_6): δ = –16.12 (s).

Mass spectrometry (APCI): $C_{21}H_{21}$P+H ([M+H]$^+$); Calcd. = 305.1454, Found = 305.1458.

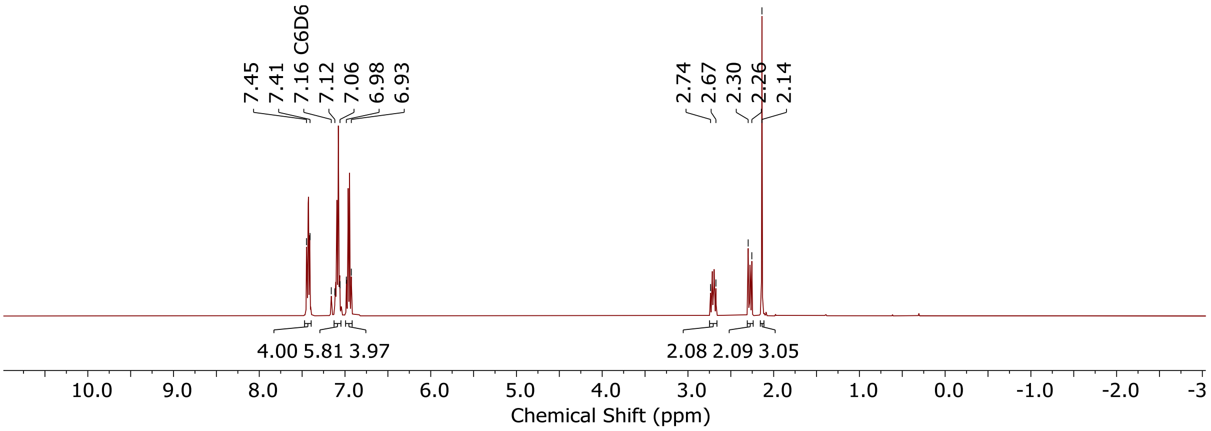

Figure S92. ^{1}H NMR spectrum (C_6D_6) of isolated **6c**.

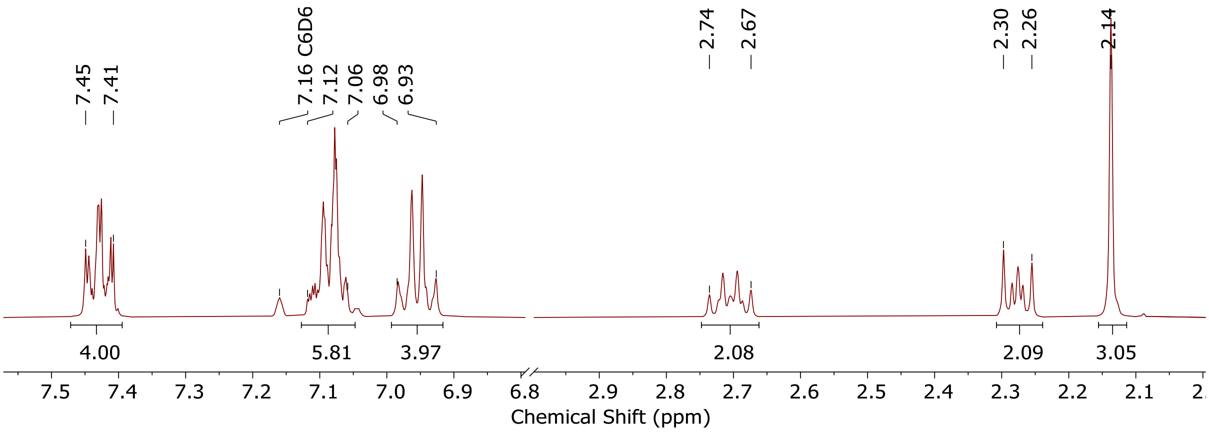

Figure S93. Zoomed in ^{1}H NMR spectrum (C_6D_6) of isolated **6c**. Region of 3.0–6.8 ppm removed for further clarity.

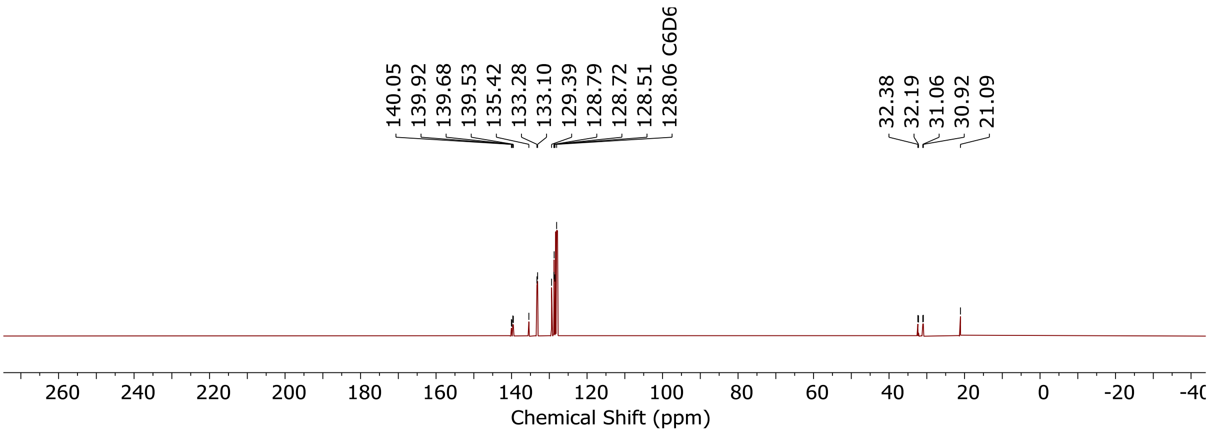

Figure S94. $^{13}C\{^{1}H\}$ NMR spectrum (C_6D_6) of isolated **6c**.

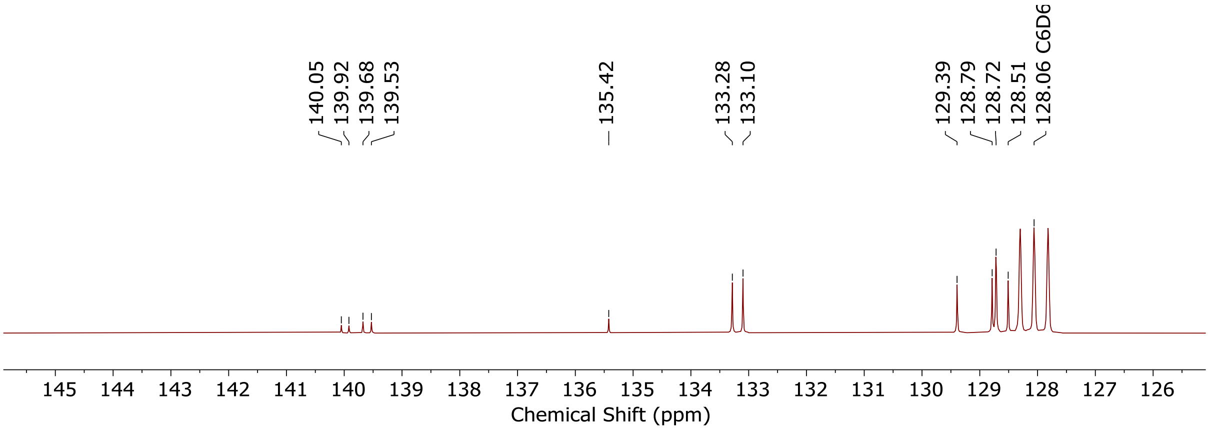

Figure S95. Zoomed in $^{13}C\{^{1}H\}$ NMR spectrum (C_6D_6) of isolated **6c**.

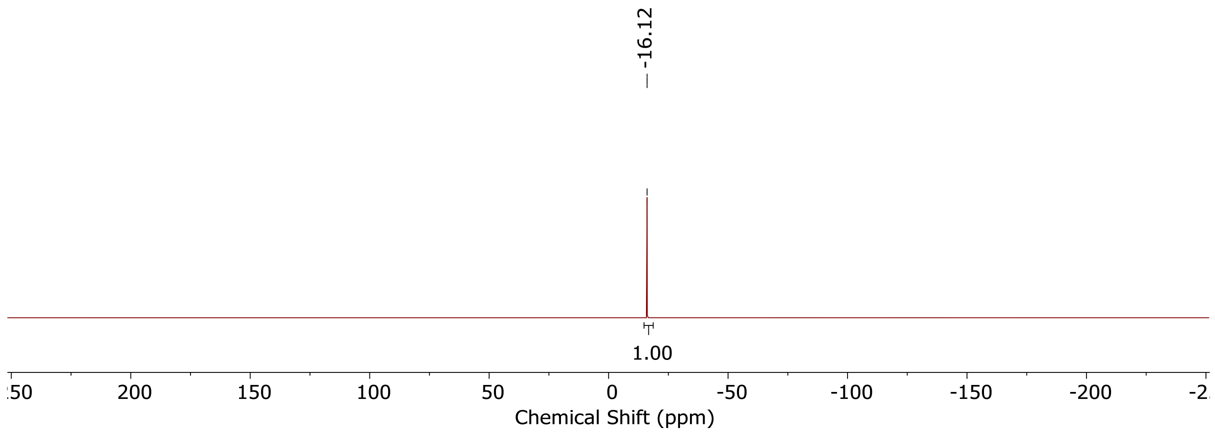

Figure S96. $^{31}P\{^1H\}$ NMR spectrum (C_6D_6) of isolated **6c**.

4.5. Preparatory Scale Synthesis of (4-fluorophenethyl)diphenylphosphane (6d)

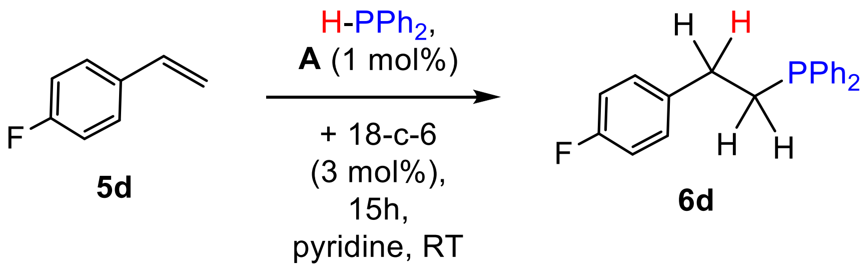

Prepared according to the general procedure with 4-fluorostyrene (**5d**, 611 mg, 5 mmol), diphenylphosphine (931 mg, 5 mmol), and initiator **A** (17 mg, 0.05 mmol, 1 mol%) and 18-crown-6 (40 mg, 0.15 mmol, 3 mol%). The reaction was stirred for 15 hours before work-up. (4-fluorophenethyl)diphenylphosphane (**6d**) was isolated as a colorless oil.

Isolated Yield: 1.2428 g, 4.03 mmol, 81%

^{1}H NMR (400 MHz, 298 K, C_6D_6): δ = 2.11–2.16 (m, 2H, CH_2CH$_2$PPh$_2$), 2.52–2.58 (m, 2H, CH$_2$CH_2PPh$_2$), 6.68–6.71 (m, 2H, *o*-Ar), 6.74–6.79 (m, 2H, *m*-Ar), 7.06–7.12 (m, 6H, *m*-/*p*-PPh$_2$), 7.38–7.43 (m, 4H, o-PPh$_2$).

$^{13}C\{^1H\}$ NMR (101 MHz, 298 K, C_6D_6): δ = 30.75 (d, $^2J_{CP}$ = 15.96 Hz, *C*H$_2$CH$_2$PPh$_2$), 31.74 (d, $^1J_{CP}$ = 18.09 Hz, CH$_2$*C*H$_2$PPh$_2$), 115.31 (d, $^1J_{CF}$ = 21.11 Hz, Ar–*C*F), 128.76 (s, Ar), 128.83 (s, Ar), 129.92 (d, $^3J_{CP}$ = 7.98 Hz, *m*-PPh$_2$), 133.15 (d, $^2J_{CP}$ = 18.05 Hz, *o*-PPh$_2$), 138.50 (d, $^1J_{CP}$ = 14.72 Hz, *i*-PPh$_2$), 139.39 (d, $^1J_{CP}$ = 14.72 Hz, *i*-PPh$_2$), 160.63 (s, Ar), 163.05 (s, Ar).

$^{19}F\{^1H\}$ NMR (376 MHz, 298 K, C_6D_6): δ = –117.23 (s).

$^{31}P\{^1H\}$ NMR (162 MHz, 298 K, C_6D_6): δ = –16.30 (s).

Mass spectrometry (APCI): $C_{20}H_{18}FP+H$ ($[M+H]^+$); Calcd. = 309.1203, Found = 309.1207.

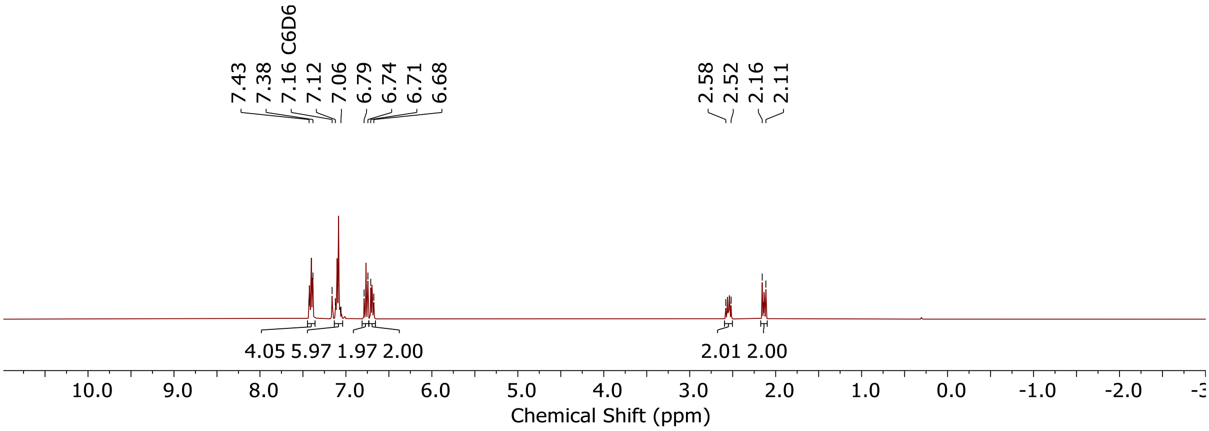

Figure S97. ^1H NMR spectrum (C_6D_6) of isolated **6d**.

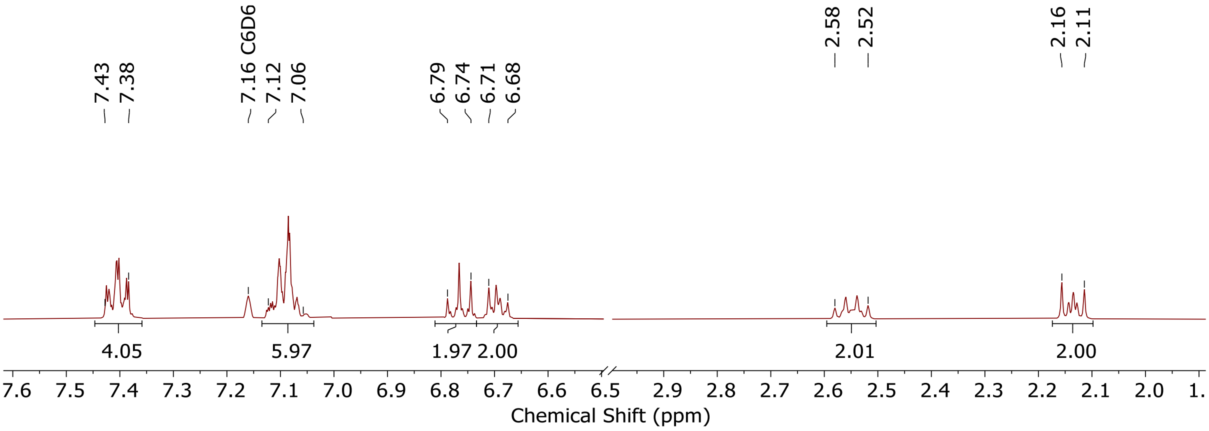

Figure S98. Zoomed in ^1H NMR spectrum (C_6D_6) of isolated **6d**. Region of 3.0–6.5 ppm removed for further clarity.

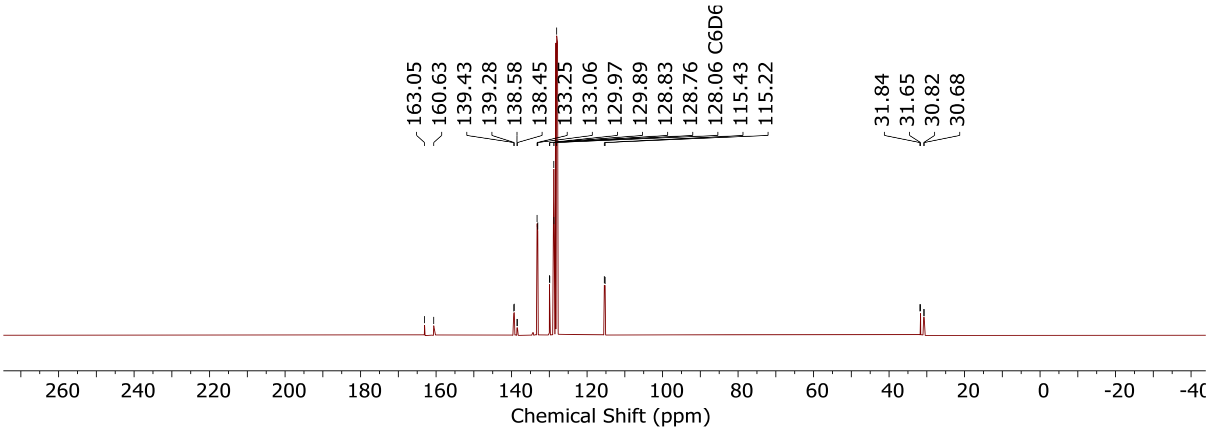

Figure S99. $^{13}C\{^1H\}$ NMR spectrum (C_6D_6) of isolated **6d**.

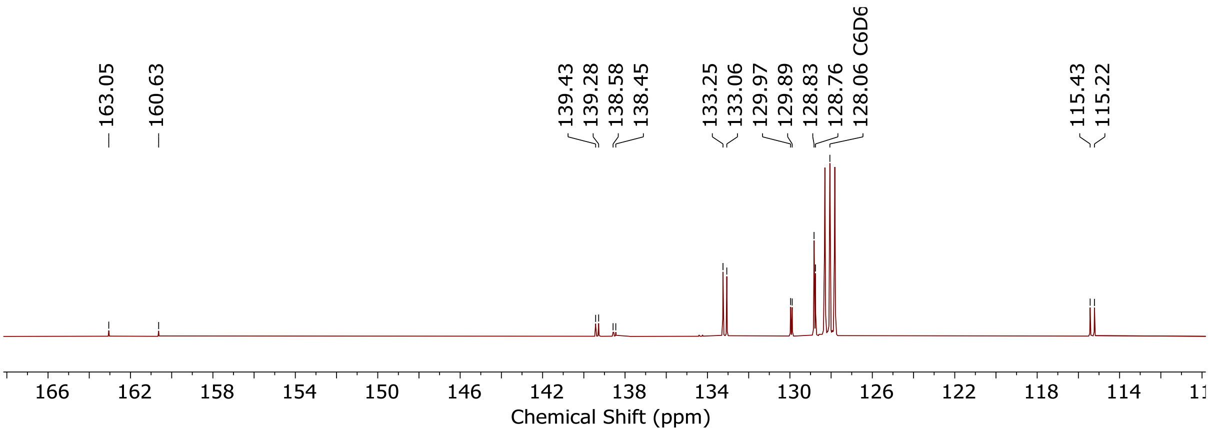

Figure S100. Zoomed in $^{13}C\{^{1}H\}$ NMR spectrum (C_6D_6) of isolated **6d**.

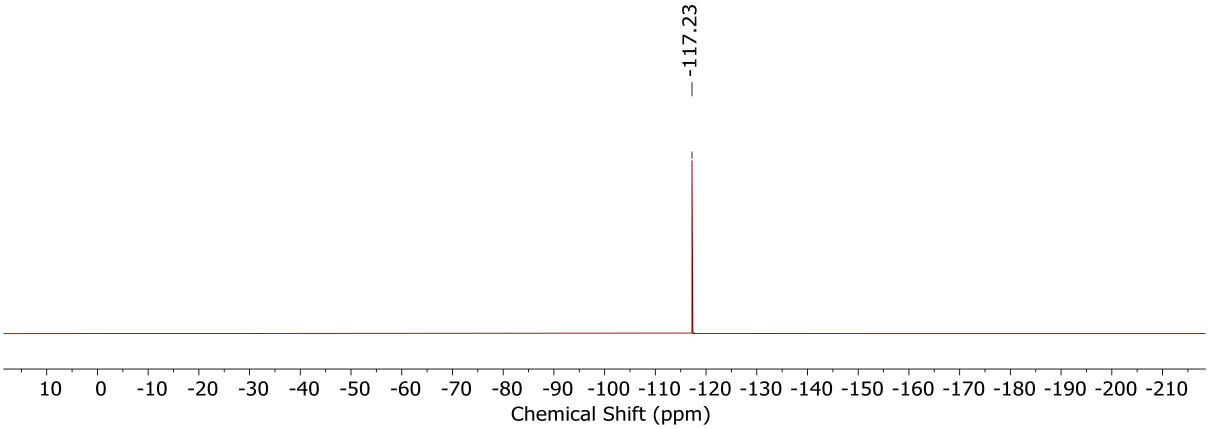

Figure S101. $^{19}F\{^{1}H\}$ NMR spectrum (C_6D_6) of isolated **6d**.

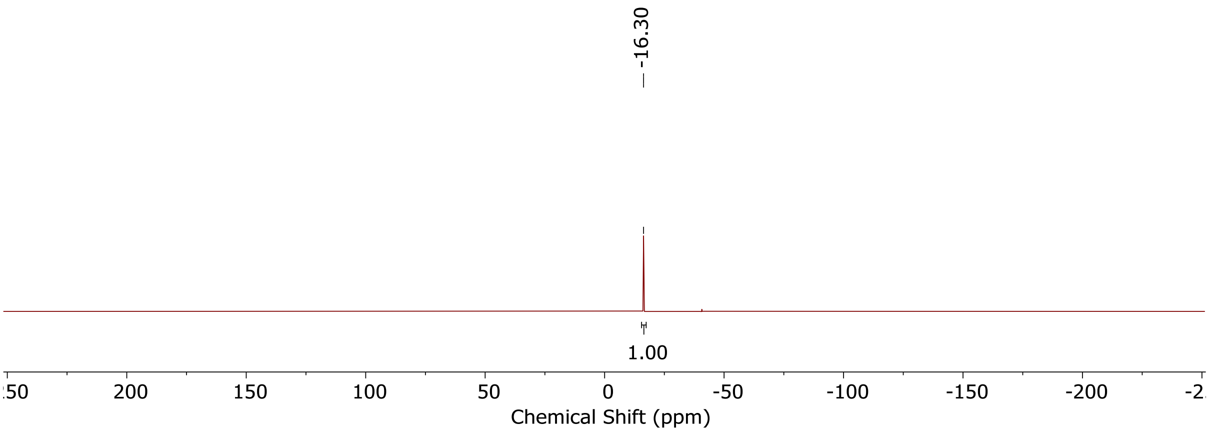

Figure S102. $^{31}P\{^{1}H\}$ NMR spectrum (C_6D_6) of isolated **6d**.

4.6. Preparatory Scale Synthesis of (4-bromophenethyl)diphenylphosphane (6e)

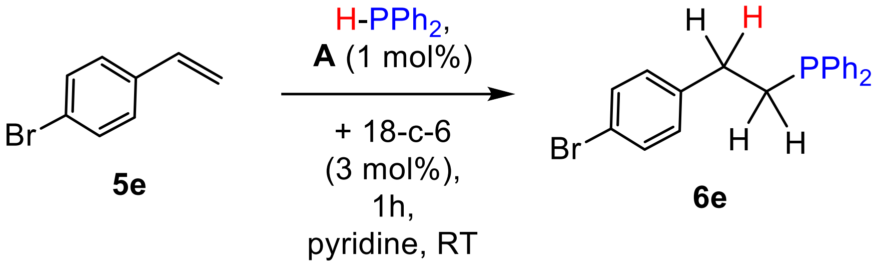

Prepared according to the general procedure with 4-bromostyrene (**5e**, 915 mg, 5 mmol), diphenylphosphine (931 mg, 5 mmol), and initiator **A** (17 mg, 0.05 mmol, 1 mol%) and 18-crown-6 (40 mg, 0.15 mmol, 3 mol%). The reaction was stirred for one hour before work-up, at which time ^{31}P NMR revealed clean formation of **6e** and no $HPPh_2$. (4-bromophenethyl)diphenylphosphane (**6e**) was isolated as a colorless oil. Upon work-up 6% $HPPh_2$ was detected by ^{31}P{^{1}H} NMR spectroscopy, this has been accounted for in the yield. Repeated attempts yielded similar outcomes, crude ^{31}P{^{1}H} NMR spectra from the aliquot has been included here.

Isolated Yield: 1.3303 g, 3.60 mmol, 72%

^{1}H NMR (400 MHz, 298 K, C_6D_6): δ = 2.07–2.11 (m, 2H, $C\mathit{H}_2CH_2PPh_2$), 2.44–2.50 (m, 2H, $CH_2C\mathit{H}_2PPh_2$), 6.56 (d, $^3J_{HH}$ = 8.23 Hz, 2H, *o*-Ar), 7.05–7.12 (m, 6H, *m*-/*p*-PPh_2), 7.21 (d, $^3J_{HH}$ = 8.23 Hz, 2H, *m*-Ar), 7.36–7.41 (m, 4H, *o*-PPh_2).

^{13}C{^{1}H} NMR (101 MHz, 298 K, C_6D_6): δ = 30.43 (d, $^2J_{CP}$ = 13.77 Hz, $\mathit{C}H_2CH_2PPh_2$), 31.92 (d, $^1J_{CP}$ = 18.70 Hz, $CH_2\mathit{C}H_2PPh_2$), 120.07 (s, Ar), 128.80 (d, $^3J_{CP}$ = 6.39 Hz, *m*-PPh_2), 128.86 (s, Ar), 130.30 (s, Ar), 131.69 (s, Ar), 133.15 (d, $^2J_{CP}$ = 18.28 Hz, *o*-PPh_2), 139.24 (d, $^1J_{CP}$ = 15.47 Hz, *i*-PPh_2), 141.77 (d, $^1J_{CP}$ = 15.47 Hz, *i*-PPh_2).

^{31}P{^{1}H} NMR (162 MHz, 298 K, C_6D_6): δ = –16.28 (s).

Mass spectrometry (APCI): $C_{20}H_{18}BrP$+H ([M+H]$^+$); Calcd. = 369.0402, Found = 369.0417.

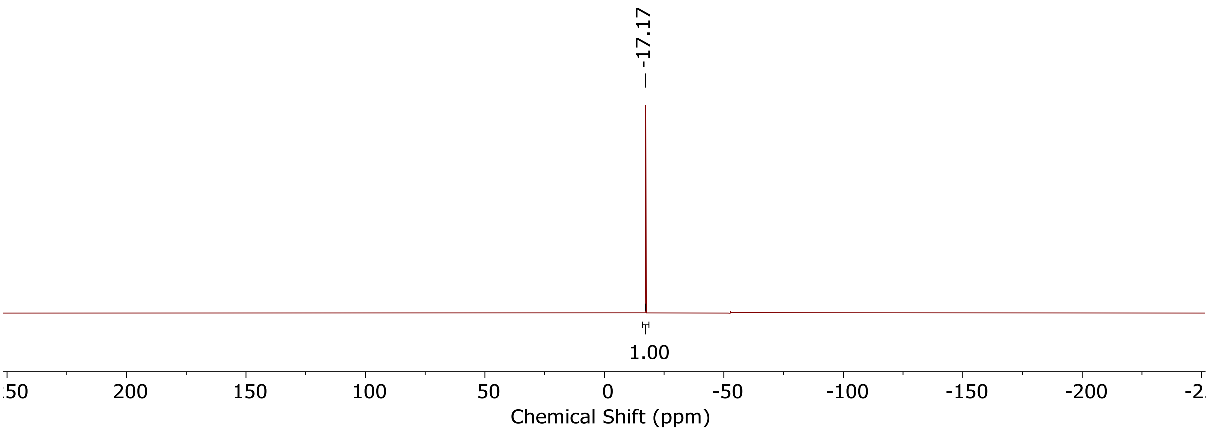

Figure S103. $^{31}P\{^{1}H\}$ NMR spectrum (pyridine) of crude **6e**.

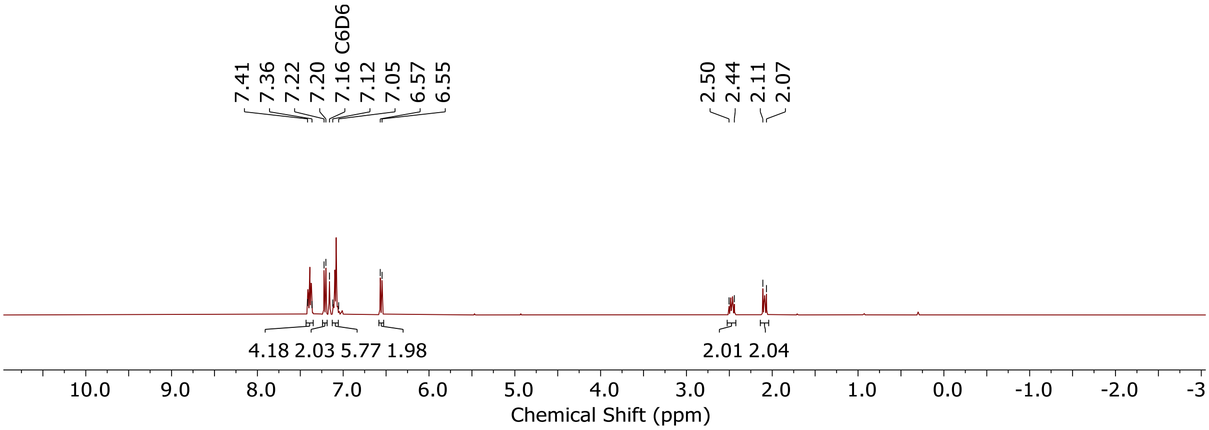

Figure S104. ^{1}H NMR spectrum (C_6D_6) of isolated **6e**.

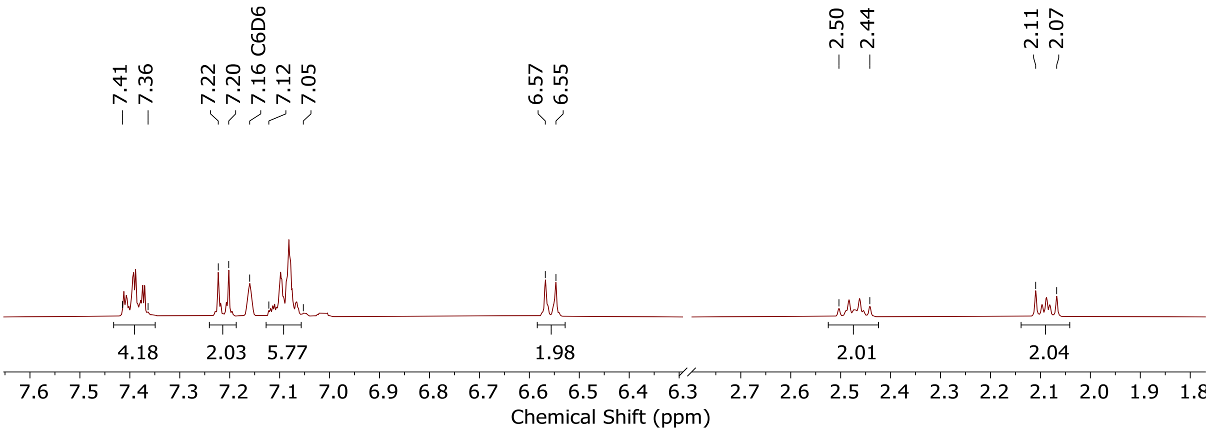

Figure S105. ^{1}H NMR spectrum (C_6D_6) of isolated **6e**. Region of 2.8–6.3 ppm removed for further clarity.

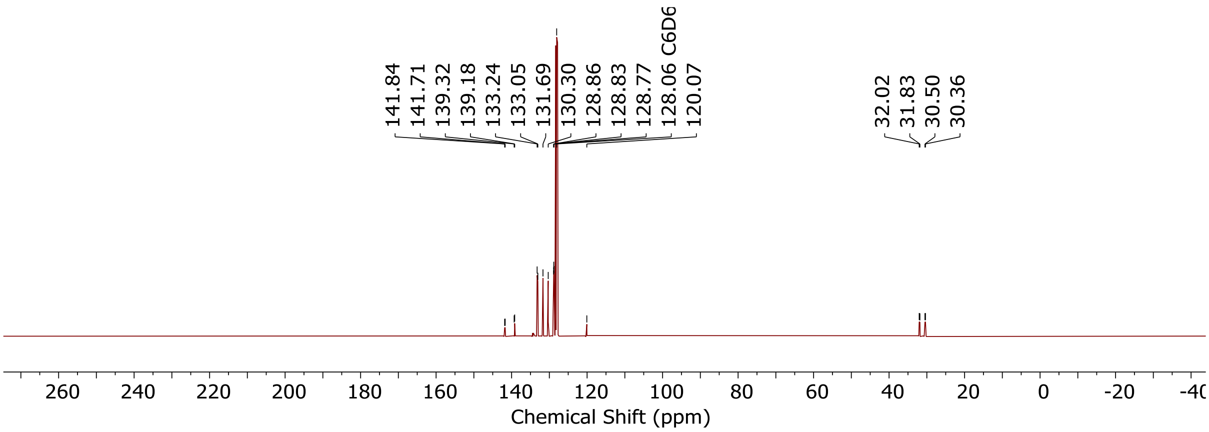

Figure S106. $^{13}C\{^{1}H\}$ NMR spectrum (C_6D_6) of isolated **6e**.

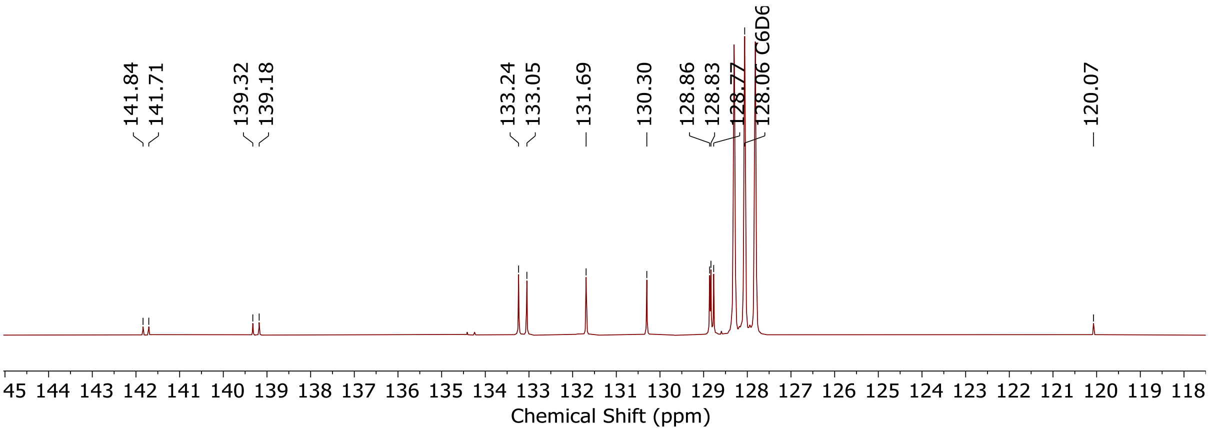

Figure S107. Zoomed in $^{13}C\{^{1}H\}$ NMR spectrum (C_6D_6) of isolated **6e**.

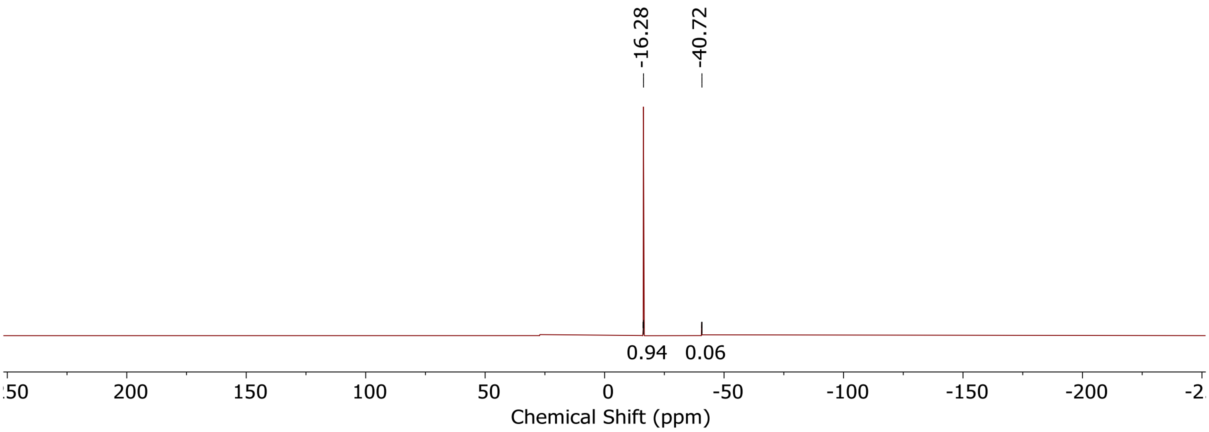

Figure S108. $^{31}P\{^{1}H\}$ NMR spectrum (C_6D_6) of isolated **6e**.

4.7. Preparatory Scale Synthesis of diphenyl(2-(triethoxysilyl)ethyl)phosphane (6g)

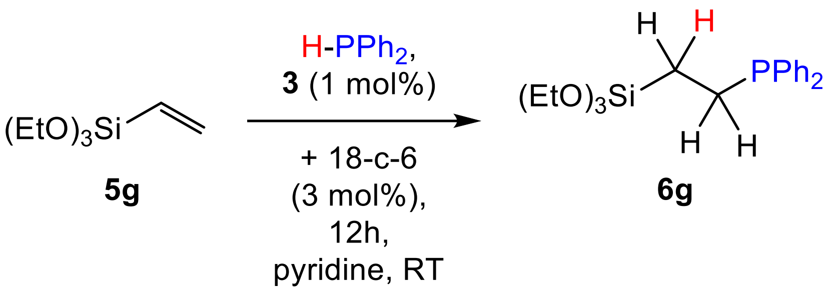

Prepared according to the general procedure with triethoxyvinylsilane (**5g**, 952 mg, 5 mmol), diphenylphosphine (931 mg, 5 mmol), and initiator **A** (17 mg, 0.05 mmol, 1 mol%) and 18-crown-6 (40 mg, 0.15 mmol, 3 mol%). The reaction was stirred for 12 hours before work-up. Diphenyl(2-(triethoxysilyl)ethyl)phosphane (**6g**) was isolated as a colorless oil.

Isolated Yield: 1.1572 g, 3.07 mmol, 61%

^{1}H NMR (400 MHz, 298 K, C_6D_6): δ = 0.89–0.95 (m, 2H, $CH_2CH_2PPh_2$), 1.11 (t, $^{3}J_{HH}$ = 7.25 Hz, 9H, CH_3CH_2O), 2.31–2.36 (m, 2H, $CH_2CH_2PPh_2$), 3.72 (q, $^{3}J_{HH}$ = 7.25 Hz, 6H, CH_3CH_2O), 7.01–7.10 (m, 6H, *m*-/*p*-PPh_2), 7.45–7.49 (m, 4H, o-PPh_2).

^{13}C{^{1}H} NMR (101 MHz, 298 K, C_6D_6): δ = 7.00 (d, $^{2}J_{CP}$ = 12.30 Hz, $CH_2CH_2PPh_2$), 18.58 (s, CH_3CH_2O), 21.38 (d, $^{1}J_{CP}$ = 14.65 Hz, $CH_2CH_2PPh_2$), 58.59 (s, CH_3CH_2O), 128.85 (s, *p*-PPh_2), 128.73 (d, $^{3}J_{CP}$ = 6.31 Hz, *m*-PPh_2), 133.21 (d, $^{2}J_{CP}$ = 18.43 Hz, *o*-PPh_2), 134.32 (d, $^{1}J_{CP}$ = 15.96 Hz, *i*-PPh_2), 139.74 (d, $^{1}J_{CP}$ = 15.96 Hz, *i*-PPh_2)

^{29}Si{^{1}H} NMR (79 MHz, 298 K, C_6D_6): δ = –47.01 (d, $^{3}J_{SiP}$ = 30.84 Hz).

^{31}P{^{1}H} NMR (162 MHz, 298 K, C_6D_6): δ = –9.35 (s).

Mass spectrometry (APCI): $C_{20}H_{29}O_3PSi$+H ($[M+H]^+$); Calcd. = 377.1696, Found = 377.1697.

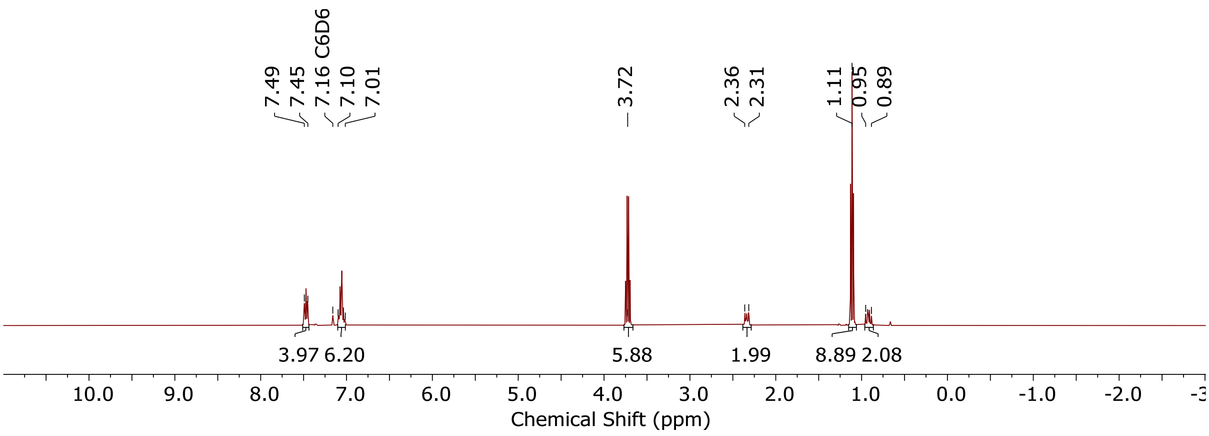

Figure S109. ^{1}H NMR spectrum (C_6D_6) of isolated **6g**.

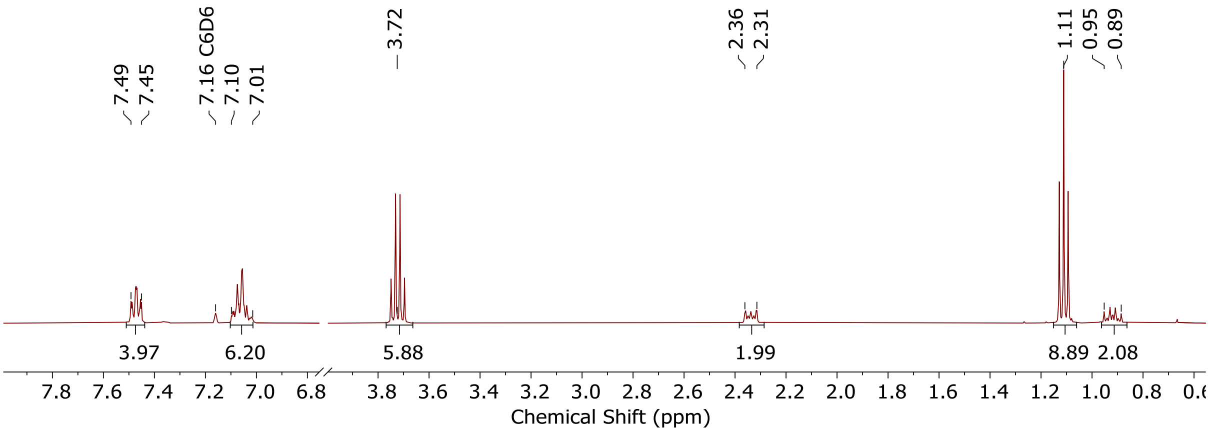

Figure S110. Zoomed in ^{1}H NMR spectrum (C_6D_6) of isolated **6g**. Region of 4.0–6.75 ppm removed for further clarity.

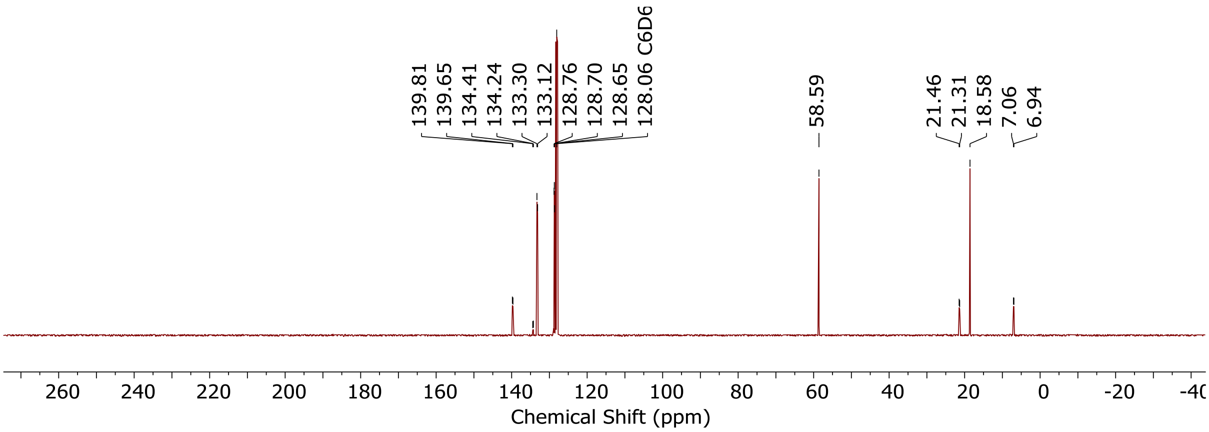

Figure S111. $^{13}C\{^{1}H\}$ NMR spectrum (C_6D_6) of isolated **6g**.

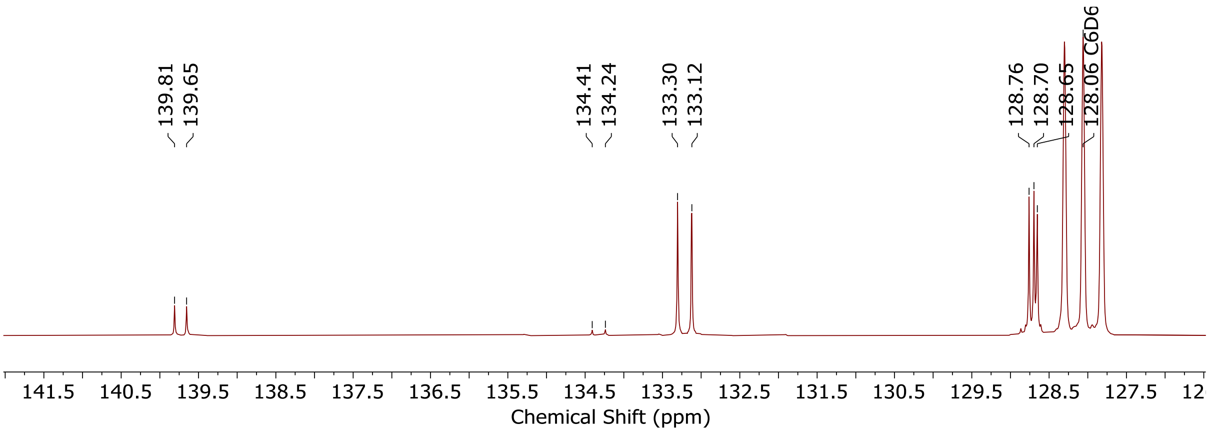

Figure S112. Zoomed in $^{13}C\{^{1}H\}$ NMR spectrum (C_6D_6) of isolated **6g**.

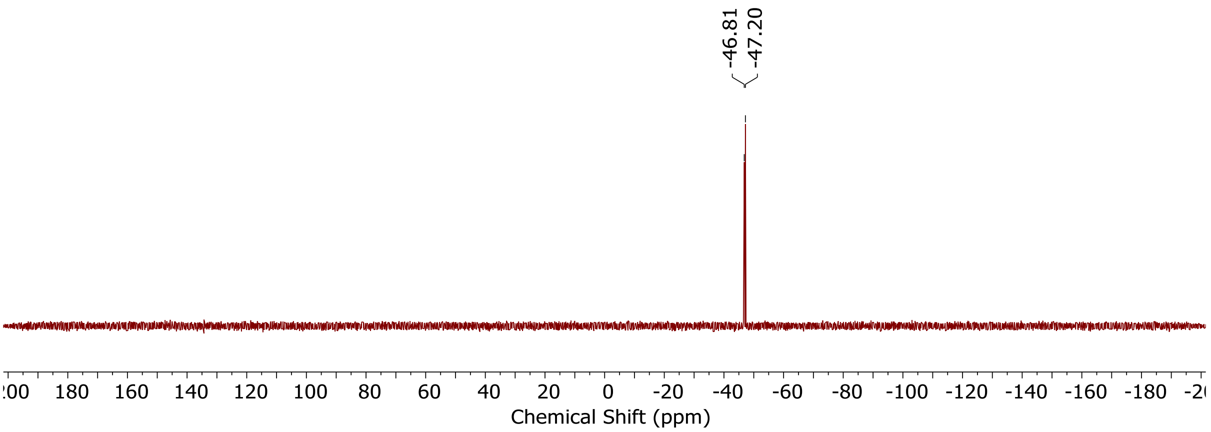

Figure S113. $^{29}Si\{^1H\}$ NMR spectrum (C_6D_6) of isolated **6g**.

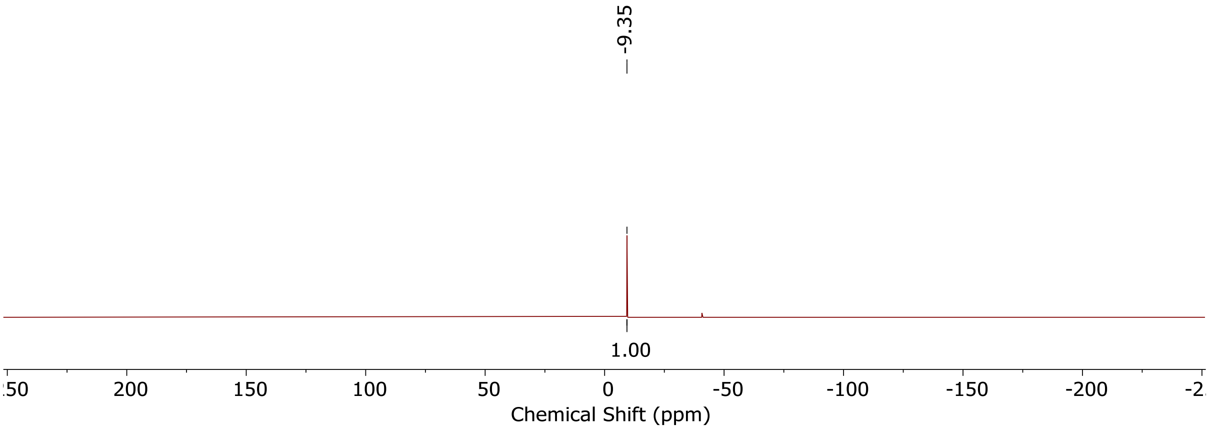

Figure S114. $^{31}P\{^1H\}$ NMR spectrum (C_6D_6) of isolated **6g**.

5. Comparing Catalytic Activity Of Different Zintl Phases

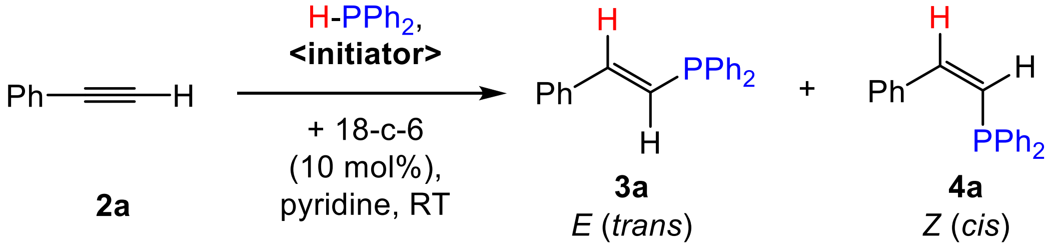

In a nitrogen glovebox, a J Young NMR tube was loaded with initiator (**A** – 3 mg, 3.33 mol%; **B** – 3.3 mg, 3.33 mol%; **C** – 6.4 mg, 3.33 mol%; **D** – 3.3 mg, 3.33 mol%; **E** – 9.7 mg, 3.33 mol%; **F** – 6.2 mg, 2 mol%; **G** – 6.1 mg, 2.5 mol%; **H** – 9.2 mg, 2.5 mol%) and where indicated 18-crown-6 (8 mg, 10 mol%) was added. Phenylacetylene (**2a**, 33 µL, 0.3 mmol) in pyridine (0.6 mL) was transferred to the J Young NMR tube followed by the addition of diphenylphosphine (52.2 µL, 0.3 mmol) *via* microsyringe. The reaction progress was monitored by $^{31}P\{^1H\}$ NMR spectroscopy, completion was determined by the absence of diphenylphosphine ($^{31}P\{^1H\}$ NMR (162 MHz, 298 K, pyridine): δ = 40.24 ppm).

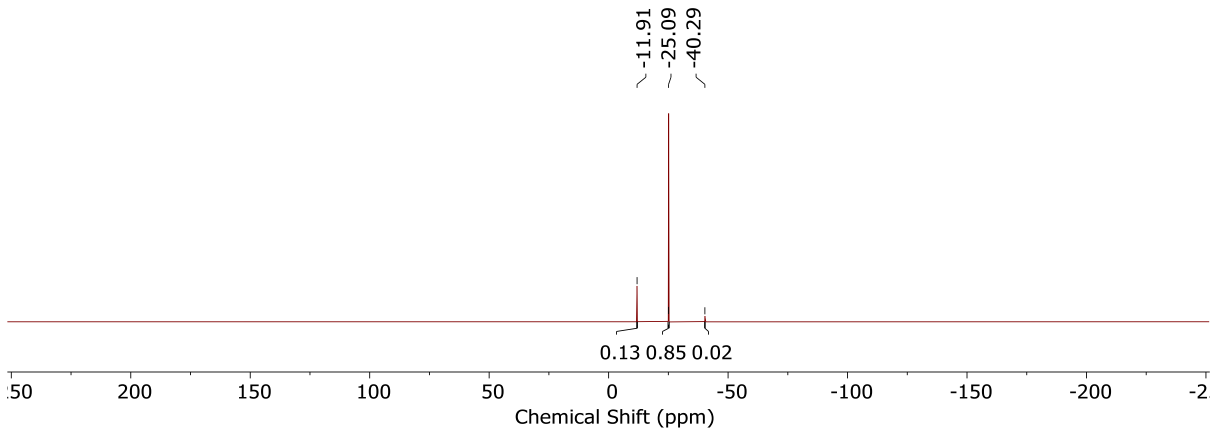

Figure S115. $^{31}P\{^{1}H\}$ NMR spectrum (pyridine) of crude **3a**/**4a** after 12h at RT catalysed by 3.33 mol% **A**.

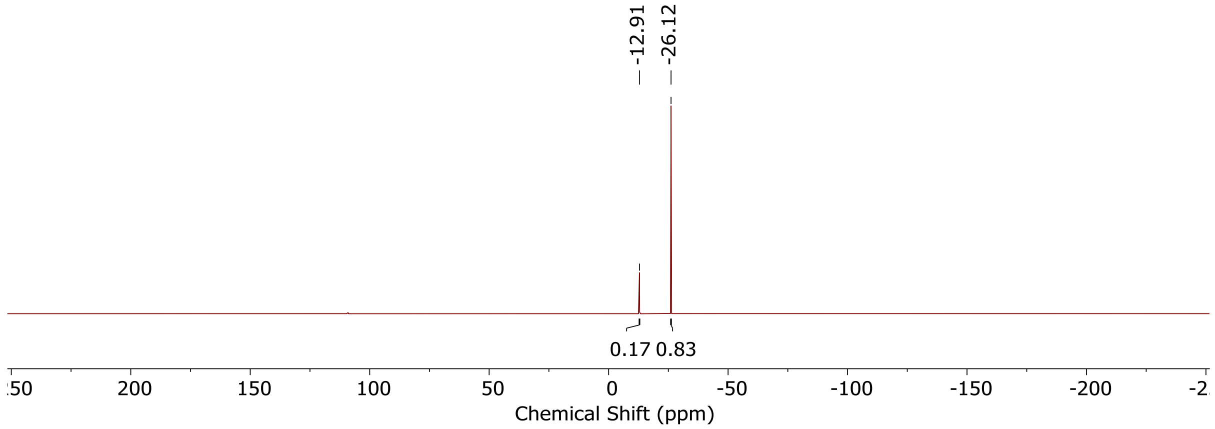

Figure S116. $^{31}P\{^{1}H\}$ NMR spectrum (pyridine) of crude **3a**/**4a** after 15 minutes at RT catalysed by 3.33 mol% **A** + 10 mol% 18-c-6.

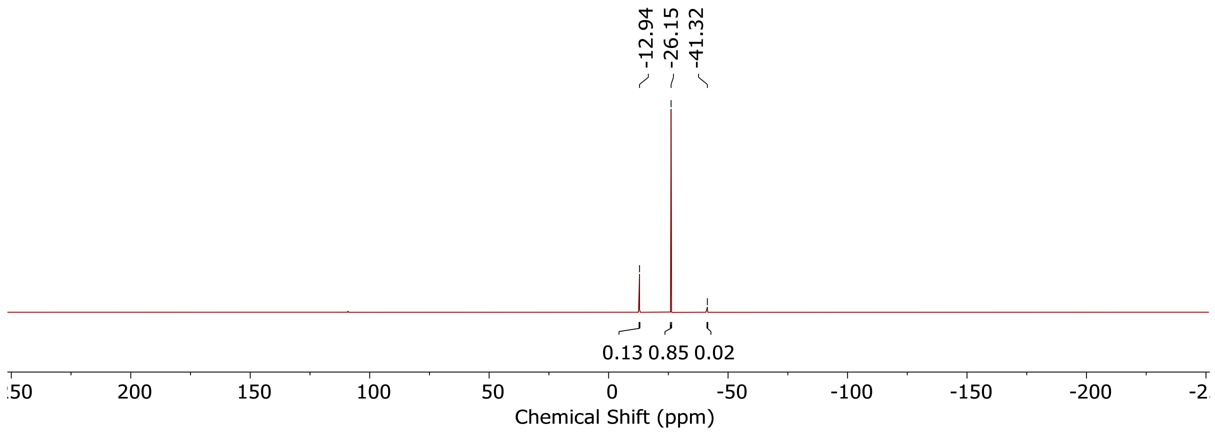

Figure S117. $^{31}P\{^{1}H\}$ NMR spectrum (pyridine) of crude **3a**/**4a** after 6h at RT catalysed by 3.33 mol% **B**.

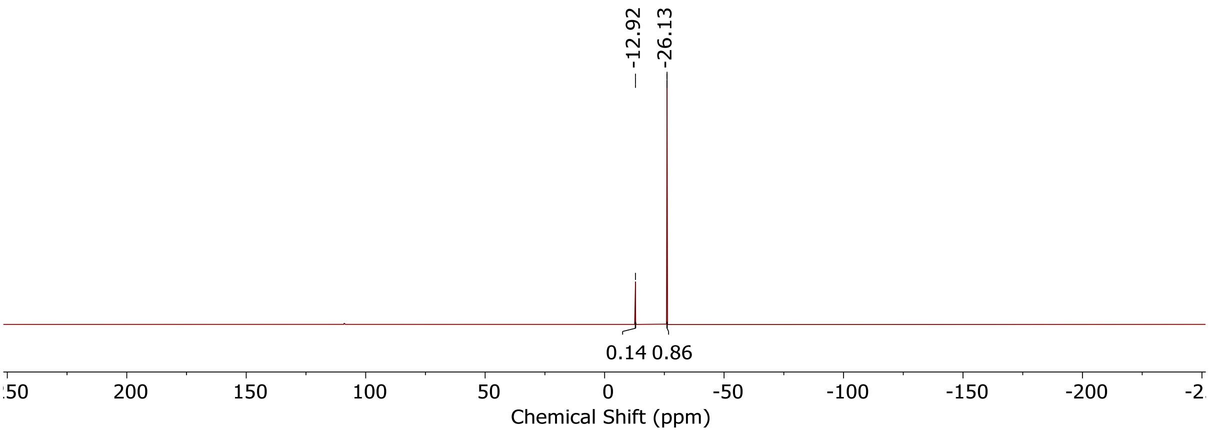

Figure S118. $^{31}P\{^1H\}$ NMR spectrum (pyridine) of crude **3a**/**4a** after 15 minutes at RT catalysed by 3.33 mol% **B** + 10 mol% 18-c-6.

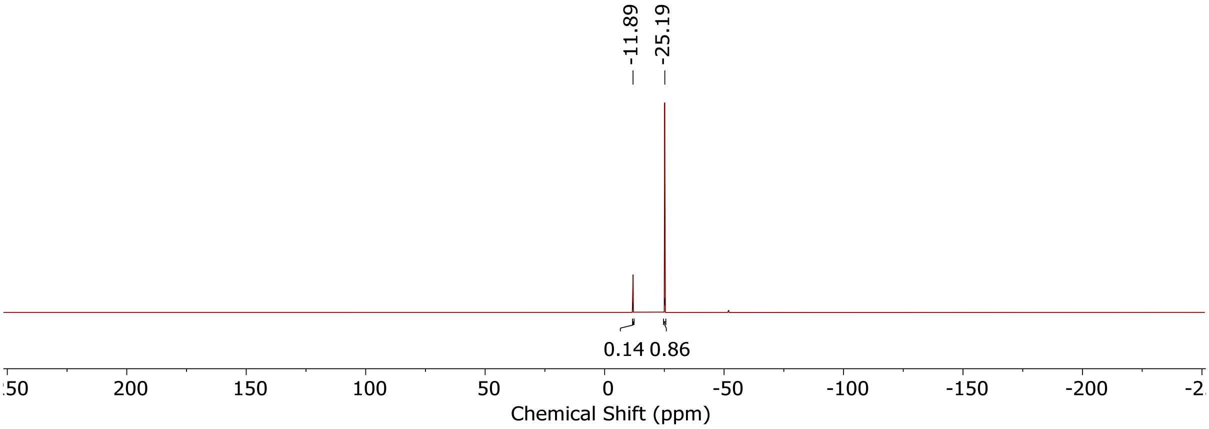

Figure S119. $^{31}P\{^1H\}$ NMR spectrum (pyridine) of crude **3a**/**4a** after 1h at RT catalysed by 3.33 mol% **C**.

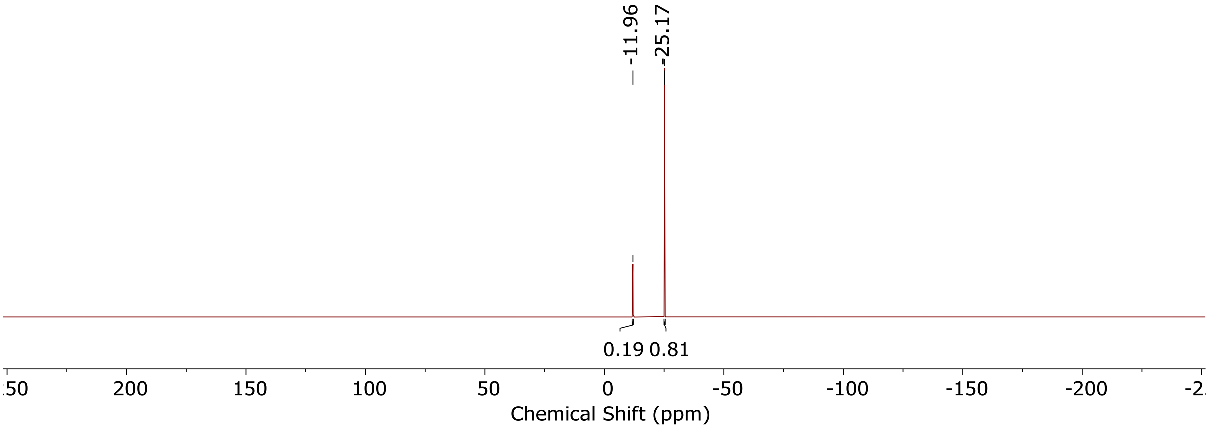

Figure S120. $^{31}P\{^1H\}$ NMR spectrum (pyridine) of crude **3a**/**4a** after 15 minutes at RT catalysed by 3.33 mol% **C** + 10 mol% 18-c-6.

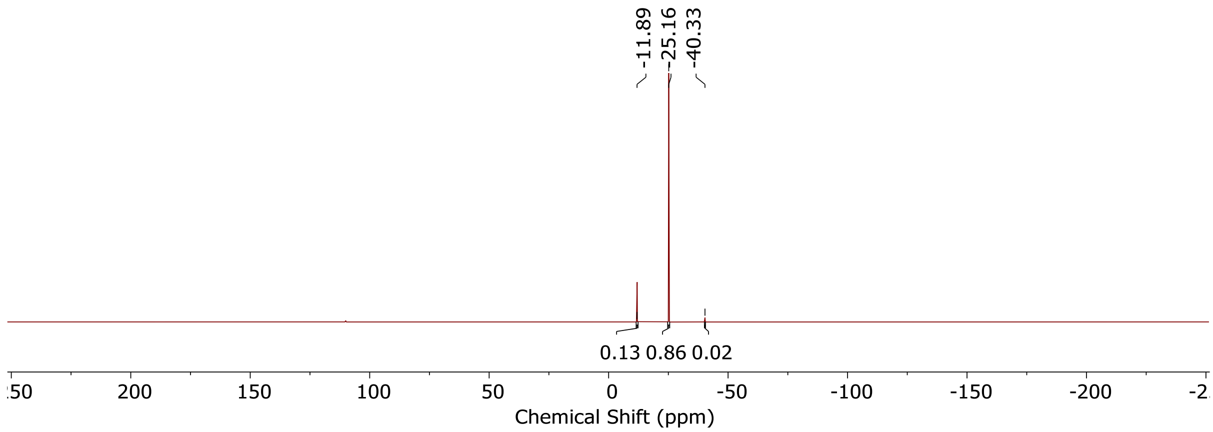

Figure S121. $^{31}P\{^{1}H\}$ NMR spectrum (pyridine) of crude **3a**/**4a** after 18h at RT catalysed by 3.33 mol% **D**.

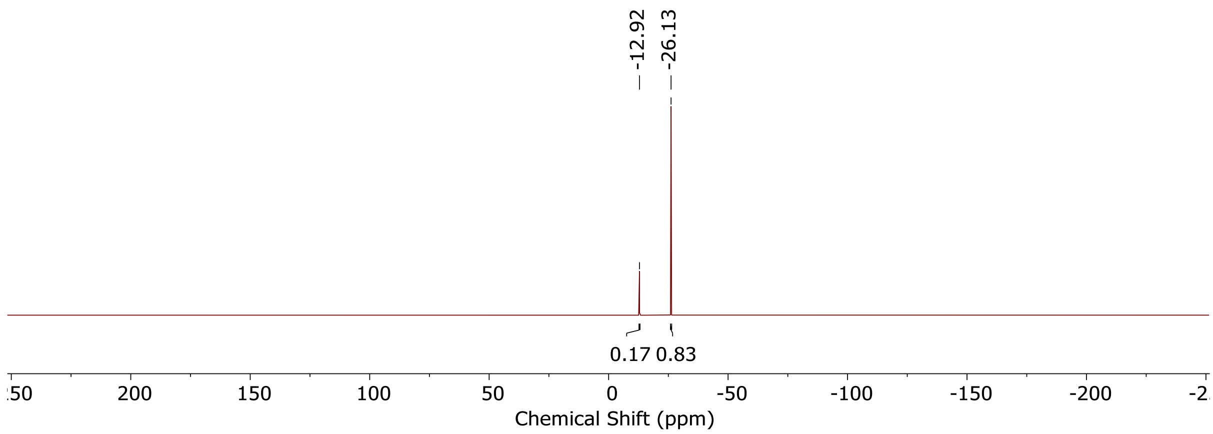

Figure S122. $^{31}P\{^{1}H\}$ NMR spectrum (pyridine) of crude **3a**/**4a** after 15 minutes at RT catalysed by 3.33 mol% **D** + 10 mol% 18-c-6.

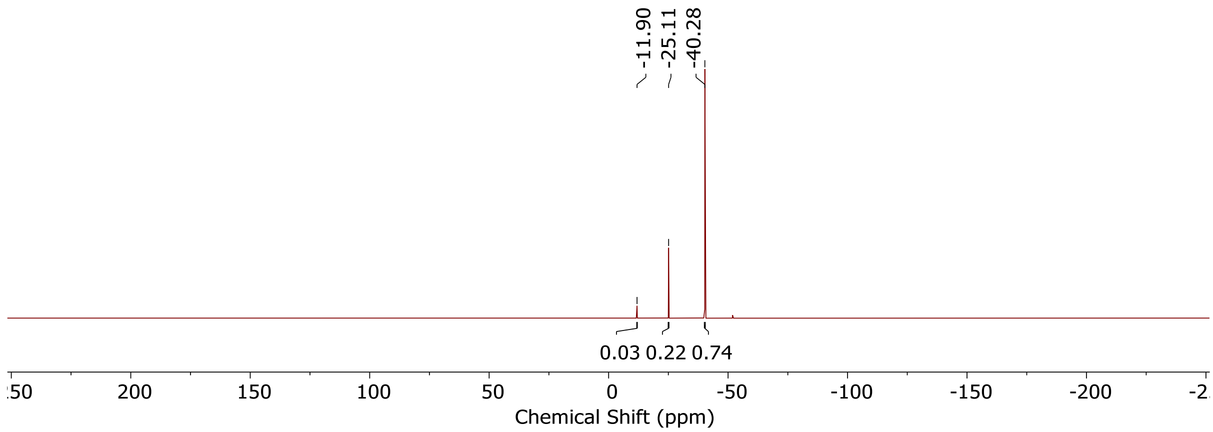

Figure S123. $^{31}P\{^{1}H\}$ NMR spectrum (pyridine) of crude **3a**/**4a** after 24h at RT catalysed by 3.33 mol% **E**.

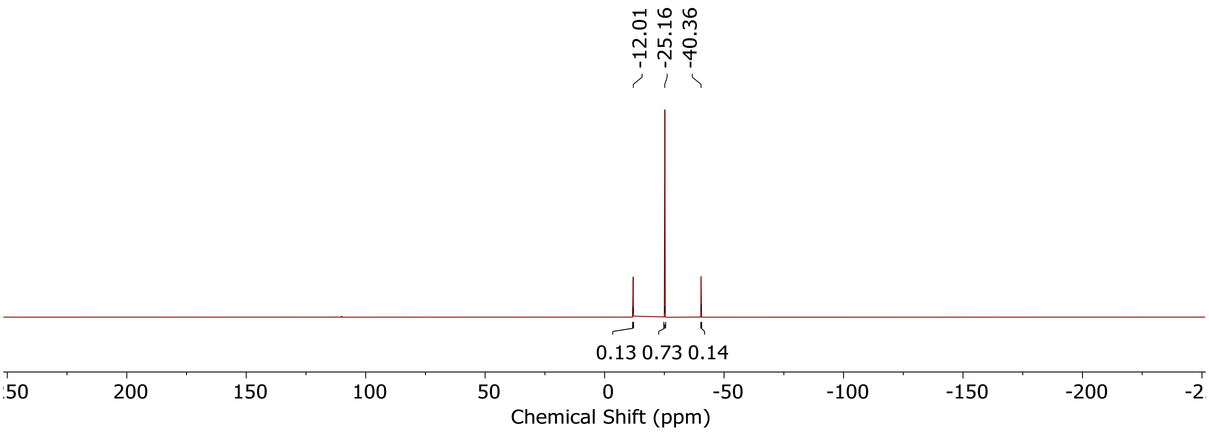

Figure S124. $^{31}P\{^{1}H\}$ NMR spectrum (pyridine) of crude **3a**/**4a** after 24h at RT catalysed by 3.33 mol% **E** + 10 mol% 18-c-6.

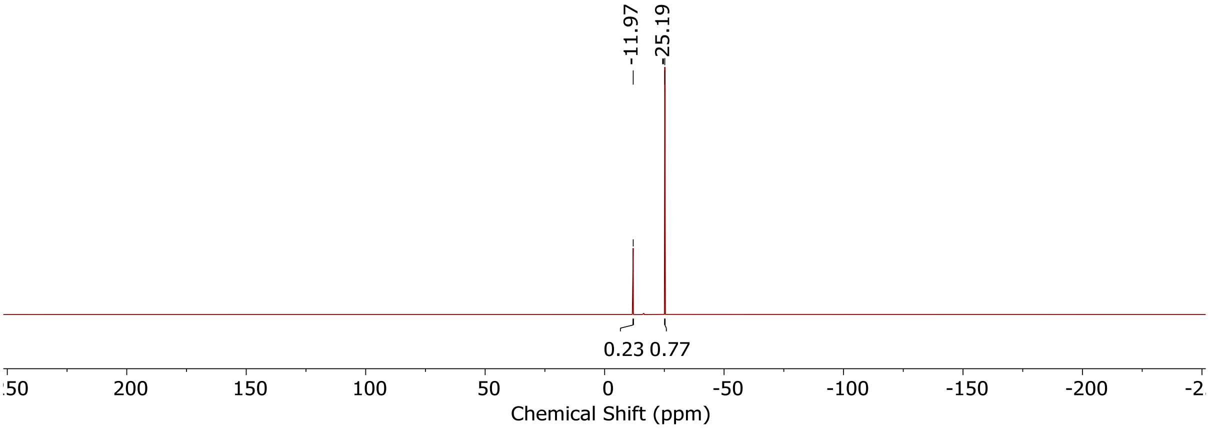

Figure S125. $^{31}P\{^{1}H\}$ NMR spectrum (pyridine) of crude **3a**/**4a** after 1h at RT catalysed by 2 mol% **F**.

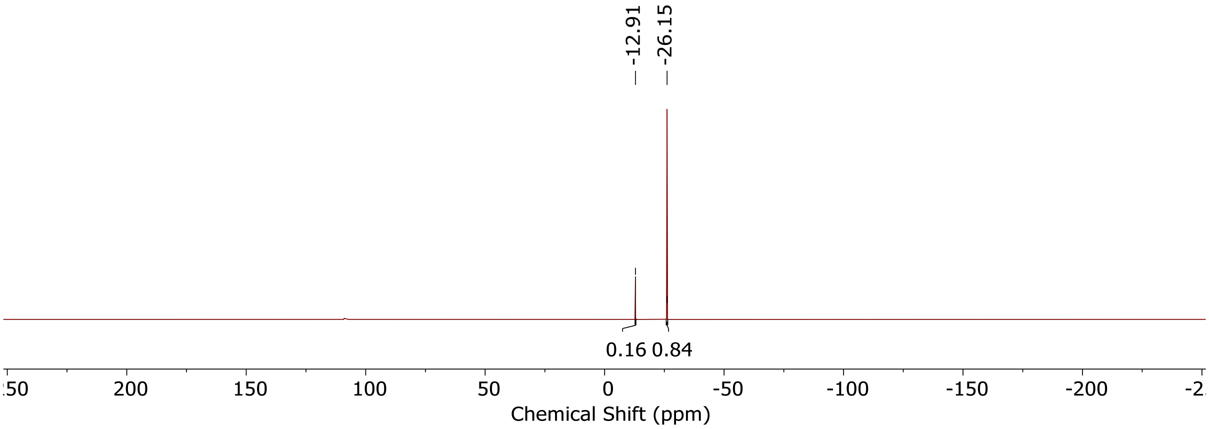

Figure S126. $^{31}P\{^{1}H\}$ NMR spectrum (pyridine) of crude **3a**/**4a** after 15 minutes at RT catalysed by 2 mol% **F** + 10 mol% 18-c-6.

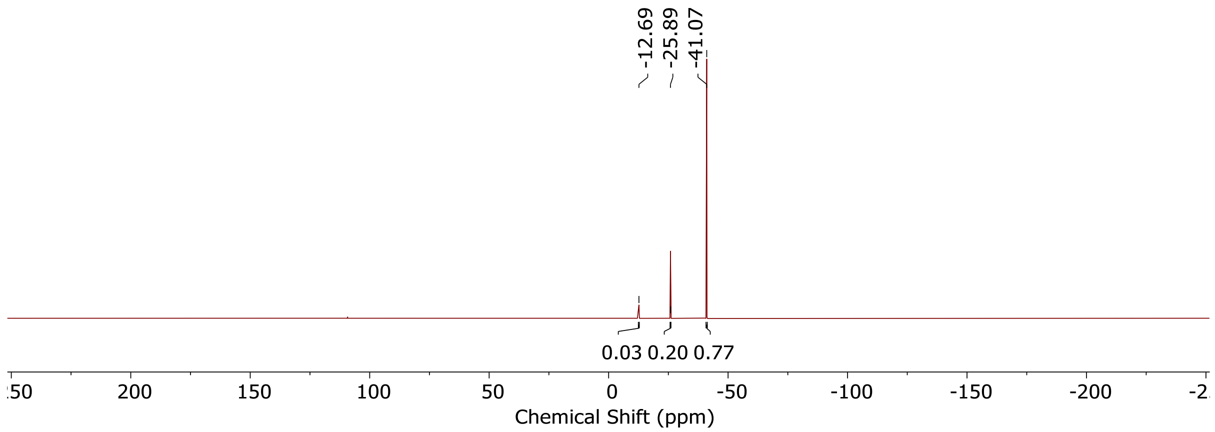

Figure S127. $^{31}P\{^{1}H\}$ NMR spectrum (pyridine) of crude **3a**/**4a** after 24h at RT catalysed by 2.5 mol% **G**.

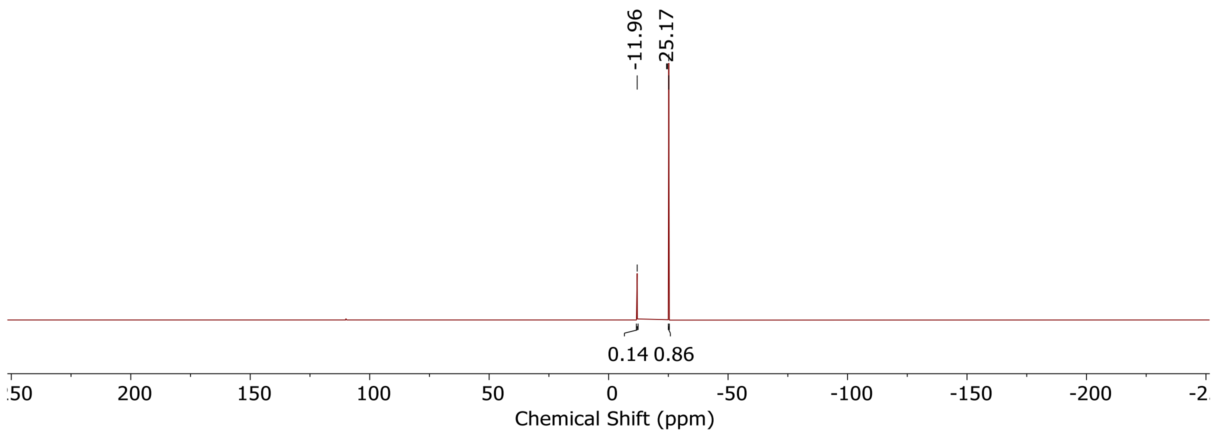

Figure S128. $^{31}P\{^{1}H\}$ NMR spectrum (pyridine) of crude **3a**/**4a** after 1h at RT catalysed by 2.5 mol% **G** + 10 mol% 18-c-6.

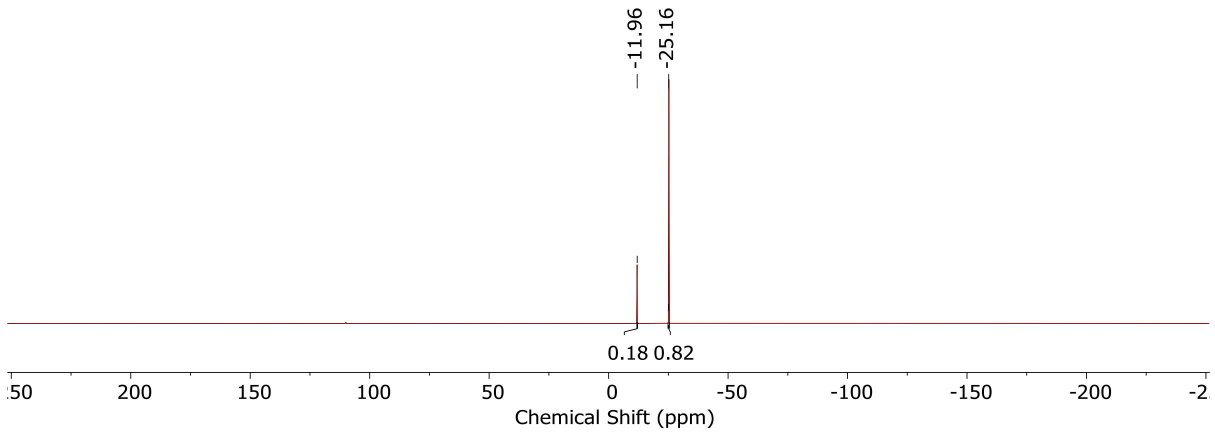

Figure S129. $^{31}P\{^{1}H\}$ NMR spectrum (pyridine) of crude **3a**/**4a** after 1h at RT catalysed by 2.5 mol% **H**.

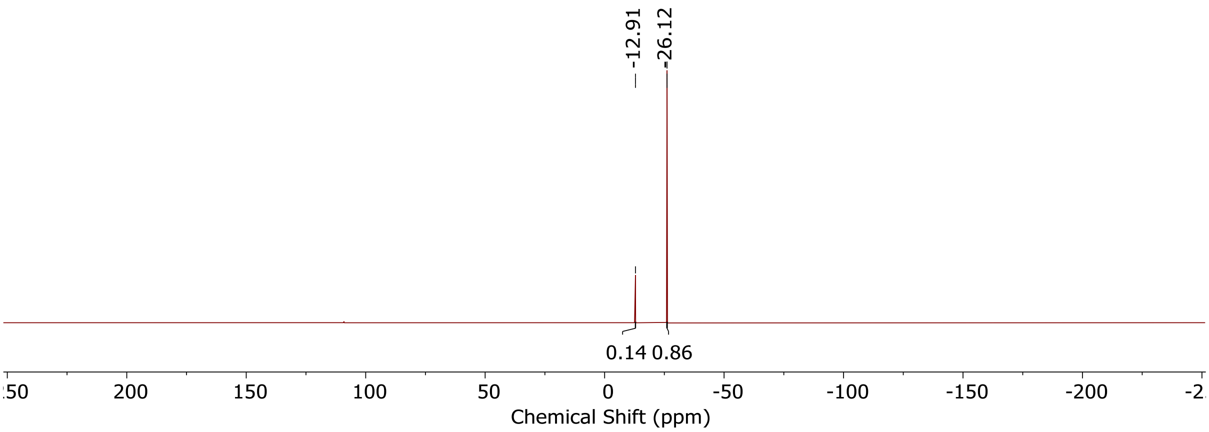

Figure S130. $^{31}P\{^1H\}$ NMR spectrum (pyridine) of crude **3a**/**4a** after 15 minutes at RT catalysed by 2.5 mol% **H** + 10 mol% 18-c-6.

6. Stoichiometric Reactions of $HPPh_2$ with Zintl Ions/ Phases

Zintl ion/phase (0.03 mmol) and 18-crown-6 (3 – 5 equivalents depending on the number of anionic sites) was added to a J Young NMR tube followed by the addition of pyridine and diphenylphosphine (3 – 5 equivalents depending on the number of anionic sites) by microsyringe. The reaction was monitored at room temperature (RT) by $^{31}P\{^1H\}$ NMR spectroscopy for 1h.

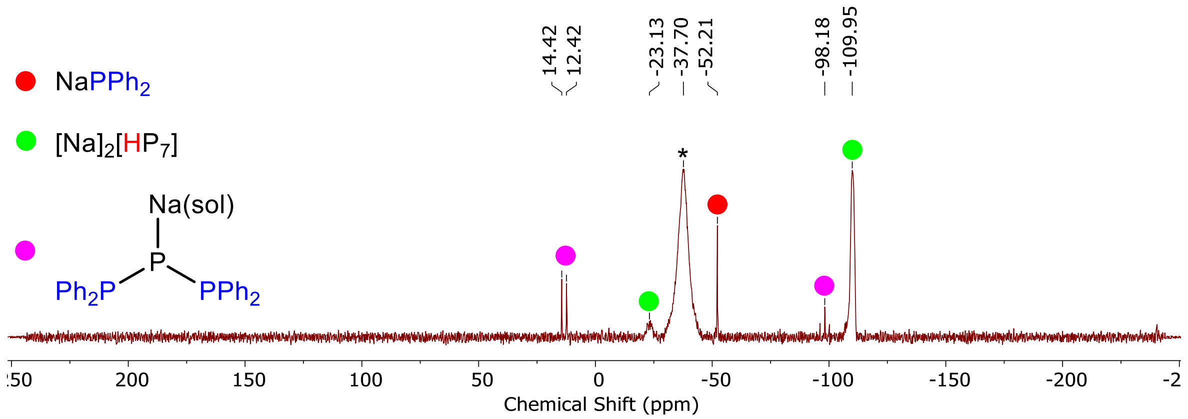

Figure S131. $^{31}P\{^1H\}$ NMR spectrum (pyridine) of **A** + 3 $HPPh_2$ + 3 18-c-6 after 1h at RT. * denotes $HPPh_2$.

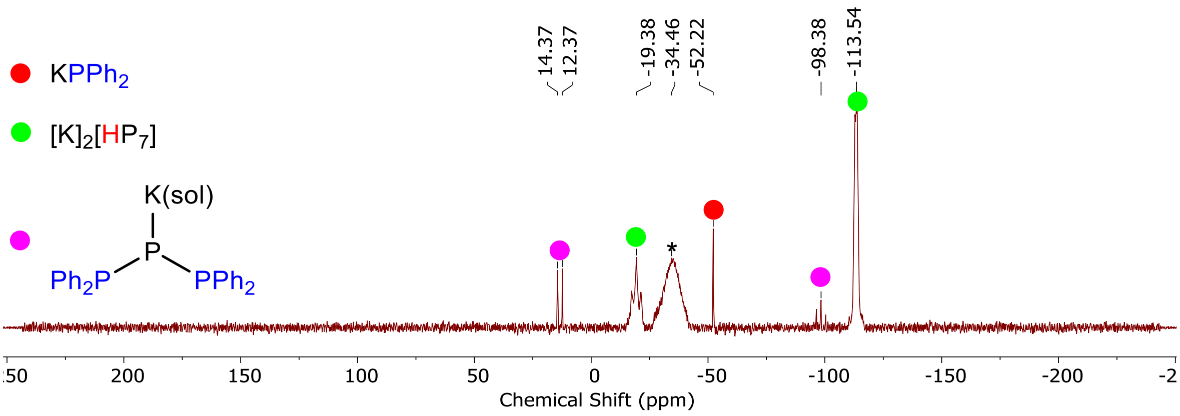

Figure S132. $^{31}P\{^1H\}$ NMR spectrum (pyridine) of **B** + 3 $HPPh_2$ + 3 18-c-6 after 1h at RT. * denotes $HPPh_2$.

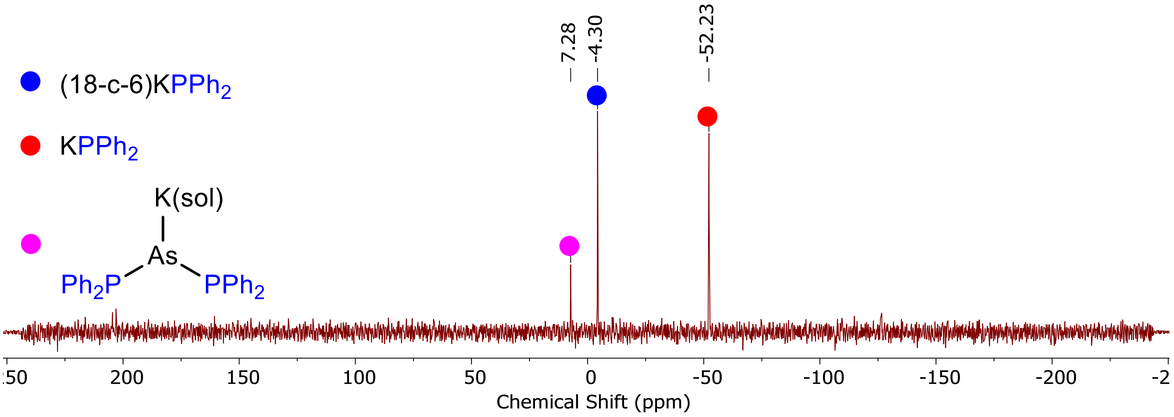

Figure S133. $^{31}P\{^1H\}$ NMR spectrum (pyridine) of **C** + 3 $HPPh_2$ + 3 18-c-6 after 1h at RT. Assignment of $K[As(PPh_2)_2]$ is based on comparing the chemical shift to literature known (TMEDA)(THF)Li[As(P^tBu)$_2$(μ-P^tBu)].[16]

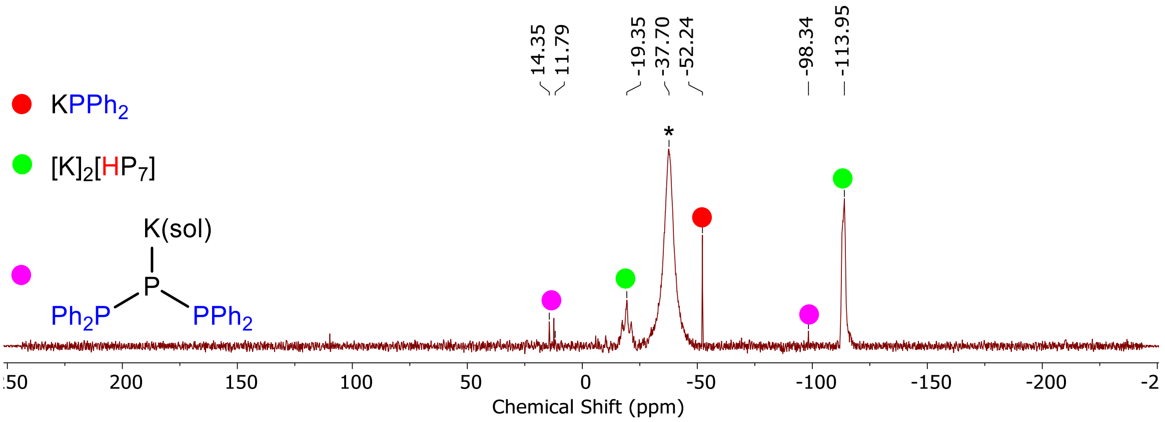

Figure S134. $^{31}P\{^1H\}$ NMR spectrum (pyridine) of **D** + 3 $HPPh_2$ + 3 18-c-6 after 1h at RT. * denotes $HPPh_2$.

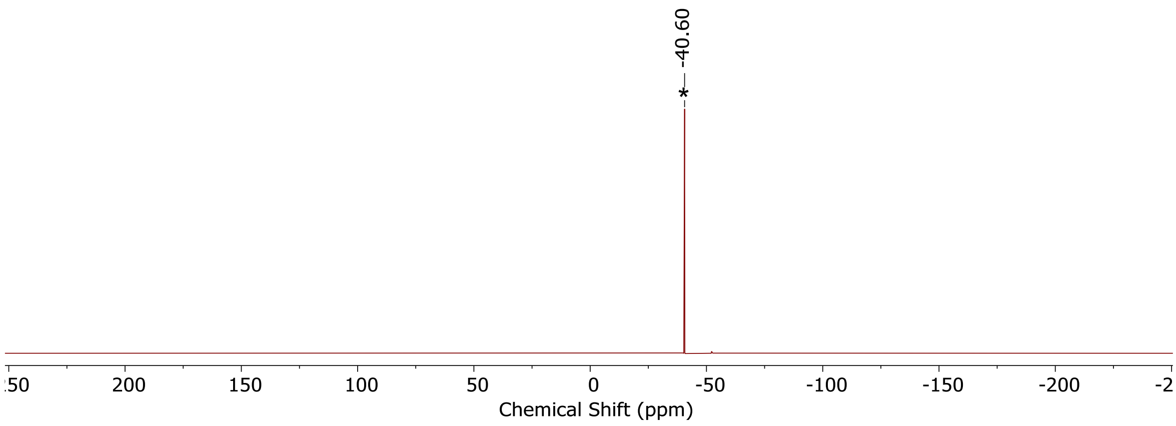

Figure S135. $^{31}P\{^{1}H\}$ NMR spectrum (pyridine) of **E** + 3 $HPPh_2$ + 3 18-c-6 after 1h at RT. No reaction occurred. * denotes $HPPh_2$.

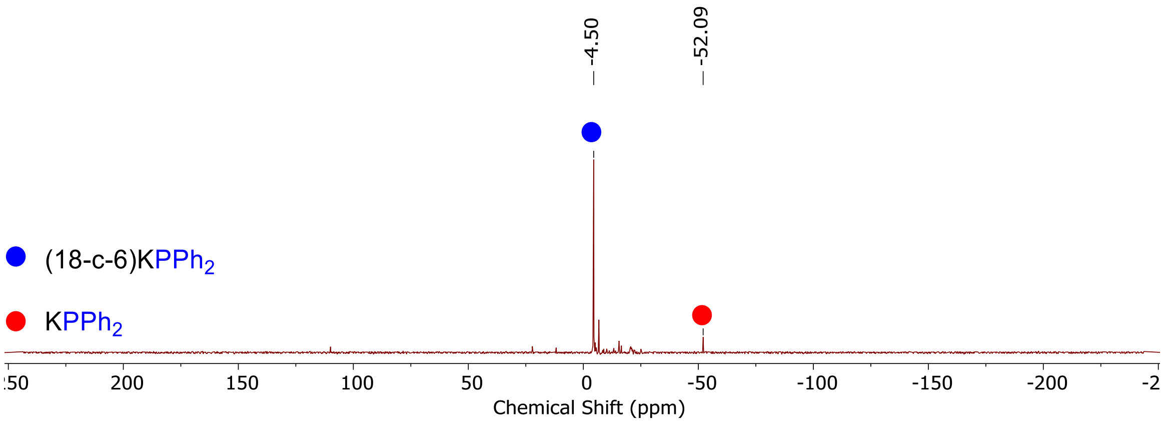

Figure S136. $^{31}P\{^{1}H\}$ NMR spectrum (pyridine) of **F** + 5 $HPPh_2$ + 5 18-c-6 after 1h at RT.

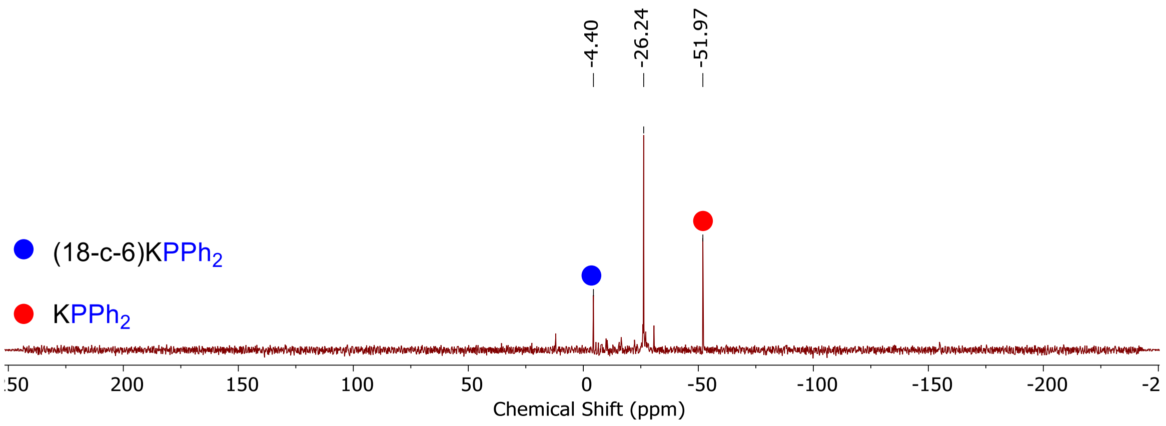

Figure S137. $^{31}P\{^{1}H\}$ NMR spectrum (pyridine) of **G** + 4 $HPPh_2$ + 4 18-c-6 after 1h at RT. Unknown species at –26.24 ppm.

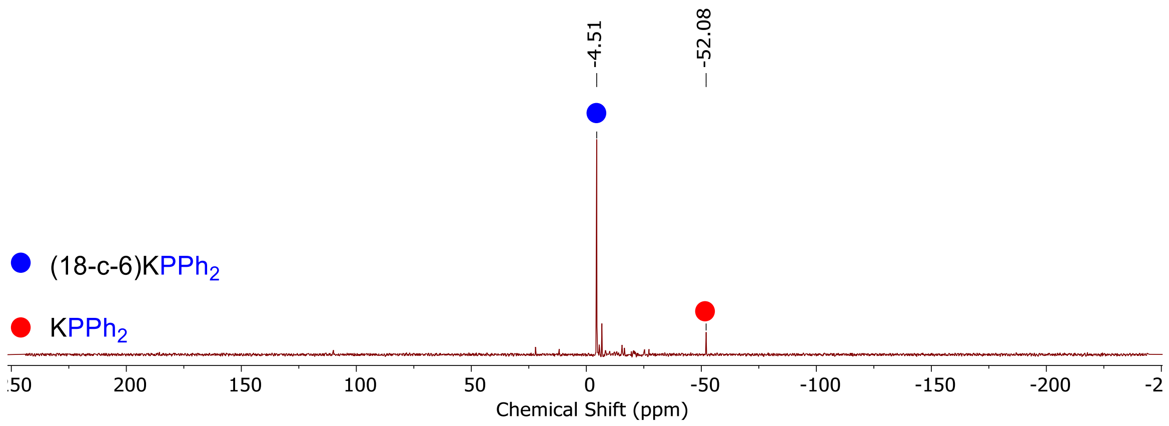

Figure S138. $^{31}P\{^1H\}$ NMR spectrum (pyridine) of **H** + 4 $HPPh_2$ + 4 18-c-6 after 1h at RT.

7. Stoichiometric Reactions of Substrates with Zintl Ion A

Zintl ion **A** (9 mg, 0.03 mmol) and 18-crown-6 (24 mg, 0.09 mmol) was added to a J Young NMR tube in pyridine followed by the addition of 3 equivalents of organic substrate **2a**, **5a**, or **7a**. The reaction was monitored at room temperature (RT) by $^{31}P\{^1H\}$ NMR spectroscopy after 15 minutes, 3 h, and 24 h. No reaction was observed by ^{31}P NMR spectroscopy for substrate **2a** after 15 minutes, and substrates **5a** or **7a** after 24 h. After 3 h, the reaction mixture with substrate **2a** displayed 3 new signals by ^{31}P NMR spectroscopy, consistent with cluster decomposition.

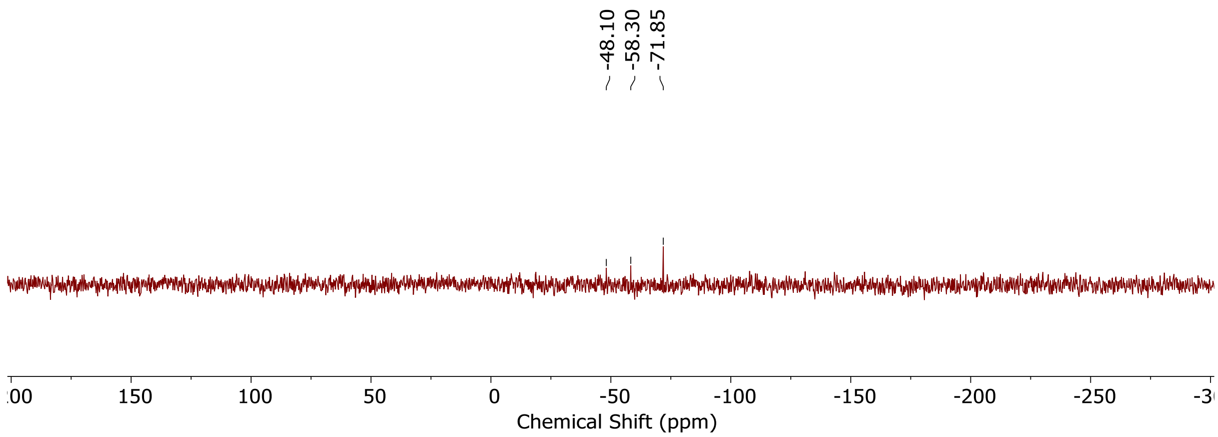

Figure S139. $^{31}P\{^1H\}$ NMR spectrum (pyridine) of **A** + 3 18-c-6 + 3 PhCCH (**2a**) after 3h at RT.

8. References

(1) a) Okamoto, S.; Ariki, R.; Tsujioka, H.; Sudo, A. A Metal-Free Approach to 1,2-Diamines via Visible Light-Driven Reductive Coupling of Imines with Perylene as a Photoredox Catalyst. *J. Org. Chem.* **2017**, *82* (18), 9731–9736. b) Fabio, M.;

Ronzini, L.; Troisi, L. Synthesis of Stable Isoxazolines by [3+2] Cycloaddition of Oxaziridines with Alkynes. *Tetrahedron* **2008**, *64* (22), 4979–4984.
(2) van Ijzendoorn, B.; Albawardi, S. F.; Vitorica-Yrezabal, I. J.; Whitehead, G. F. S.; McGrady, J. E.; Mehta, M. A Zintl Cluster for Transition Metal-Free Catalysis: C═O Bond Reductions. *J. Am. Chem. Soc.* **2022**, *144* (46), 21213–21223.
(3) Cicač-Hudi, M.; Bender, J.; Schlindwein, S. H.; Bispinghoff, M.; Nieger, M.; Grützmacher, H.; Gudat, D. Direct Access to Inversely Polarized Phosphaalkenes from Elemental Phosphorus or Polyphosphides. *Eur. J. Inorg. Chem.* **2016**, *2016* (5), 649–658.
(4) Jobbins, W. D.; van Ijzendoorn, B.; Vitorica-Yrezabal, I. J.; Whitehead, G. F. S.; Mehta, M. Reactivity of Tetrel Functionalized Heptapnictogen Clusters Towards Heteroallenes. *Dalton Trans.* **2023**, *52* (8), 2384–2391.
(5) Santandrea, R. P.; Mensing, C.; Von Schnering, H. G. The Sublimation and Thermodynamic Properties of the Alkali Metal Phosphides $Na_3P_{7(s)}$, $K_3P_{7(s)}$, $Rb_3P_{7(s)}$ and $Cs_3P_{7(s)}$11Chemistry and Structural Chemistry of Phosphides and Polyphosphides *Thermochim. Acta* **1986**, *98*, 301–311.
(6) Hirschle, C.; Röhr, C. Darstellung und Kristallstruktur der bekannten Zintl-Phasen Cs_3Sb_7 und Cs_4Sb_2. *Z. Anorg. Allg. Chem.* **2000**, *626* (9), 1992–1998.
(7) Von Schnering, H. G.; Baitinger, M.; Bolle, U.; Carrillo-Cabrera, W.; Curda, J.; Grin, Y.; Heinemann, F.; Llanos, J.; Peters, K.; Schmeding, A.; et al. Binary Alkali Metal Compounds with the Zintl Anions $[Ge_9]^{4-}$ and $[Sn_9]^{4-}$. *Z. Anorg. Allg. Chem.* **1997**, *623* (7), 1037–1039.
(8) Cisar, A.; Corbett, J. D. Polybismuth anions. Synthesis and Crystal Structure of a Salt of the Tetrabismuthide(2-) ion, Bi_4^{2-}. A Basis for the Interpretation of the Structure of Some Complex Intermetallic Phases. *Inorg. Chem.* **1977**, *16* (10), 2482–2487.
(9) Kuveke, R. E. H.; Barwise, L.; van Ingen, Y.; Vashisth, K.; Roberts, N.; Chitnis, S. S.; Dutton, J. L.; Martin, C. D.; Melen, R. L. An International Study Evaluating Elemental Analysis. *ACS Cent. Sci.* **2022**, *8* (7), 855–863.
(10) a) Lee, K.; Huang, Y.; Corrigan, J. F. Synthesis and Reaction Chemistry of Zinc-Diarylphosphido Clusters with Phosphorus Precursors. *Eur. J. Inorg. Chem.* **2020**, *2020* (1), 57–63. b) Branfoot, C.; Young, T. A.; Wass, D. F.; Pringle, P. G. Radical-initiated P,P-metathesis Reactions of Diphosphanes: Evidence from Experimental and Computational Studies. *Dalton Trans.* **2021**, *50* (20), 7094–7104. c) You, Q.; Zhang, J.; Zhang, F.; Cai, J.; Zhou, X. Cooperative Rare-Earth/Lithium-Mediated Conversion of White Phosphorus. *Chem. Eur. J.* **2023**, *29* (22), e202203679.
(11) Pollard, V. A.; Young, A.; McLellan, R.; Kennedy, A. R.; Tuttle, T.; Mulvey, R. E. Lithium-Aluminate-Catalyzed Hydrophosphination Applications. *Angew. Chem. Int. Ed.* **2019**, *58* (35), 12291–12296.
(12) a) Hayashi, M.; Matsuura, Y.; Watanabe, Y. Regio- and Stereoselective Synthesis of Alkenylphosphines: A Rhodium-Catalyzed Hydrophosphination of Alkynes Using a Silylphosphine. *J. Org. Chem.* **2006**, *71* (24), 9248–9251. b) Itazaki, M.; Katsube, S.; Kamitani, M.; Nakazawa, H. Synthesis of Vinylphosphines and Unsymmetric Diphosphines: Iron-Catalyzed Selective Hydrophosphination Reaction of Alkynes and Vinylphosphines with Secondary Phosphines. *Chem. Commun.* **2016**, *52* (15), 3163–3166.
(13) a) Barrett, A. N.; Sanderson, H. J.; Mahon, M. F.; Webster, R. L. Hydrophosphination using $[GeCl\{N(SiMe_3)_2\}_3]$ as a Pre-catalyst. *Chem. Commun.* **2020**, *56* (88), 13623–13626. b) Yuan, J.; Hu, H.; Cui, C. N-Heterocyclic Carbene–

Ytterbium Amide as a Recyclable Homogeneous Precatalyst for Hydrophosphination of Alkenes and Alkynes. *Chem. Eur. J.* **2016**, *22* (16), 5778–5785.
(14) Nolla-Saltiel, R.; Geer, A. M.; Taylor, L. J.; Churchill, O.; Davies, E. S.; Lewis, W.; Blake, A. J.; Kays, D. L. Hydrophosphination of Activated Alkenes by a Cobalt(I) Pincer Complex. *Adv. Synth. Catal.* **2020**, *362* (15), 3148–3157.
(15) Bar-Nir Ben-Aroya, B.; Portnoy, M. Preparation of α-Aminophosphines on Solid Support: Model Studies and Parallel Synthesis. *Tetrahedron* **2002**, *58* (25), 5147–5158.
(16) A. Beswick, M.; D. Hopkins, A.; E. G. Mosquera, M.; R. Raithby, P.; Rothenberger, A.; J. Wheatley, A.; S. Wright, D.; Choi, N.; McPartlin, M.; Stalke, D. Direct synthesis of heterocyclic $[(RP)E]^-$ anions using $[E(NMe_2)_3]$ (E = Sb, As); Implications to the Mechanism of Formation of Zintl Compounds. *Chem. Commun.* **1998**, (22), 2485–2486.
